# Design and synthesis of 5-aryl-4-(4-arylpiperazine-1-carbonyl)-2*H*-1,2,3-triazole derivatives as colchicine binding site inhibitors

**DOI:** 10.1038/s41598-017-17449-0

**Published:** 2017-12-07

**Authors:** Yue Wu, Dongjie Feng, Meiqi Gao, Zhiwei Wang, Peng Yan, Zhenzhen Gu, Qi Guan, Daiying Zuo, Kai Bao, Jun Sun, Yingliang Wu, Weige Zhang

**Affiliations:** 1 Key Laboratory of Structure-Based Drug Design and Discovery, Ministry of Education, Shenyang Pharmaceutical University, 103 Wenhua Road, Shenhe District Shenyang, 110016 China; 2 Department of Pharmacology, Shenyang Pharmaceutical University, 103 Wenhua Road, Shenhe District Shenyang, 110016 China; 3 Wuya College of Innovation, Shenyang Pharmaceutical University, 103 Wenhua Road, Shenhe District Shenyang, 110016 China; 4 Clinical Pharmacology Laboratory, Henan Province People’s Hospital, Zhengzhou University People’s Hospital, 7 Weiwu Road, Jinshui District Zhengzhou, 450003 China

## Abstract

A series of 5-aryl-4-(4-arylpiperazine-1-carbonyl)-2*H*-1,2,3-triazol derivatives were designed as potential microtubule targeting agents. The regioselective alkylation of 5-aryl-4-(4-arylpiperazine-1-carbonyl)-2*H*-1,2,3-triazole was predicted by computations and confirmed by an unambiguous synthetic route. The antiproliferative activity of the synthesized compounds was tested *in vitro* using three human cancer cell lines and some compounds exhibited significant antiproliferative activity, which suggested the reasonability of introduction of the 1,2,3-triazole fragment. Among them, compound **7p** showed highest activity with the IC_50_ values at nanomolar level towards all three cell lines, which were comparable to the positive control, CA-4. Tubulin polymerization assay, immunofluorescence studies, cell cycle analysis and competitive tubulin-binding assay strongly proved that **7p** is a colchicine binding site inhibitor of tubulin. Thus, **7p** was identified as a promising drug candidate for further development of colchicine binding site inhibitors.

## Introduction

**Figure 1:**
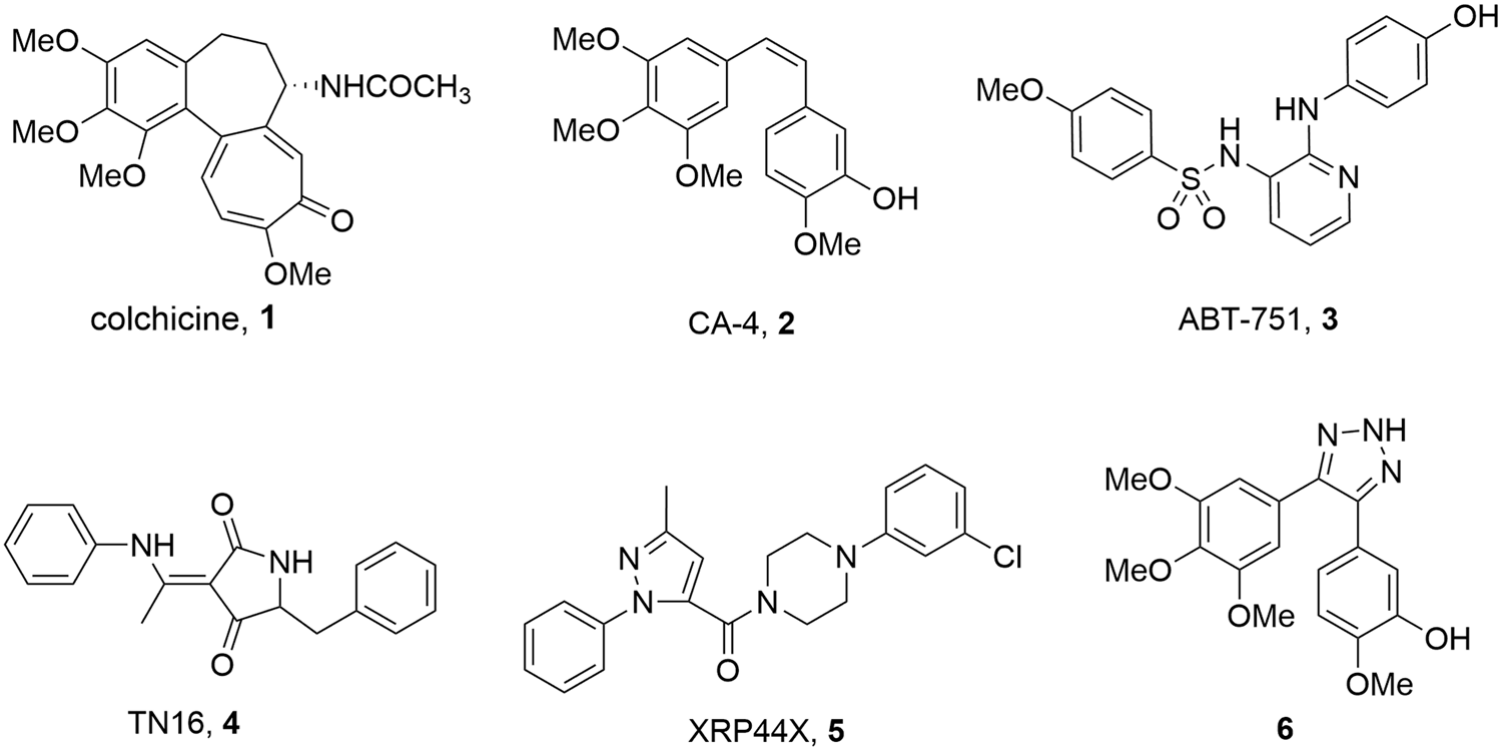
Structure of CBSIs.

Microtubules play crucial roles in cellular structure and various aspects of cell functions. The polymerization dynamics of microtubules are vital during mitosis, which is regarded as a promising target for cancer therapy since it can be profoundly affected by even small molecules^1^. Microtubule targeted agents can be classified into two major classes: microtubule-stabilizing agents which stimulate microtubule polymerization, such as paclitaxel (taxol); microtubule-destabilizing agents which inhibit polymerization of tubulin, include vinblastine and colchicine (**1**, Fig. 1).

Although colchicine can significantly inhibit the polymerization of tubulin, its therapeutic application is limited by the low therapeutic index. To overcome the disadvantages of colchicine, it is desired to find novel compounds that target at the colchicine binding site and exert their biological effects by inhibiting tubulin assembly and suppressing microtubule formation. Those compounds are classified as colchicine binding site inhibitors (CBSIs)^2^. Over decades, a large number of CBSIs have been reported, such as combretastatin A-4 (CA-4, **2**)^3^, ABT-751 (**3**)^4^ and TN16 (**4**)^5^. XRP44X (**5**)^6^ and other piperazine based CBSIs reported by Wasylyk and Chopra *et al*.^7^, showed potential effects on the inhibition of microtubules polymerization and the morphology of the actin cytoskeleton.

1,2,3-Triazoles attracted our attention since tremendous compounds containing this fragment have been reported to show potent antitumor activity^8,9^. For example, 4,5-disubstituted 1,2,3-triazole (**6**) was designed as an analogue of CA-4^10^, which showed low IC_50_ value at nanomolar level. Therefore, we designed a series of 5-aryl-4-(4-arylpiperazine-1-carbonyl)-2*H*-1,2,3-triazole derivatives (**7**) and 2-alkyl-5-aryl-4-(4-arylpiperazine-1-carbonyl)-2*H*-1,2,3-triazole derivatives (**8**) by replacing the pyrazole of XRP44X with 1,2,3-triazole. Meanwhile, those compounds showed well performance on molecular modeling studies. To access 2-alkylated derivatives, the regioselective alkylation of 5-aryl-4-(4-arylpiperazine-1-carbonyl)-2*H*-1,2,3-triazole was investigated by computational before actual synthesis. Antiproliferative activity of all compounds was evaluated, and several compounds with considerable activity had been found. The compound with the highest antiproliferative activity was thought as a colchicine binding site inhibitor by further investigation.

## Result and Discussion

### Molecular design

By replacing the pyrazole of XRP44X with 1,2,3-triazole, **7b** was designed. XRP44X possess a methyl group on the five-member ring, and previously study suggested that the methyl group was critical for bioactivity^11^. Thus, **8b** that possess a methyl group on N-2 of 1,2,3-triazole was designed.

**Figure 2:**
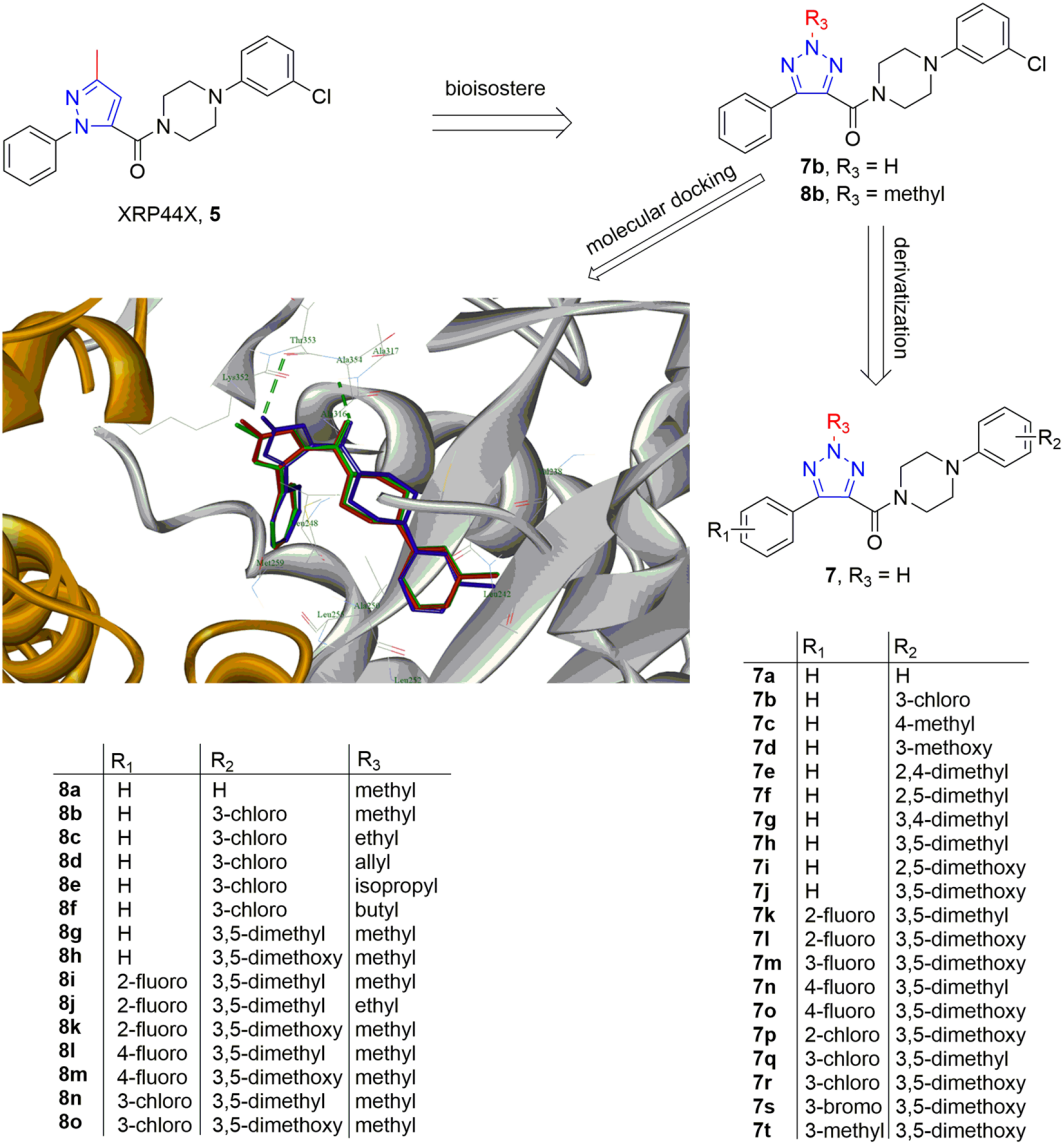
Design strategy of and docked pose of desired compounds. **5** (green), **7b** (blue) and **8b** (red) in the colchicine binding pocket (PDB code: 3HKC).

We further carried out the molecular docking for **7b** and **8b** (Fig. 2). Both of them were superimposed well with **5**. The carbonyl group contributes a hydrogen bond to β-Ala317 for these three ligands. Although **7b** lacked a methyl group, its 2*-*H on 1,2,3-triazoe ring could contribute a hydrogen bond to β-Thr353. It suggested the desired compounds are promising for inhibition of tubulin polymerization and a series of derivates (**7**, **8**) were further designed and synthesized for biological investigation.

### Chemistry

**Figure 3:**
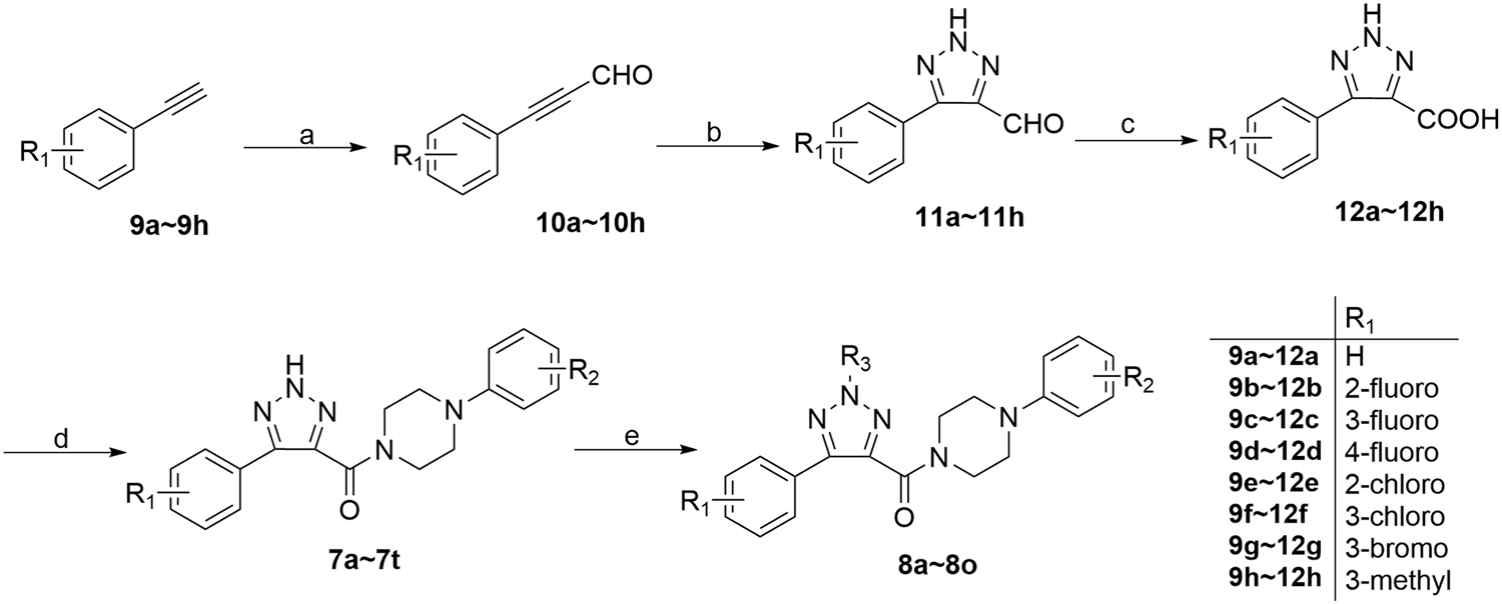
Synthetic route of all compounds. Reagents and conditions: (**a**) 1) *n*-BuLi (2.5 M in hexane), THF, −40 °C, 30 min; 2) DMF, −40 °C, 3) r.t., 1 h; (**b**) NaN_3_, DMSO, r.t., 30 min; (**c**) H_2_O_2_, KOH, MeOH, 30 min; (**d**) arylpiperazine, EDCI, HOBt, ethyl acetate, r.t., overnight; (**e**) (R_3_)_2_SO_4_, K_2_CO_3_, acetone, r.t., 1~3 h. or R_3_Br, K_2_CO_3_, KI, acetone, r.t., 2~4 h.

All desired compounds (**7**, **8**) were obtained by following the synthetic route outlined in Fig. 3. Commercially available arylacetylene derivatives (**9**) were used as the starting materials, which were transferred into arylpropiolaldehyde derivatives (10) by the formulation of acetylides with DMF^12^. Then, azide–alkyne 1,3-dipolar cycloadditions was carried out for obtaining 5-aryl-2*H*-1,2,3-triazole-4-carbaldehyde (**11)**, followed by oxidation into 5-aryl-2*H*-1,2,3-triazole-4-carboxylic acid (12) with hydrogen peroxide^13^. Finally, amidations were carried by EDCI and HOBt to generate the desired compounds **7a**-**7t**.

**Figure 4:**
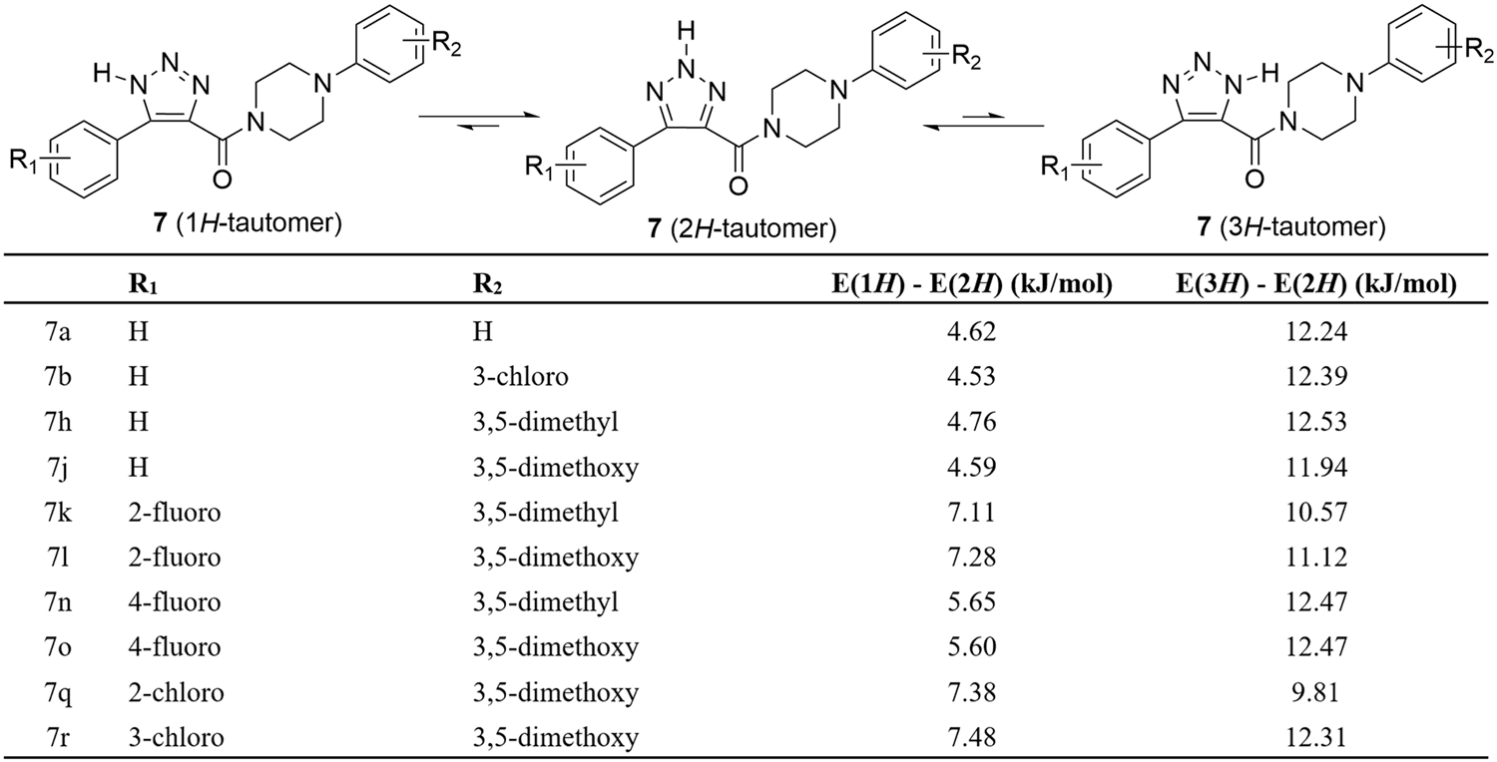
DFT computations of three tautomers of 7.

Direct alkylation of **7** may produce three different products since the tautomerism of 1,2,3-triazole. Previously studies suggested that similar substrates^14–16^ prone to generate N-2 alkylated products. In our case, density functional theory (DFT) computations were carried out for three tautomers of **7** to further predict the alkylated products. It showed that 2*H*-tautomers always have lowest energy in acetone. The results of computations were obtained for the all **7** substrates, as shown in Fig. 4. The energy of 2*H*-tautomers, lower than 1*H*- and 3*H*-tautomers, suggested N-2 alkylation products would be the principal products.

**Figure 5:**
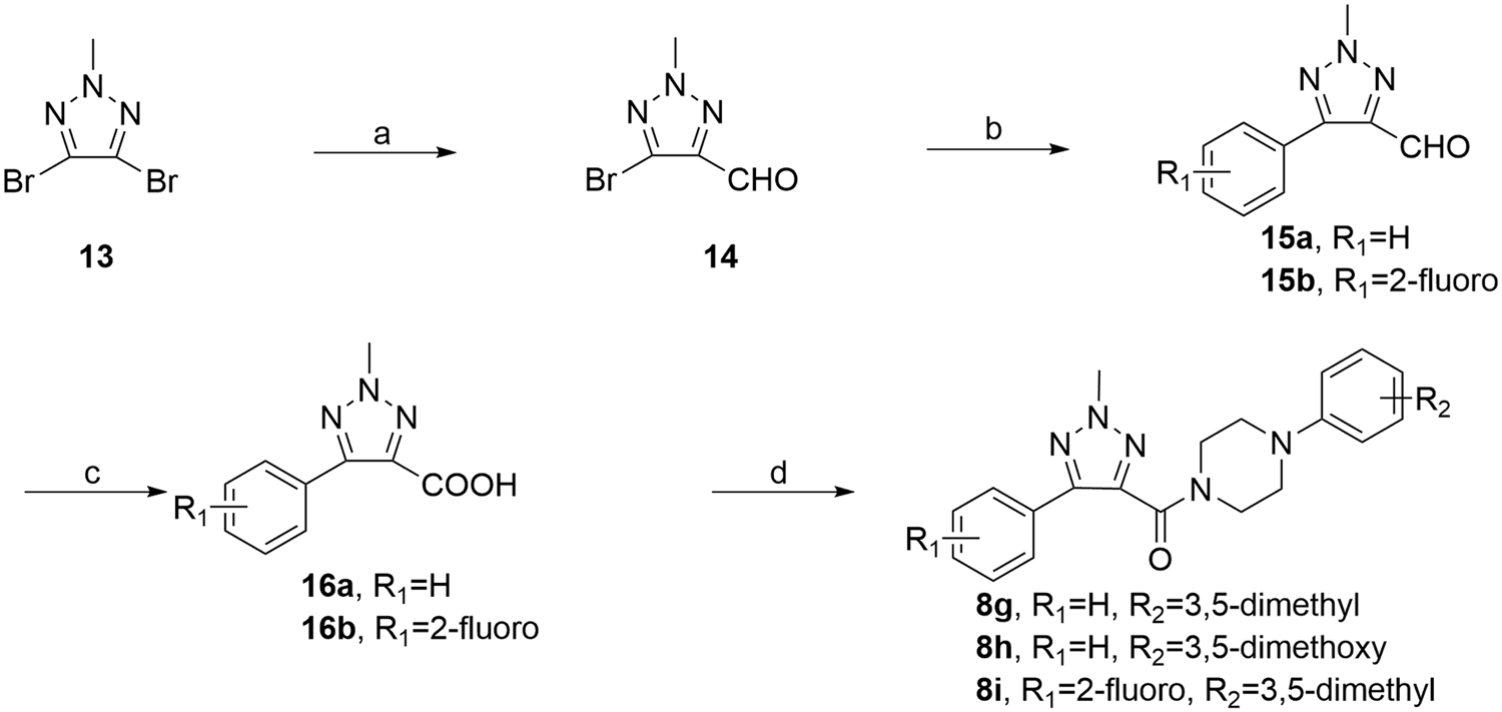
An unambiguous synthetic route for **8g**, **8h** and **8i**. Reagents and conditions: (**a**) 1) *i*-PrMgCl, THF, −45 °C, 30 min; 2) DMF, −45 °C, 15 min; (**b**) arylboric acid, Pd(PPh_3_)_4_, K_2_CO_3_, water/dioxane, 100 °C, 3 h; (**c**) H_2_O_2_, KOH, MeOH, 30 min; (**d**) arylpiperazine, EDCI, HOBt, ethyl acetate, r.t., overnight.

To further verify the structures of the alkylated products, we synthesized several representative compounds by an unambiguous synthetic route (Fig. 5). 4,5-Dibrome-2-methyl-2*H*-1,2,3-triazole (**13**) was synthesized by the reported methods^17^. Then, Grignard reaction was carried out to yield **14** ^18^. 5-Aryl was installed by Suzuki coupling to obtain **15** ^19^, followed by oxidation and amidation to generate **8g**, **8h**, and **8i**. These three compounds were proven as identical as the principal products obtained from the synthetic route in Fig. 3.

### *In vitro* antiproliferative activity and structure-activity relationship

The synthesized compounds (**7**, **8**) were investigated for their antiproliferative activity against cancer cells by MTT assay. Three human carcinoma cell lines including gastric adenocarcinoma SGC-7901 cells, lung adenocarcinoma A549 cells and fibrosarcoma HT-1080 cells were used. Two of the most well-known CBSIs, colchicine (**1**) and CA-4 (**2**) were evaluated as positive controls (Table 1).

**Table 1:** Antiproliferative activity of target compounds (7~8).

compound	Structure			IC_50_ (μM)		
	R_1_	R_2_	R_3_	SGC-7901	A549	HT-1080
**7a**	H	H	H	14.4 ± 0.6	49.0 ± 0.6	43.0 ± 0.9
**7b**	H	3-chloro	H	1.22 ± 0.03	1.30 ± 0.06	2.38 ± 0.04
**7c**	H	4-methyl	H	2.08 ± 0.04	13.2 ± 0.5	4.64 ± 0.19
**7d**	H	3-methoxy	H	0.704 ± 0.031	0.600 ± 0.022	1.37 ± 0.05
**7e**	H	2,4-dimethyl	H	5.12 ± 0.22	34.0 ± 0.9	6.64 ± 0.28
**7f**	H	2,5-dimethyl	H	82.2 ± 4.1	84.2 ± 3.2	25.9 ± 0.9
**7g**	H	3,4-dimethyl	H	8.04 ± 0.27	12.5 ± 0.2	4.75 ± 0.14
**7h**	H	3,5-dimethyl	H	0.243 ± 0.004	0.401 ± 0.016	0.580 ± 0.027
**7i**	**H**	**2,5-dimethoxy**	**H**	**0.368 ± 0.007**	**0.366 ± 0.018**	**0.254 ± 0.005**
**7j**	H	3,5-dimethoxy	H	0.107 ± 0.002	0.067 ± 0.001	0.050 ± 0.001
**7k**	2-fluoro	3,5-dimethyl	H	0.236 ± 0.005	0.215 ± 0.011	0.260 ± 0.005
**7l**	2-fluoro	3,5-dimethoxy	H	0.254 ± 0.003	0.236 ± 0.012	0.561 ± 0.017
**7m**	3-fluoro	3,5-dimethoxy	H	0.165 ± 0.008	0.191 ± 0.003	0.190 ± 0.008
**7n**	4-fluoro	3,5-dimethyl	H	0.477 ± 0.009	0.532 ± 0.019	0.980 ± 0.024
**7o**	4-fluoro	3,5-dimethoxy	H	0.493 ± 0.017	0.447 ± 0.02	1.05 ± 0.03
**7p**	**2-chloro**	**3,5-dimethoxy**	**H**	**0.052 ± 0.002**	**0.029 ± 0.0005**	**0.005 ± 0.0002**
**7q**	3-chloro	3,5-dimethyl	H	0.488 ± 0.016	0.508 ± 0.022	0.434 ± 0.012
**7r**	3-chloro	3,5-dimethoxy	H	0.509 ± 0.014	0.449 ± 0.02	0.389 ± 0.006
**7s**	3-bromo	3,5-dimethoxy	H	10.1 ± 0.3	11.2 ± 0.2	9.81 ± 0.47
**7t**	3-methyl	3,5-dimethoxy	H	0.739 ± 0.017	0.310 ± 0.014	0.352 ± 0.011
**8a**	H	H	methyl	10.8 ± 0.3	4.52 ± 0.12	4.45 ± 0.11
**8b**	H	3-chloro	methyl	1.05 ± 0.01	1.10 ± 0.05	0.841 ± 0.035
**8c**	H	3-chloro	ethyl	3.10 ± 0.15	14.4 ± 0.4	10.0 ± 0.2
**8d**	H	3-chloro	allyl	6.22 ± 0.17	81.0 ± 2.6	37.7 ± 1.2
**8e**	H	3-chloro	isopropyl	18.2 ± 0.6	46.8 ± 1.8	78.5 ± 2.7
**8f**	H	3-chloro	butyl	6.18 ± 0.14	10.0 ± 0.5	>100
**8g**	H	3,5-dimethyl	methyl	0.559 ± 0.014	0.882 ± 0.014	0.639 ± 0.019
**8h**	H	3,5-dimethoxy	methyl	0.442 ± 0.012	0.304 ± 0.009	0.515 ± 0.011
**8i**	**2-fluoro**	**3,5-dimethyl**	**methyl**	**0.228 ± 0.006**	**0.210 ± 0.008**	**1.07 ± 0.03**
**8j**	2-fluoro	3,5-dimethyl	ethyl	54.2 ± 1.4	60.9 ± 0.6	44.4 ± 0.9
**8k**	2-fluoro	3,5-dimethoxy	methyl	0.162 ± 0.003	0.172 ± 0.008	0.165 ± 0.003
**8l**	4-fluoro	3,5-dimethyl	methyl	0.396 ± 0.018	0.686 ± 0.01	0.712 ± 0.009
**8m**	4-fluoro	3,5-dimethoxy	methyl	0.291 ± 0.013	0.639 ± 0.024	0.564 ± 0.010
**8n**	3-chloro	3,5-dimethyl	methyl	0.422 ± 0.009	0.461 ± 0.007	0.464 ± 0.016
**8o**	3-chloro	3,5-dimethoxy	methyl	0.448 ± 0.013	0.453 ± 0.011	0.475 ± 0.017
**Colchicine (1)**				0.134 ± 0.004	0.137 ± 0.006	0.042 ± 0.0018
**CA-4 (2)**				0.048 ± 0.0009	0.034 ± 0.0006	0.007 ± 0.0001

At first, the effect of alkyl on N-2 of 1,2,3-triazole (R_3_) was evaluated. All methylated compounds showed no significant change on the activity compared with corresponding compounds without methyl (**7a** vs. **8a**, **7b** vs. **8b**, **7h** vs. **8g**, **7j** vs. **8h**, **7l** vs. **8k**, ect.). These results indicated that methyl on N-2 is unnecessary, however, activity severely declined as bulky alkyl group was introduced (**8c**-**8f** vs. **8b**, **8j** vs. **8i**). Then, the effect of substituents on the aryl ring linked to the piperazine fragment (R_2_) was investigated. Compared with **7b** (R_2_ = 3-chloro), both **7a** (R_2_ = H) and **7c** (R_2_ = 4-methyl) showed dramatically decreased activity, while **7d** (R_2_ = 3-methoxy) also show considerable activity, which indicated steric factor is crucial on R_2_ rather than electronegativity factor. Furthermore, di-meta-substituted compounds showed increased activity, for instance, **7j** (R_2_ = 3,5-dimethoxy) showed evident higher activity than **7d** (R_2_ = 3-methoxy). Finally, we investigated the effect of substituents on the aryl ring directly linked to 1,2,3-triazole core (R_1_) and found that the position and electronegativity had minimum effects on the activity. Among the designed compounds, **7p** (R_1_ = 2-chloro) showed an IC_50_ value of 5~52 nM towards all three cell lines, which was comparable to CA-4 (7~48 nM).

### Tubulin polymerization

**Figure 6:**
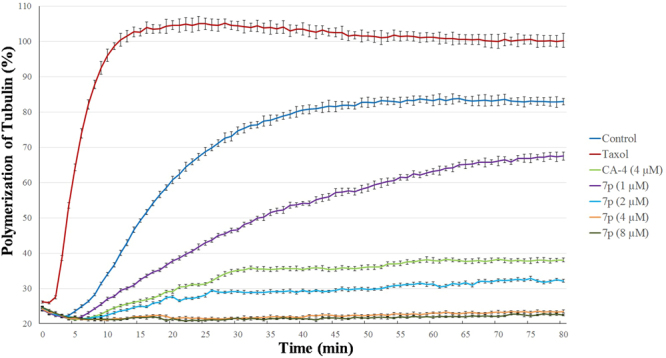
Effects of **7p** on tubulin polymerization. Tubulin had been pre-incubated for 1 min with **7p** at 1 μM, 2 μM, 4 μM and 8 μM, CA-4 at 4 μM, Taxol at 5 μM or vehicle DMSO.

**7p**, the compound with the highest antiproliferative activity, was assessed its effect on the tubulin polymerization *in vitro*. CA-4 (2) was used as a positive control and Taxol as a negative control. As shown in Fig. 6, **7p** exhibited a dose-dependent inhibition of tubulin polymerization. The inhibitory activity of **7p** towards tubulin polymerization (IC_50_ value, 2.06 µM) was more potent than the positive control CA-4.

### Immunofluorescence studies

**Figure 7:**
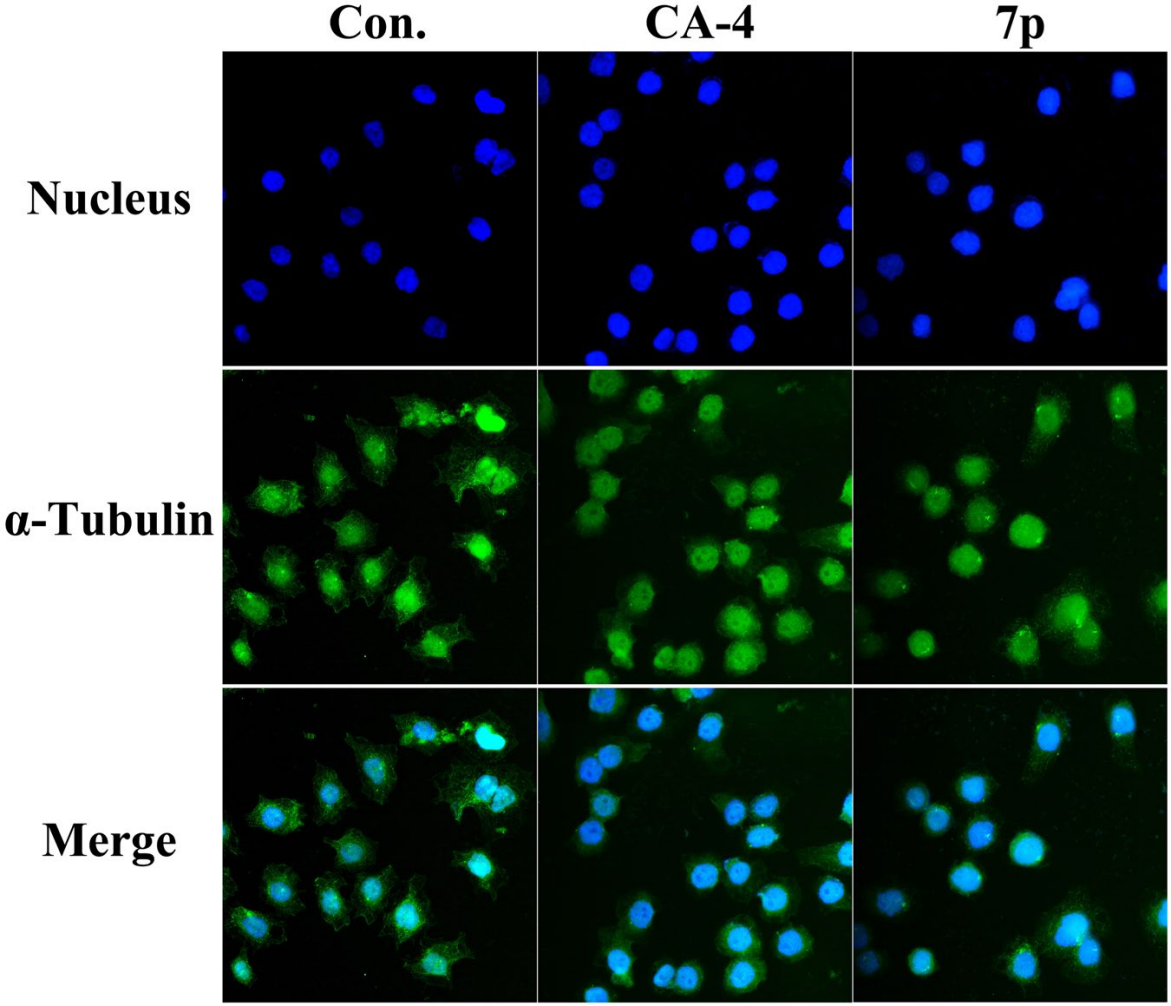
Immunofluorescence studies. CA-4 (48.1 nM) and **7p** (52.0 nM) induced depolymerization of the microtubule networks of SGC-7901 cancer cells, while untreated cells also used as control. After 24 h, cells were fixed and stained with anti-α-tubulin-FITC specific antibodies followed by DAPI. Microtubules and unassembled tubulin are shown in green, and nuclei, which were stained with DAPI, are shown in blue.

To visualize the effects of **7p** toward microtubule network, immunofluorescence studies were carried out. SGC-7901 cell lines were treated for 24 h with CA-4 and **7p** at their IC_50_. As shown in Fig. 7, both CA-4 and **7p** lead to radical changes in cell morphology of structured microtubules. These results indicated that **7p** can destabilize the microtubules of SGC-7901 cells.

### Cell cycle analysis

**Figure 8:**
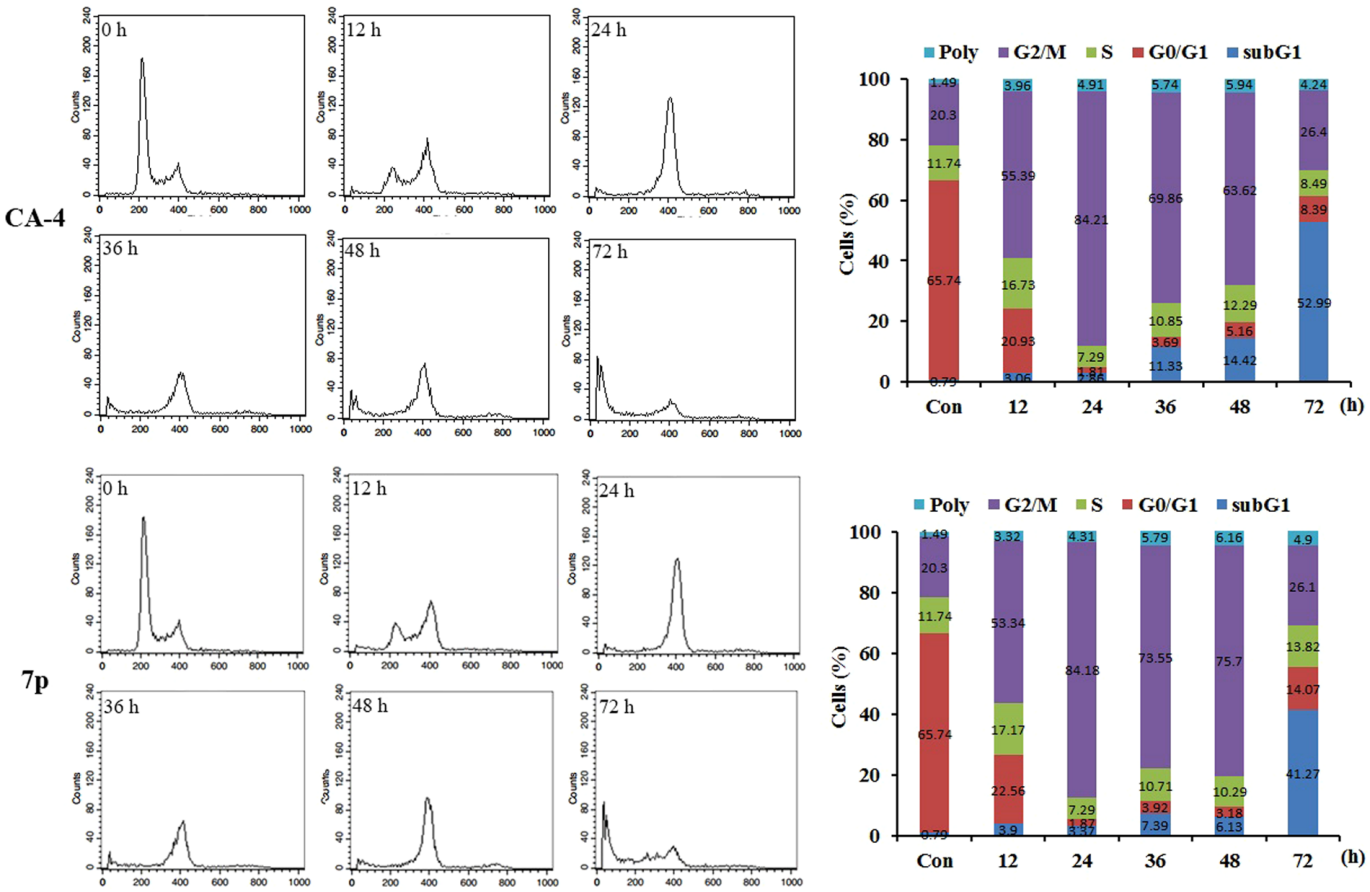
Effects of CA-4 and **7p** on cell cycle. SGC-7901 cells lines treated with CA-4 (48.1 nM) and **7p** (52.0 nM) for 0, 12, 24, 36, 48, 72 h.

To evaluate the effects of **7p** on the cell mitosis, we have analyzed the effect of **7p** on the cell cycle of SGC-7901 cells by flow cytometry (Fig. 8). CA-4 was also examined as a positive control. Similar as CA-4, **7p** arrested the cell cycle in G2/M phase significantly, increasing the percentage of cells in G2/M phase at 12 h and 24 h. Then, population of G2/M declined while the population of Sub-G1 raised, that indicated part of cells underwent apoptosis.

### Competitive tubulin-binding assay

**Figure 9:**
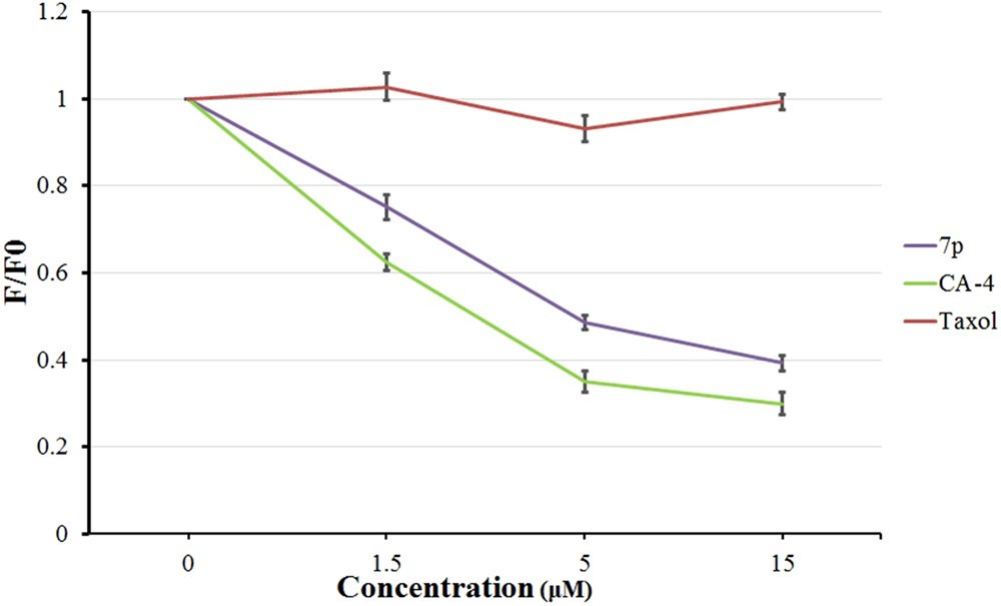
Fluorescence based colchicine competitive binding assay. The competitional binding between **7p** and colchicine was observed.

To confirm that **7p** binds to colchicine site of tubulin, we further assessed the competition between **7p** and colchicine for binding to tubulin via competitive binding assays (Fig. 9). CA-4 (2) and Taxol were used as the positive control and negative control, respectively^20^. The fluorescence of a colchicine-tubulin complex was reduced in the presence of **7p** in a dose-dependent manner, which indicated that **7p** binds at the colchicine binding site.

In this study, we designed and synthesized a series of 5-aryl-4-(4-arylpiperazine-1-carbonyl)-2*H*-1,2,3-triazole derivatives. The N-2 regioselective alkylation of 1,2,3-triazole was predicted by computations and confirmed by an unambiguous synthetic route. Most compounds showed potent antiproliferative activity at sub-micromolar or nanomolar, which suggested the reasonability of introduction of 1,2,3-triazole fragment. **7p** displayed highest activity against three human carcinoma cell lines, which was comparable to the positive control, CA-4. **7p** also highly inhibited tubulin polymerization *in vitro* with IC_50_ value was 2.06 µM. The immunofluorescence studies observed that **7p** induced depolymerization of the microtubule networks. Cell cycle analysis displayed evident G2/M arrest and induction of apoptosis. Furthermore, **7p** could compete with colchicine in tubulin binding site. All those experiments suggested that **7p** should be a colchicine binding site inhibitor of tubulin.

## Methods

### Reagents and equipment

All reagents were commercially available and were used without further purification. The silica gel plate (HSGF-254) and silica gel (H, 200–300 mesh) from Yantai Jiangyou silicone Development Co., Ltd. was used for preparative TLC and column chromatography, respectively. Visualization was made with UV light (254 nm and 365 nm). Mass spectra (MS) were obtained from Agilent Co. Ltd. on an Agilent 1100-sl mass spectrometer with an electrospray ionization source. ^1^H-NMR and ^13^C-NMR spectra were measured in CDCl_3_ or d_6_-DMSO with TMS as the internal reference on a Bruker AVANCE spectrometer operating at 400 MHz or 600 MHz (^1^H at 400 or 600 MHz, ^13^C at 100 MHz).

### Chemistry

The detailed information is in Supplementary Information.

### Cell line and culture conditions

The human gastric adenocarcinoma SGC-7901 cells, lung adenocarcinoma A549 cells and fibrosarcoma HT-1080 cells were purchased from Shanghai Institute of Cell Resources Center of Life Science (Shanghai, China). All cells were cultured in RPMI-1640 medium (Invitrogen, USA) supplemented with 10% fetal bovine serum (FBS; Hyclone, USA), streptomycin and penicillin at 37 °C in humidified atmosphere with 5% CO_2_.

### MTT assay

MTT assays were used to measure the cell viability after treatment. Briefly, 4~10 × 10^3^ cells/well were seeded in 96-well plates (Corning, NY, USA), cultured for 24 h, and treated with various concentrations of compounds for 72 h or incubated with SNP (10 mM) or Haemoglobin (10 mM) for 2 h, then treated with 4d (25 nM) for the indicated times. The DMSO concentration was kept below 0.05% in cell culture so it did not effect on cell growth. Then, MTT solution (5 mg/mL in PBS) was added (20 mL/well) to each well and incubated for another 4 h at 37 °C. The purple formazan crystals were dissolved in 100 mL dimethyl sulfoxide, and the plates were read on a plate reader (MK3, Thermo, German) at 492 nm. Experiments were repeated three times.

### Tubulin polymerization assay

*In vitro* tubulin polymerization assays were conducted as described in the manufacturer’s protocol (Cytoskeleton, Cat.#BK011P) using 96-well plates. Briefly, **7p**, CA-4 or Taxol were incubated with purified porcine tubulin (2 mg/mL) and buffer containing 10% glycerol and 1 mM GTP at 37 °C, and the effects of these compounds on tubulin polymerization were monitored kinetically for 82 min using a plate reader (Biotek Synergy HT, Winoo-skin, VT, USA). The increase in the relative fluorescence unit (RFU) was measured at an excitation of 340 ± 20 nm and emission of 415 ± 20 nm every minute. Experiments were repeated three times. The detail information of IC_50_ calculation is in Supplementary Information.

### Immunofluorenscence assay

One thousand SGC-7901 cells were cultured for 24 h in 96-well plates, followed by treatment with **7p**, CA-4 or 0.05% DMSO for the indicated times. After treatment, the cells were fixed with 4% formaldehyde in PBS, washed twice with PBS and permeabilised with 0.1% (v/v) Triton X-100 in PBS for 5 min. Then, cells were blocked with 5% bovine serum albumin (BSA) in PBS for 10 min. Microtubules were detected using a monoclonal anti-a-tubulin antibody diluted 1:50 in PBS overnight at 4 °C and a FITC conjugated secondary antibody diluted 1:200 in PBS for 1 h at 37 °C. After nuclear counter staining with DAPI (1 mg/mL), the cells were imaged using a fluorescence microscope. Experiments were repeated three times.

### Cell cycle analysis

One million SGC-7901 cells were incubated with **7p** (52 nM), CA-4 (48 nM) or 0.05% DMSO (control) for the indicated times. The cells were collected by centrifugation, washed with PBS and fixed in ice-cold 70% ethanol. The fixed cells were harvested by centrifugation and resuspended in 500 mL of PBS containing 50 mg/mL RNase. After 30 min incubation at 37 °C, cells were stained with 50 mg/mL PI at 4 °C in the dark for 30 min. Then, the samples were analysed by FACS can flow cytometry (BectoneDickinson, Franklin Lakes, NJ, USA). Experiments were repeated at least three times.

### Competitive tubulin-binding assay

**7p**, CA-4 and Taxol were carried out at various concentrations containing 5 μM of tubulin and colchicine for 60 min at 37 °C. Fluorescence values are normalized to DMSO (control), F/F_0_ represents inhibition rate (IR = F/F_0_) whereas F_0_ refers to fluorescence of the 5 μM colchicine–tubulin complex, and F describes the fluorescence of a given concentrations (1.5 μM, 5 μM and 15 μM) of **7p**, CA-4 and Taxol competition with the 5 μM colchicine–tubulin complex.

### DFT computations

The molecules was prepared by Avogadro (Version 1.20)^21^, and optimized by molecular dynamic forcefield MMFF94^22–26^. Then, all molecules were fully optimized by DFT basic set B3LYP/6-31 G(d) on Gaussian 09^27^. Solvent effects were computed using the CPCM^28,29^ model. After fully optimization, the energy of three tautomer was obtained for compare. The raw data with unit a.u. are transformed to kJ/mol (1 a.u. = 2625.5 kJ/mol). All date are provided on Supplementary Information.

### Molecular modeling studies

Molecular docking was carried out by Discovery Studio 3.5 with LibDock program. The 3D structure of 3HKC in docking study was downloaded from Protein Data Bank. The docking poses were selected according to previously studies^11^.

## Electronic supplementary material

Supplementary Information
