# Supplementary material for: Design and synthesis of 5-aryl-4-(4-arylpiperazine-1-carbonyl)-2H-1,2,3-triazole derivatives as colchicine binding site inhibitors
Source: Sci Rep. 2017 Dec 7;7:17120. doi: 10.1038/s41598-017-17449-0 (PMC5719359; doi:10.1038/s41598-017-17449-0)
Supplement: Supplementary file 1 — Supplementary Information [file 41598_2017_17449_MOESM1_ESM.pdf]

Design and synthesis of 5-aryl-4-(4-arylpiperazine-1-carbonyl)-2*H*-1,2,3-triazole derivatives as colchicine binding site inhibitors

Yue Wu[a], Dongjie Feng[a], Meiqi Gao[b], Zhiwei Wang[a], Peng Yan[a], Zhenzhen Gu[c], Qi Guan[a], Daiying Zuo[b], Kai Bao[c], Jun Sun[d*], Yingliang Wu[b*], Weige Zhang[a*]

[a] Key Laboratory of Structure-Based Drug Design and Discovery, Ministry of Education, Shenyang Pharmaceutical University, 103 Wenhua Road, Shenhe District, Shenyang 110016, China.

[b] Department of Pharmacology, Shenyang Pharmaceutical University, 103 Wenhua Road, Shenhe District, Shenyang 110016, China.

[c] Wuya College of Innovation, Shenyang Pharmaceutical University, 103 Wenhua Road, Shenhe District, Shenyang 110016, China.

[d] Clinical Pharmacology Laboratory, Henan Province People's Hospital, Zhengzhou University People's Hospital, 7 Weiwu Road, Jinshui District, Zhengzhou 450003, China

[*] corresponding authors.

e-mail: zhangweige@syphu.edu.cn (W. Zhang), yingliang_1016@163.com (Yingliang Wu), Jun_84@163.com (J. Sun).

List of content

Synthetic procedures for 10a-10h, 11a-11h, 12a-12h	1
Synthetic procedures for 7a-7t	4
Synthetic procedures for 8a-8o	10
An unambiguous synthetic route for 8g, 8h and 8i	15
^{1}H NMR and ^{13}C NMR spectra of the products	17
Information of DFT computation	54

Synthetic procedures for 10a-10h, 11a-11h, 12a-12h

General synthetic procedures for 3-arylpropiolaldehyde (**10a-10h**)

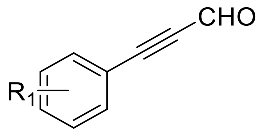

To a solution of 10 mmol of arylethyne (**9a-9h**) in 25 mL of THF that had been cooled to -40 °C under nitrogen was added 4 mL of n-BuLi (2.5 M in n-hexane) with stirring. After 15 min, to this solution was added DMF (0.85 mL, 11 mmol) dropwise over 10 min. The solution allowed to warm room temperature. After 30 min of further stirring, 80 mL of 10% KH_2PO_4 solution the solution was added at 0 °C. The solution was extract by ethyl acetate, organic layers were washed with brine, dried over anhydrous Na_2SO_4, filtered and concentrated under reduced pressure.

3-phenylpropiolaldehyde (**10a**). Colorless liquid, Yield 92%，MS (ESI) m/z 131.2 $[M+H]^+$, m/z 153.1 $[M+Na]^+$.

3-(2-fluorophenyl)propiolaldehyde (**10b**). Colorless liquid, Yield 87%，MS (ESI) m/z 149.0 $[M+H]^+$.

3-(3-fluorophenyl)propiolaldehyde (**10c**). liquid, Yield 86%，MS (ESI) m/z 149.0 $[M+H]^+$.

3-(4-fluorophenyl)propiolaldehyde (**10d**). Colorless liquid, Yield 88%，MS (ESI) m/z 149.0 $[M+H]^+$.

3-(2-chlorophenyl)propiolaldehyde (**10e**). Colorless liquid, Yield 81%，MS (ESI) m/z 165.0 $[M+H]^+$, m/z 187.0 $[M+Na]^+$.

3-(3-chlorophenyl)propiolaldehyde (**10f**). Colorless liquid, Yield 80%，MS (ESI) m/z 165.0 $[M+H]^+$, m/z 187.0 $[M+Na]^+$.

3-(3-bromophenyl)propiolaldehyde (**10g**). Colorless liquid, Yield 75%，MS (ESI) m/z 209.0 $[M+H]^+$.

3-(3-methylphenyl)propiolaldehyde (**10h**). Colorless liquid, Yield 88%，MS (ESI) m/z 145.1 $[M+H]^+$, m/z 167.1 $[M+Na]^+$.

General synthetic procedures for 5-aryl-2H-1,2,3-triazole-4-carbaldehyde (**11a-11h**)

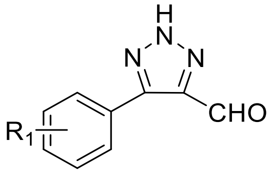

To a solution of 10 mmol of 3-arylpropiolaldehyde (**10a-10h**) in 25 mL of DMSO, NaN_3 (715 mg, 11 mmol) was added slowly. The reaction mixture was stirred for 30 min at room temperature, then, 80 mL of 10% KH_2PO_4 solution the solution was added. The solution was extract by ethyl acetate, organic layers were washed with brine, dried over anhydrous Na_2SO_4, filtered and concentrated under reduced pressure.

5-phenyl-2H-1,2,3-triazole-4-carbaldehyde (**11a)**. While soild, Yield 87%，MS (ESI) 174.0 $[M+H]^+$, m/z 196.0 $[M+Na]^+$, m/z 172.0 $[M-H]^-$.

5-(2-fluorophenyl)-2H-1,2,3-triazole-4-carbaldehyde (**11b)**. While soild, Yield 85%，MS (ESI) 192.0 $[M+H]^+$, m/z 214.0 $[M+Na]^+$, m/z 190.0 $[M-H]^-$.

5-(3-fluorophenyl)-2H-1,2,3-triazole-4-carbaldehyde (**11c)**. While soild, Yield 83%，MS (ESI) 192.0 $[M+H]^+$, m/z 214.0 $[M+Na]^+$, m/z 190.0 $[M-H]^-$.

5-(4-fluorophenyl)-2H-1,2,3-triazole-4-carbaldehyde (**11d)**. While soild, Yield 82%，MS (ESI) 192.0 $[M+H]^+$, m/z 214.0 $[M+Na]^+$, m/z 190.0 $[M-H]^-$.

5-(2-chlorophenyl)-2H-1,2,3-triazole-4-carbaldehyde (**11e)**. While soild, Yield 81%，MS (ESI) 208.0 $[M+H]^+$, m/z 230.0 $[M+Na]^+$, m/z 206.0 $[M-H]^-$.

5-(3-chlorophenyl)-2H-1,2,3-triazole-4-carbaldehyde (**11f)**. While soild, Yield 81%，MS (ESI) 208.0 $[M+H]^+$, m/z 230.0 $[M+Na]^+$, m/z 206.0 $[M-H]^-$.

5-(3-bromophenyl)-2H-1,2,3-triazole-4-carbaldehyde (**11g)**. While soild, Yield 79%，MS (ESI) 252.0 $[M+H]^+$, m/z 250.0 $[M-H]^-$.

5-(3-methylphenyl)-2H-1,2,3-triazole-4-carbaldehyde (**11h)**. While soild, Yield 83%，MS (ESI) 188.0 $[M+H]^+$, m/z 210.0 $[M+Na]^+$, m/z 186.0 $[M-H]^-$.

General synthetic procedures for 5-aryl-2H-1,2,3-triazole-4-carboxylic acid (**12a-12h**)

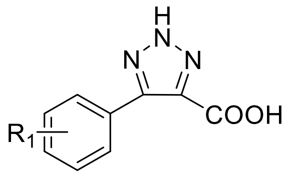

Aqueous hydrogen peroxide (30%, 8.0 mL, 80 mmol) was added dropwise to a stirred solution of 50% aq. KOH (2.24 g KOH and 2.24 mL water, 40 mmol) and 10 mmol of 5-aryl-2*H*-1,2,3-triazole-4-carbaldehyde (**11a-11h**) in methanol (25 mL) at 65 °C for 20 min. The mixture was then stirred at the same temperature for 10 min, cooled, acidified with 18% HCl to give 5-aryl-2*H*-1,2,3-triazole-4-carboxylic acid.

5-phenyl-2H-1,2,3-triazole-4-carboxylic acid (**12a)**. While soild, Yield 89%.

5-(2-fluorophenyl)-2H-1,2,3-triazole-4-carboxylic acid (**12b)**. While soild, Yield 87%.

5-(3-fluorophenyl)-2H-1,2,3-triazole-4-carboxylic acid (**12c)**. While soild, Yield 90%.

5-(4-fluorophenyl)-2H-1,2,3-triazole-4-carboxylic acid (**12d)**. While soild, Yield 91%.

5-(2-chlorophenyl)-2H-1,2,3-triazole-4-carboxylic acid (**12e)**. While soild, Yield 88%.

5-(3-chlorophenyl)-2H-1,2,3-triazole-4-carboxylic acid (**12f)**. While soild, Yield 86%.

5-(3-bromophenyl)-2H-1,2,3-triazole-4-carboxylic acid (**12g)**. While soild, Yield 86%.

5-(3-methylphenyl)-2H-1,2,3-triazole-4-carboxylic acid (**12h)**. While soild, Yield 87%.

Synthetic procedures for 7a-7t

General synthetic procedures for 5-aryl-4-(4-arylpiperazine-1-carbonyl)-2H-1,2,3-triazole (**7a-7t**)

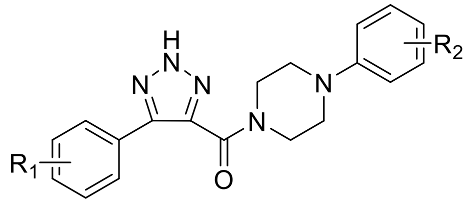

A solution of 1 mmol of 5-aryl-2*H*-1,2,3-triazole-4-carboxylic acid (**12a-12h**), 1-Ethyl-3-(3-dimethylaminopropyl)carbodiimide hydrochloride (192 mg, 1 mmol), 1-hydroxybenzotriazole (135 mg, 1 mmol) and 1 mmol of arylpiperazine, was stirred at room temperature overnight. After concentrated, the residual was purified by Preparative-TLC (DCM: MeOH = 25:1).

5-phenyl-4-(4-phenylpiperazine-1-carbonyl)-2H-1,2,3-triazole (**7a**). Yellow solid, yield: 69 %; ^{1}H NMR (400 MHz, DMSO-d_6) δ 7.71 (2H, d, *J* = 6.20 Hz), 7.47 (3H, m), 7.21 (2H, t, *J* = 7.94 Hz), 6.91 (2H, d, *J* = 8.17 Hz), 6.80 (1H, t, *J* = 7.28 Hz), 3.84 (2H, s), 3.50 (2H, s), 3.21 (2H, s), 2.94 (2H, s). ^{13}C NMR (150 MHz, $CDCl_3$) δ 163.01, 150.6, 143.3, 136.99, 129.18 (2C), 128.84 (2C), 128.67, 128.31, 127.44 (2C), 120.67, 116.74 (2C), 49.51, 49.3, 46.98, 42.25; HRMS calcd. for $C_{19}H_{20}N_5O$ $[M+H]^+$ 334.1668, found 334.1771, calcd. for $C_{19}H_{18}N_5O$ $[M-H]^-$ 332.1517, found 332.1539.

5-phenyl-4-(4-(3-chlorophenyl)piperazine-1-carbonyl)-2H-1,2,3-triazole (**7b**). Yellow solid, yield: 72 %; ^{1}H NMR (400 MHz, $CDCl_3$) δ 7.67 (2H, d, *J* = 6.20 Hz), 7.35 (3H, m), 7.14 (1H, t, *J* = 8.13 Hz), 6.83 (1H, dd, *J* = 7.89 Hz, 1.60 Hz), 6.79 (1H, t, *J* = 1.95 Hz), 3.96 (2H, t, *J* = 4.65 Hz), 3.47 (2H, m), 3.22 (2H, t, *J* = 4.77 Hz), 2.90 (2H, t, *J* = 4.28 Hz). ^{13}C NMR (150 MHz, $CDCl_3$) δ 162.78, 151.73, 145.17, 137.25, 134.99, 130.13, 129.25, 128.90 (2C), 128.32, 127.50 (2C), 120.26, 116.46, 114.52, 49.06, 48.80, 46.75, 42.01; HRMS calcd. for $C_{19}H_{19}N_5OCl$ $[M+H]^+$ 368.1273, found 368.128, calcd. for $C_{19}H_{17}N_5OCl$ $[M-H]^-$ 366.1127, found 366.1135.

5-phenyl-4-(4-(4-methylphenyl)piperazine-1-carbonyl)-2H-1,2,3-triazole (**7c**). Yellow solid, yield: 77 %; ^{1}H NMR (600 MHz, $CDCl_3$) δ 7.70 (2H, dd, *J* = 8.41 Hz, 1.45 Hz), 7.40 (3H, m), 7.08 (2H, d, *J* = 8.27 Hz), 6.80 (2H, d, *J* = 8.55 Hz), 4.00 (2H, t, *J* = 5.07 Hz), 3.49 (2H, t, *J* = 4.93 Hz), 3.20

(2H, t, J = 5.10 Hz), 2.89 (2H, t, J = 5.00 Hz), 2.28 (3H, s). ^{13}C NMR (150 MHz, $CDCl_3$) δ 162.74, 148.50, 143.77, 137.34, 130.45, 129.76 (2C), 129.25, 128.91 (2C), 128.48, 127.49 (2C), 117.18 (2C), 50.17, 50.01, 47.02, 42.24, 20.43; HRMS calcd. for $C_{20}H_{22}N_5O$ $[M+H]^+$ 348.1819, found 348.1854, calcd. for $C_{20}H_{20}N_5O$ $[M-H]^-$ 346.1673, found 346.1681.

5-phenyl-4-(4-(3-methoxyphenyl)piperazine-1-carbonyl)-2H-1,2,3-triazole (**7d**). Yellow solid, yield: 73 %; ^{1}H NMR (600 MHz, $CDCl_3$) δ 7.69 (2H, dd, J = 8.27 Hz, 1.41 Hz), 7.36 (3H, m), 7.15 (2H, t, J = 8.20 Hz), 6.45 (2H, td, J = 9.03 Hz, 2.11 Hz), 6.39 (1H, t, J = 2.24 Hz), 3.97 (2H, t, J = 5.03 Hz), 3.76 (3H, s), 3.46 (2H, t, J = 4.93 Hz), 3.23 (2H, t, J = 5.07 Hz), 2.91 (2H, t, J = 4.38 Hz). ^{13}C NMR (150 MHz, $CDCl_3$) δ 162.90, 160.47, 151.95, 143.26, 136.93, 129.87, 129.19, 128.86 (2C), 128.31, 127.42 (2C), 109.37, 105.36, 103.18, 55.14, 49.38, 49.17, 46.88, 42.15; HRMS calcd. for $C_{20}H_{22}N_5O_2$ $[M+H]^+$ 364.1768, found 364.1882, calcd. for $C_{20}H_{20}N_5O_2$ $[M-H]^-$ 362.1622, found 362.1705.

5-phenyl-4-(4-(2,4-dimethylphenyl)piperazine-1-carbonyl)-2H-1,2,3-triazole (**7e**). Yellow solid, yield: 68 %; ^{1}H NMR (400 MHz, $CDCl_3$) δ 7.72 (2H, dd, J = 7.89 Hz, 1.41 Hz), 7.40 (3H, m), 6.99 (1H, s), 6.94 (1H, d, J = 8.12 Hz), 6.78 (1H, d, J = 8.03 Hz), 3.97 (2H, s), 3.44 (2H, t, J = 4.39 Hz), 2.91 (2H, t, J = 4.79 Hz), 2.61 (2H, t, J = 4.46 Hz), 2.26 (3H, s), 2.24 (3H, s). ^{13}C NMR (150 MHz, $CDCl_3$) δ 162.96, 148.14, 143.44, 137.45, 133.34, 132.56, 131.83, 129.13, 128.88 (2C), 128.30, 127.47 (2C), 127.09, 119.13, 51.86, 51.68, 47.74, 42.84, 20.64, 17.55; HRMS calcd. for $C_{21}H_{24}N_5O$ $[M+H]^+$ 362.1975, found 362.1989, calcd. for $C_{21}H_{22}N_5O$ $[M-H]^-$ 360.183, found 360.1835.

5-phenyl-4-(4-(2,5-dimethylphenyl)piperazine-1-carbonyl)-2H-1,2,3-triazole (**7f**). Yellow solid, yield: 73 %; ^{1}H NMR (400 MHz, $CDCl_3$) δ 7.72 (2H, dd, J = 8.08, 1.50 Hz), 7.40 (3H, m), 7.05 (1H, d, J = 7.61 Hz), 6.82 (2H, d, J = 7.51 Hz), 3.98 (2H, s), 3.46 (2H, t, J = 4.58 Hz), 2.94 (2H, t, J = 4.89 Hz), 2.65 (2H, t, J = 4.68 Hz), 2.28 (3H, s), 2.22 (3H, s). ^{13}C NMR (150 MHz, $CDCl_3$) δ 162.97, 150.40, 143.43, 136.20, 130.91, 129.55, 129.33, 129.12, 128.89 (2C), 128.29, 127.46 (2C), 124.43, 119.98, 51.67, 51.49, 47.74, 42.84, 21.07, 17.28; HRMS calcd. for $C_{21}H_{24}N_5O$ $[M+H]^+$ 362.1975, found 362.2036, calcd. for $C_{21}H_{22}N_5O$ $[M-H]^-$ 360.183, found 360.1842.

5-phenyl-4-(4-(3,4-dimethylphenyl)piperazine-1-carbonyl)-2H-1,2,3-triazole (**7g**). Yellow solid, yield: 68 %; ^{1}H NMR (400 MHz, $CDCl_3$) δ 7.68 (2H, dd, *J* = 7.76 Hz, 1.50 Hz), 7.35 (3H, m), 7.01 (1H, d, *J* = 8.23 Hz), 6.69 (1H, d, *J* = 2.16 Hz), 6.62 (1H, dd, *J* = 8.26 Hz, 2.36 Hz), 3.99 (2H, t, *J* = 4.87 Hz), 3.47 (2H, t, *J* = 4.65 Hz), 3.18 (2H, t, *J* = 4.86 Hz), 2.86 (2H, t, *J* = 4.70 Hz), 2.21 (3H, s), 2.18 (3H, s). ^{13}C NMR (150 MHz, $CDCl_3$) δ 162.77, 148.88, 143.57, 137.30, 130.22, 129.25, 129.20, 128.89 (2C), 128.51, 128.30, 127.45 (2C), 118.83, 114.51, 50.17, 50.03, 47.04, 42.25, 20.09, 18.76; HRMS calcd. for $C_{21}H_{24}N_5O$ $[M+H]^+$ 362.1975, found 362.211, calcd. for $C_{21}H_{22}N_5O$ $[M-H]^-$ 360.183, found 360.1853.

5-phenyl-4-(4-(3,5-dimethylphenyl)piperazine-1-carbonyl)-2H-1,2,3-triazole (**7h**). Yellow solid, yield: 74 %; ^{1}H NMR (400 MHz, $CDCl_3$) δ 7.70 (2H, dd, *J* = 7.94 Hz, 1.46 Hz), 7.37 (3H, m), 6.57 (1H, s), 6.52 (2H, s), 3.99 (2H, s), 3.48 (2H, s), 3.23 (2H, t, *J* = 4.89 Hz), 2.92 (2H, s), 2.26 (6H, s). ^{13}C NMR (150 MHz, $CDCl_3$) δ 163.20, 150.69, 142.97, 138.67 (2C), 136.85, 128.99, 128.77 (2C), 128.41, 127.38 (2C), 122.52, 114.66 (2C), 49.59, 49.39, 46.99, 42.24, 21.48 (2C); HRMS calcd. for $C_{21}H_{24}N_5O$ $[M+H]^+$ 362.1975, found 362.2053, calcd. for $C_{21}H_{22}N_5O$ $[M-H]^-$ 360.183, found 360.1871.

5-phenyl-4-(4-(2,5-dimethoxyphenyl)piperazine-1-carbonyl)-2H-1,2,3-triazole (**7i**). Yellow solid, yield: 72 %; ^{1}H NMR (600 MHz, $CDCl_3$) δ 7.70 (2H, dd, *J* = 8.34 Hz, 1.45 Hz), 7.37 (3H, m), 6.76 (2H, d, *J* = 8.82 Hz), 6.51 (2H, dd, *J* = 8.82 Hz, 2.90 Hz), 6.42 (1H, t, *J* = 2.90 Hz), 4.01 (2H, t, *J* = 4.69 Hz), 3.79 (3H, s), 3.74 (3H, s), 3.48 (2H, t, *J* = 4.83 Hz), 3.11 (2H, t, *J* = 4.89 Hz), 2.79 (2H, t, *J* = 4.76 Hz). ^{13}C NMR (150 MHz, $CDCl_3$) δ 162.85, 153.96, 146.47, 143.46, 141.29, 137.15, 129.12, 128.87 (2C), 128.56, 127.43 (2C), 111.83, 106.52, 106.15, 55.81, 55.57, 50.47, 50.29, 47.23, 42.38; HRMS calcd. for $C_{21}H_{24}N_5O_3$ $[M+H]^+$ 394.1874, found 394.1904, calcd. for $C_{21}H_{22}N_5O_3$ $[M-H]^-$ 392.1728, found 392.1749.

5-phenyl-4-(4-(3,5-dimethoxyphenyl)piperazine-1-carbonyl)-2H-1,2,3-triazole (**7j**). Yellow solid, yield: 71 %; ^{1}H NMR (400 MHz, $CDCl_3$) δ 7.71 (2H, dd, *J* = 7.94 Hz, 1.58 Hz), 7.38 (3H, m), 6.04 (1H, m), 6.02 (2H, m), 3.97 (2H, t, *J* = 4.95 Hz), 3.75 (6H, s), 3.46 (2H, t, *J* = 4.58 Hz), 3.23 (2H, t, *J* = 4.93 Hz), 2.92 (2H, t, *J* = 4.70 Hz). ^{13}C NMR (150 MHz, $CDCl_3$) δ 163.00, 161.33 (2C), 152.48, 142.97, 136.80, 129.11, 128.78 (2C), 128.18, 127.36 (2C), 95.54 (2C), 92.26, 55.13 (2C),

49.28, 49.07, 46.82, 42.11; HRMS calcd. for $C_{21}H_{24}N_5O_3$ $[M+H]^+$ 394.1874, found 394.1904, calcd. for $C_{21}H_{22}N_5O_3$ $[M-H]^-$ 392.1728, found 392.1766.

5-(2-fluorophenyl)-4-(4-(3,5-dimethylphenyl)piperazine-1-carbonyl)-2H-1,2,3-triazole (**7k**). Yellow solid, yield: 66 %; ^{1}H NMR (600 MHz, $CDCl_3$) δ 7.65 (1H, td, *J* = 7.42 Hz, 1.38 Hz), 7.35 (qd, 1H, *J* = 6.95 Hz, 1.33 Hz), 7.16 (1H, t, *J* = 7.58 Hz), 7.10 (1H, t, *J* = 9.43 Hz), 6.57 (1H, s), 6.55 (2H, s), 3.96 (2H, t, 4.42 Hz), 3.67 (2H, t, 4.34 Hz), 3.25 (2H, t, *J* = 4.81 Hz), 3.05 (2H, t, *J* = 4.53 Hz), 2.28 (6H, s). ^{13}C NMR (150 MHz, $CDCl_3$) δ 162.37, 160.41, 158.75, 150.88, 141.68, 140.38, 138.82 (2C), 131.06, 131.00, 130.32, 130.31, 124.61, 124.59, 122.61, 117.75, 116.03, 115.88, 114.76 (2C), 49.78, 49.57, 47.10, 42.19, 21.56 (2C); HRMS calcd. for $C_{21}H_{23}N_5OF$ $[M+H]^+$ 380.1881, found 380.1924, calcd. for $C_{21}H_{21}N_5OF$ $[M-H]^-$ 378.1736, found 378.1744.

5-(2-fluorophenyl)-4-(4-(3,5-dimethoxyphenyl)piperazine-1-carbonyl)-2H-1,2,3-triazole (**7l**). Yellow solid, yield: 69 %; ^{1}H NMR (600 MHz, $CDCl_3$) δ 7.73 (1H, td, *J* = 7.25 Hz, 1.32 Hz), 7.39 (1H, m), 7.23 (1H, t, *J* = 7.59 Hz), 7.13 (1H, t, *J* = 9.45 Hz), 6.07 (2H, m), 6.05 (1H, m), 3.94 (2H, t, *J* = 4.86 Hz), 3.77 (6H, s), 3.49 (2H, s), 3.26 (2H, t, *J* = 4.79 Hz), 3.06 (2H, t, *J* = 4.83 Hz). ^{13}C NMR (150 MHz, $CDCl_3$) δ 162.22, 161.47 (2C), 159.59 (d, *J* = 248.26 Hz), 152.75, 139.22, 138.80, 131.11 (d, *J* = 8.16 Hz), 130.31 (d, *J* = 2.38 Hz), 129.88, 124.65, 116.69 (d, *J* = 13.66 Hz), 115.98 (d, *J* = 21.42 Hz), 95.68 (2C), 92.34, 55.25 (2C), 49.46, 49.27, 46.94, 42.07; HRMS calcd. for $C_{21}H_{23}N_5O_3F$ $[M+H]^+$ 412.1779, found 412.1813, calcd. for $C_{21}H_{21}N_5O_3F$ $[M-H]^-$ 410.1634, found 410.1659.

5-(3-fluorophenyl)-4-(4-(3,5-dimethoxyphenyl)piperazine-1-carbonyl)-2H-1,2,3-triazole (**7m**). Yellow solid, yield: 67 %; ^{1}H NMR (600 MHz, $CDCl_3$) δ 7.74 (2H, m), 7.33 (1H, m), 7.03 (1H, td, *J* = 8.24 Hz, 1.93 Hz), 6.04 (3H, m), 3.98 (2H, t, *J* = 5.00 Hz), 3.77 (6H, s), 3.49 (2H, s), 3.25 (2H, t, *J* = 4.54 Hz), 2.98 (2H, t, *J* = 4.48 Hz). ^{13}C NMR (150 MHz, $CDCl_3$) δ 162.75(d, *J* = 244.92 Hz), 162.75, 161.39 (2C), 152.51, 142.81, 137.17, 130.72(d, *J* = 7.81 Hz), 130.43(d, *J* = 8.17 Hz), 123.04(d, *J* = 2.29 Hz), 115.94(d, *J* = 20.94 Hz), 114.34(d, *J* = 23.04 Hz), 95.65 (2C), 92.39, 55.17 (2C), 49.51, 49.18, 46.88, 42.18; HRMS calcd. for $C_{21}H_{23}N_5O_3F$ $[M+H]^+$ 412.1779, found 412.1807, calcd. for $C_{21}H_{21}N_5O_3F$ $[M-H]^-$ 410.1634, found 410.1739.

5-(4-fluorophenyl)-4-(4-(3,5-dimethylphenyl)piperazine-1-carbonyl)-2H-1,2,3-triazole (**7n**) .Yellow solid, yield: 73 %; ^{1}H NMR (600 MHz, $CDCl_3$) δ 7.69 (2H, m), 7.06 (2H, m), 6.57 (1H, s), 6.51 (2H, s), 3.98 (2H, t, *J* = 4.99 Hz), 3.53 (2H, t, *J* = 4.63 Hz), 3.23 (2H, t, *J* = 4.99 Hz), 2.97 (2H, t, *J* = 5.06 Hz), 2.26 (6H, s). ^{13}C NMR (150 MHz, $CDCl_3$) δ 163.43 (d, *J* = 248.55 Hz), 162.30, 150.66, 143.07, 138.81 (2C), 136.90, 129.38 (2C, d, *J* = 8.27 Hz), 124.67, 122.78, 115.91 (2C, d, *J* = 21.93 Hz), 114.79 (2C), 49.88, 49.57, 47.13, 42.59, 21.49 (2C); HRMS calcd. for $C_{21}H_{23}N_5OF$ $[M+H]^+$ 380.1881, found 380.1912, calcd. for $C_{21}H_{21}N_5OF$ $[M-H]^-$ 378.1736, found 378.1763.

5-(4-fluorophenyl)-4-(4-(3,5-dimethoxyphenyl)piperazine-1-carbonyl)-2H-1,2,3-triazole (**7o**). Yellow solid, yield: 74 %; ^{1}H NMR (600 MHz, $CDCl_3$) δ 7.67 (m, 2H), 7.03 (t, 2H, *J* = 8.61 Hz), 6.02 (s, 3H), 3.94 (s, 2H), 3.73 (s, 6H), 3.51 (s, 2H), 3.22 (s, 2H), 2.98 (s, 2H). ^{13}C NMR (150 MHz, $CDCl_3$) δ 162.96 (d, *J* = 248.16 Hz), 162.74, 161.32 (2C), 152.43, 142.71, 136.74, 129.30 (2C, d, *J* = 8.25 Hz), 124.58, 115.75 (2C, d, *J* = 21.54 Hz), 95.53 (2C), 92.25, 55.09 (2C), 49.44, 49.10, 46.83, 42.42; HRMS calcd. for $C_{21}H_{23}N_5O_3F$ $[M+H]^+$ 412.1779, found 412.1806, calcd. for $C_{21}H_{21}N_5O_3F$ $[M-H]^-$ 410.1634, found 410.1753.

5-(2-chlorophenyl)-4-(4-(3,5-dimethoxyphenyl)piperazine-1-carbonyl)-2H-1,2,3-triazole (**7p**). Yellow solid, yield: 74 %; ^{1}H NMR (600 MHz, $CDCl_3$) δ 7.55 (1H, dd, *J* = 7.23 Hz, 2.15 Hz), 7.47 (1H, m), 7.40 (2H, m), 6.05 (3H, s), 3.88 (2H, t, *J* = 4.48 Hz), 3.77 (6H, s), 3.75 (2H, s), 3.21 (2H, s), 3.00 (2H, s). ^{13}C NMR (150 MHz, $CDCl_3$) δ 161.88, 161.44 (2C), 152.75, 143.41, 139.13, 131.58, 131.17, 130.46, 129.87, 127.02, 124.27, 95.57 (2C), 92.25, 55.24 (2C), 49.51, 49.21, 46.87, 42.06; HRMS calcd. for $C_{21}H_{23}N_5O_3Cl$ $[M+H]^+$ 428.1484, found 428.1553, calcd. for $C_{21}H_{21}N_5O_3Cl$ $[M-H]^-$ 426.1338, found 426.1365.

5-(3-chlorophenyl)-4-(4-(3,5-dimethylphenyl)piperazine-1-carbonyl)-2H-1,2,3-triazole (**7q**). Yellow solid, yield: 71 %; ^{1}H NMR (600 MHz, $CDCl_3$) δ 7.75 (1H, s), 7.60 (1H, m), 7.30 (2H, m), 6.56 (1H, s), 6.51 (2H, s), 3.98 (2H, t, *J* = 4.85 Hz), 3.49 (2H, t, *J* = 4.70 Hz), 3.23 (2H, t, *J* = 4.92 Hz), 2.96 (2H, t, *J* = 5.35 Hz), 2.25 (6H, s). ^{13}C NMR (150 MHz, $CDCl_3$) δ 162.63, 150.61, 142.57, 138.68 (2C), 137.20, 134.58, 130.04, 129.42, 128.96, 127.30, 125.44, 122.65, 114.73 (2C), 49.80, 49.43, 47.02, 42.28, 21.42 (2C); HRMS calcd. for $C_{21}H_{23}N_5OCl$ $[M+H]^+$ 396.1586, found 396.1618, calcd. for $C_{21}H_{21}N_5OCl$ $[M-H]^-$ 394.144, found 394.1517.

5-(3-chlorophenyl)-4-(4-(3,5-dimethoxyphenyl)piperazine-1-carbonyl)-2H-1,2,3-triazole (**7r**). Yellow solid, yield: 69 %; ^{1}H NMR (600 MHz, $CDCl_3$) δ 7.73 (1H, m), 7.59 (1H, m), 7.29 (2H, d, *J* = 4.92 Hz), 6.02 (3H, s), 4.24 (3H, s), 3.95 (2H, t, *J* = 4.85 Hz), 3.73 (6H, s), 3.48 (2H, s), 3.23 (2H, t, *J* = 4.77 Hz), 2.96 (2H, s). ^{13}C NMR (150 MHz, $CDCl_3$) δ 162.50, 161.32 (2C), 152.49, 142.59, 137.24, 134.52, 130.47, 130.03, 128.94, 127.30, 125.46, 95.55 (2C), 92.31, 55.12 (2C), 49.45, 49.10, 46.80, 42.08; HRMS calcd. for $C_{21}H_{23}N_5O_3Cl$ $[M+H]^+$ 428.1484, found 428.1532, calcd. for $C_{21}H_{21}N_5O_3Cl$ $[M-H]^-$ 426.1338, found 426.1386.

5-(3-bromophenyl)-4-(4-(3,5-dimethoxyphenyl)piperazine-1-carbonyl)-2H-1,2,3-triazole (**7s**). Yellow solid, yield: 66 %; ^{1}H NMR (600 MHz, $CDCl_3$) δ 7.71 (2H, d, *J* = 6.96 Hz), 7.37 (3H, m), 6.04 (1H, s), 6.02 (2H, s), 3.96 (2H, s), 3.75 (6H, s), 3.46 (2H, s), 3.23 (2H, s), 2.92 (2H, s). ^{13}C NMR (150 MHz, $CDCl_3$) δ 162.79, 161.43 (2C), 153.39, 143.25, 139.83, 130.25, 129.64, 129.13, 128.80, 127.46, 125.22, 95.62 (2C), 92.36, 55.22 (2C), 49.49, 49.28, 46.81, 42.04; MS (ESI) m/z 472.1 $[M+H]^+$, m/z 470.1 $[M-H]^-$.

5-(3-methylphenyl)-4-(4-(3,5-dimethoxyphenyl)piperazine-1-carbonyl)-2H-1,2,3-triazole (**7t**). Yellow solid, yield: 70 %; ^{1}H NMR (600 MHz, $CDCl_3$) δ 7.52 (1H, s), 7.48 (1H, d, *J* = 7.77 Hz), 7.28 (2H, m), 6.04 (1H, m), 6.02 (2H, m), 3.96 (2H, t, *J* = 4.96 Hz), 3.75 (6H, s), 3.45 (2H, t, *J* = 4.76 Hz), 3.23 (2H, t, *J* = 5.03 Hz), 2.92 (2H, t, *J* = 4.83 Hz), 2.34 (3H, s). ^{13}C NMR (150 MHz, $CDCl_3$) δ 162.87, 161.36 (2C), 152.57, 143.16, 138.50, 130.88, 129.94, 128.73, 128.18, 127.96, 124.49, 95.58 (2C), 92.31, 55.16 (2C), 49.34, 49.15, 46.77, 42.05, 21.30; HRMS calcd. for $C_{22}H_{26}N_5O_3$ $[M+H]^+$ 408.203, found 408.2061, calcd. for $C_{22}H_{24}N_5O_3$ $[M-H]^-$ 406.1885, found 406.1942.

Synthetic procedures for 8a-8o

General synthetic procedures for 2-alkyl-5-aryl-4-(4-arylpiperazine-1-carbonyl)-2H-1,2,3-triazole (**8a-8o**)

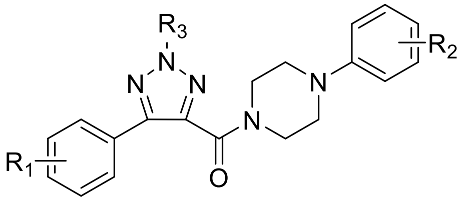

To a solution of 1 mmol of 5-aryl-4-(4-arylpiperazine-1-carbonyl)-2*H*-1,2,3-triazole (**7**) in acetone, K_2CO_3 (276 mg, 2 mmol) and 1.1 mmol of alkylating agents was added. The solution stirred at room temperature for 2-4 h. After concentrated, the residual was purified by Preparative-TLC (PE:EA = 8:1~4:1).

2-methyl-5-phenyl-4-(4-phenylpiperazine-1-carbonyl)-2H-1,2,3-triazole (**8a**). Yellow solid, yield: 81 %; ^{1}H NMR (400 MHz, $CDCl_3$) δ 7.74 (2H, dd, *J* = 8.10 Hz, 1.38 Hz), 7.40 (3H, m), 7.27 (2H, t, *J* = 7.94 Hz), 6.92 (3H, m), 6.02 (2H, d, *J* = 1.88 Hz), 4.26 (3H, s), 4.00 (2H, s), 3.48 (2H, s), 3.25 (2H, t, *J* = 5.07 Hz), 2.93 (2H, t, *J* = 4.91 Hz). ^{13}C NMR (150 MHz, $CDCl_3$) δ 162.41, 150.75, 145.78, 138.57, 129.39, 129.23 (2C), 128.99, 128.86 (2C), 127.28 (2C), 120.71, 116.79 (2C), 49.62, 49.44, 46.85, 42.02, 42.00; HRMS calcd. for $C_{20}H_{22}N_5O$ $[M+H]^+$ 348.1819, found 348.1894.

2-methyl-5-phenyl-4-(4-(3-chlorophenyl)piperazine-1-carbonyl)-2H-1,2,3-triazole (**8b**). Yellow solid, yield: 79 %; ^{1}H NMR (400 MHz, $CDCl_3$) δ 7.73 (dd, 2H, *J* = 8.10 Hz, 1.27 Hz), 7.40 (3H, m), 7.16 (1H, t, *J* = 8.08 Hz), 6.83 (2H, m), 6.73 (1H, dd, *J* = 7.99 Hz, 2.20 Hz), 4.26 (3H, s), 3.96 (2H, t, 5.16 Hz), 3.45 (2H, t, 5.09 Hz), 3.24 (2H, t, *J* = 5.19 Hz), 2.92 (2H, t, *J* = 5.09 Hz). ^{13}C NMR (150 MHz, $CDCl_3$) δ 162.39, 151.82, 145.89, 138.46, 135.03, 130.15, 129.39, 129.03, 128.87 (2C), 127.32 (2C), 120.28, 116.50, 114.55, 49.09, 48.89, 46.65, 42.03, 41.85; HRMS calcd. for $C_{20}H_{21}N_5OCl$ $[M+H]^+$ 382.1429, found 382.1445.

2-ethyl-5-phenyl-4-(4-(3-chlorophenyl)piperazine-1-carbonyl)-2H-1,2,3-triazole (**8c**). Yellow solid, yield: 77 %; ^{1}H NMR (400 MHz, $CDCl_3$) δ 7.75 (2H, d, *J* = 7.11 Hz), 7.40 (m, 3H), 7.17 (1H, t, *J* = 8.05 Hz), 6.85 (2H, m), 6.77 (2H,d, *J* = 8.55 Hz), 4.53 (3H, q, *J* = 7.34 Hz), 3.98 (2H,

t, *J* = 5.09 Hz), 3.49 (2H, t, *J* = 5.03 Hz), 3.26 (2H, t, *J* = 5.16 Hz), 2.94 (2H, t, *J* = 5.03 Hz), 1.64 (3H, t, *J* = 7.33 Hz). ^{13}C NMR (150 MHz, $CDCl_3$) δ 162.58, 151.66, 145.66, 138.07, 135.04, 130.17, 129.54, 128.95, 128.84 (2C), 127.36 (2C), 120.44, 116.56, 114.63, 50.86, 49.19, 48.98, 46.62, 41.80, 14.77; HRMS calcd. for $C_{21}H_{23}N_5OCl$ $[M+H]^+$ 396.1586, found 396.1613.

2-allyl-5-phenyl-4-(4-(3-chlorophenyl)piperazine-1-carbonyl)-2H-1,2,3-triazole (**8d**). Yellow solid, yield: 74 %; ^{1}H NMR (400 MHz, $CDCl_3$) δ 7.76 (2H, dd, *J* = 1.25 Hz, 9.02 Hz), 7.41 (3H, m), 7.18 (1H, t, *J* = 8.02 Hz), 6.88 (2H, m), 6.80 (2H, d, *J* = 8.52 Hz), 5.09 (2H, d, *J* = 6.18 Hz), 3.99 (2H, t, 5.18 Hz), 3.50 (2H, t, 5.14 Hz), 3.26 (2H, t, *J* = 5.18 Hz), 2.95 (2H, t, *J* = 5.01 Hz). ^{13}C NMR (150 MHz, $CDCl_3$) δ 162.37, 151.81, 146.02, 138.54, 135.02, 130.98, 130.14, 129.36, 129.04, 128.84 (2C), 127.39 (2C), 120.26, 120.05, 116.47, 114.53, 57.75, 49.08, 48.85, 46.67, 41.84; HRMS calcd. for $C_{22}H_{23}N_5OCl$ $[M+H]^+$ 408.1586, found 408.1602.

2-isopropyl-5-phenyl-4-(4-(3-chlorophenyl)piperazine-1-carbonyl)-2H-1,2,3-triazole (**8e**). Yellow solid, yield: 85 %; ^{1}H NMR (400 MHz, $CDCl_3$) δ 7.76 (2H, dd, *J* = 8.21 Hz, 1.20 Hz), 7.40 (3H, m), 7.16 (1H, t, *J* = 8.08 Hz), 6.84 (2H, m), 6.74 (1H, dd, *J* = 1.95 Hz, 8.43 Hz), 4.88 (1H, hept, *J* = 6.68 Hz), 3.97 (2H, t, *J* = 5.15 Hz), 3.48 (2H, t, *J* = 5.00 Hz), 3.25 (2H, t, *J* = 5.13 Hz), 2.95 (2H, t, *J* = 5.06 Hz), 1.65 (6H, d, *J* = 6.67 Hz). ^{13}C NMR (150 MHz, $CDCl_3$) δ 162.75, 151.75, 145.30, 137.62, 135.02, 130.14, 129.72, 128.84, 128.78 (2C), 127.37 (2C), 120.29, 116.48, 114.54, 65.29, 49.13, 48.88, 46.66, 41.82, 22.34 (2C); HRMS calcd. for $C_{22}H_{25}N_5OCl$ $[M+H]^+$ 410.1742, found 410.1764.

2-butyl-5-phenyl-4-(4-(3,5-chlorophenyl)piperazine-1-carbonyl)-2H-1,2,3-triazole (**8f**). Yellow solid, yield: 73 %; ^{1}H NMR (400 MHz, $CDCl_3$) δ 7.76 (dd, 2H, *J* = 8.15 Hz, 1.17 Hz), 7.40 (3H, m), 7.16 (1H, t, *J* = 8.08 Hz), 6.84 (2H, m), 6.74 (1H, dd, *J* = 8.25 Hz, 1.89 Hz), 4.47 (2H, t, *J* = 7.20 Hz), 3.97 (2H, t, 5.11 Hz), 3.47 (2H, t, 4.92 Hz), 3.25 (2H, t, *J* = 5.17 Hz), 2.95 (2H, t, *J* = 5.08 Hz), 2.01 (2H, quint, *J* = 7.38 Hz), 1.41 (2H, sext, *J* = 7.46 Hz), 0.98 (3H, t, *J* = 7.39 Hz). ^{13}C NMR (150 MHz, $CDCl_3$) δ 162.57, 151.69, 145.65, 137.97, 135.03, 130.15, 129.53, 128.92, 128.81 (2C), 127.35 (2C), 120.38, 116.53, 114.59, 55.13, 49.18, 48.94, 46.64, 41.81, 31.60, 19.73, 13.48; HRMS calcd. for $C_{23}H_{27}N_5OCl$ $[M+H]^+$ 424.1899, found 424.1931.

2-methyl-5-phenyl-4-(4-(3,5-dimethylphenyl)piperazine-1-carbonyl)-2H-1,2,3-triazole (**8g**). Yellow solid, yield: 74 %; ^{1}H NMR (600 MHz, $CDCl_3$) δ 7.77 (2H, dd, J = 8.52 Hz, 1.38 Hz), 7.42 (2H, m), 7.37 (1H, m), 6.57 (1H, s), 6.51 (2H, s), 4.25 (3H, s), 3.97 (2H, t, J = 5.12 Hz), 3.45 (2H, t, J = 5.04 Hz), 3.22 (2H, t, J = 5.11 Hz), 2.91 (2H, t, J = 5.03 Hz), 2.27 (6H, s). ^{13}C NMR (150 MHz, $CDCl_3$) δ 162.16, 150.71, 145.46, 138.50 (2C), 138.40, 129.23, 128.72, 128.62 (2C), 127.03 (2C), 122.30, 114.49 (2C), 49.47, 49.31, 46.69, 41.86, 41.74, 21.36 (2C); HRMS calcd. for $C_{22}H_{26}N_5O$ $[M+H]^+$ 376.2132, found 376.217.

2-methyl-5-phenyl-4-(4-(3,5-dimethoxyphenyl)piperazine-1-carbonyl)-2H-1,2,3-triazole (**8h**). Yellow solid, yield: 81 %; ^{1}H NMR (600 MHz, $CDCl_3$) δ 7.73(2H, dd, J = 8.51 Hz, 1.43 Hz), 7.40 (2H, m), 7.36 (1H, m), 6.04 (1H, t, J = 2.02 Hz), 6.01 (2H, d, J = 2.01 Hz), 4.25 (3H, s), 3.94 (2H, t, J = 5.16 Hz), 3.75 (6H, s), 3.43 (2H, t, J = 5.05 Hz), 3.22 (2H, t, J = 5.16 Hz), 2.89 (2H, t, J = 5.09 Hz). ^{13}C NMR (150 MHz, $CDCl_3$) δ 162.34, 161.44 (2C), 152.68, 145.72, 138.49, 128.94, 128.81 (2C), 128.49, 127.22 (2C), 95.61 (2C), 92.24, 55.19 (2C), 49.38, 49.25, 46.70, 41.94, 41.90; HRMS calcd. for $C_{22}H_{26}N_5O_3$ $[M+H]^+$ 408.203, found 408.2144.

2-methyl-5-(2-fluorophenyl)-4-(4-(3,5-dimethylphenyl)piperazine-1-carbonyl)-2H-1,2,3-triazole (**8i**). Yellow solid, yield: 80 %; ^{1}H NMR (600 MHz, $CDCl_3$) δ 7.69 (td, 1H, J = 7.56 Hz, 1.68 Hz), 7.37 (1H, m), 7.22 (1H, td, J = 7.57 Hz, 0.90 Hz), 7.10 (1H, t, J = 9.06 Hz), 6.58 (1H, s), 6.56 (2H, s), 4.28 (3H, s), 3.93 (2H, s), 3.67 (2H, s), 3.23 (2H, t, J = 4.90 Hz), 3.04 (2H, t, J = 4.60 Hz), 2.28 (6H, s). ^{13}C NMR (150 MHz, $CDCl_3$) δ 161.73, 159.72 (d, J = 247.85 Hz), 151.04, 141.87, 140.33, 138.82 (2C), 130.79 (d, J = 8.30 Hz), 130.29 (d, J = 2.52 Hz), 124.49 (d, J = 3.39 Hz), 122.52, 117.65(d, J = 13.26 Hz), 115.97 (d, J = 21.37 Hz), 114.73 (2C), 49.71, 49.59, 46.94, 42.06, 41.96, 21.56 (2C); HRMS calcd. for $C_{22}H_{25}N_5OF$ $[M+H]^+$ 394.2038, found 394.2053.

2-ethyl-5-(2-fluorophenyl)-4-(4-(3,5-dimethylphenyl)piperazine-1-carbonyl)-2H-1,2,3-triazole (**8j**). Yellow solid, yield: 78 %; ^{1}H NMR (600 MHz, $CDCl_3$) δ 7.70 (1H, td, J = 7.54 Hz, 1.69 Hz), 7.36 (1H, m), 7.22 (1H, td, J = 7.54 Hz, 1.01 Hz), 7.12 (t, 1H, J = 9.41 Hz), 6.57 (s, 1H), 6.55 (s, 2H), 4.55 (q, 2H), 3.93 (2H, t, J =4.88 Hz), 3.68 (2H, t, J =5.90 Hz), 3.23 (2H, t, J = 5.02 Hz), 3.05 (2H, t, J = 4.59 Hz), 2.63 (3H, s), 2.28 (6H, s). ^{13}C NMR (150 MHz, $CDCl_3$) δ 161.91, 159.74 (d, J = 247.89 Hz), 151.09, 141.60, 140.05, 138.80 (2C), 130.69 (d, J = 8.20 Hz), 130.34 (d, J = 2.31 Hz), 124.46 (d, J = 3.24 Hz), 122.42, 117.85 (d, J = 13.86 Hz), 115.96 (d, J = 21.57 Hz),

114.67 (2C), 50.52, 49.70, 49.53, 47.00, 41.99, 21.58 (2C), 14.82; HRMS calcd. for $C_{23}H_{27}N_5OF$ $[M+H]^+$ 408.2194, found 408.2236.

2-methyl-5-(2-fluorophenyl)-4-(4-(3,5-dimethoxyphenyl)piperazine-1-carbonyl)-2H-1,2,3-triazole (**8k**). Yellow solid, yield: 77 %; ^{1}H NMR (600 MHz, $CDCl_3$) δ 7.67 (1H, td, *J* = 8.02 Hz, 1.52 Hz), 7.35 (1H, m), 7.20 (1H, t, *J* = 7.49 Hz), 7.10 (1H, t, *J* = 9.36 Hz), 6.05 (2H, m), 6.03 (1H, m), 4.25 (3H, s), 3.88 (2H, t, *J* = 4.95 Hz), 3.74 (6H, s), 3.64 (2H, t, *J* = 4.92 Hz), 3.22 (2H, t, *J* = 4.99 Hz), 3.02 (2H, t, *J* = 4.81 Hz). ^{13}C NMR (150 MHz, $CDCl_3$) δ 161.64, 161.36 (2C), 159.59 (d, *J* = 247.81 Hz), 152.77, 141.74, 140.13, 130.71 (d, *J* = 8.28 Hz), 130.16 (d, *J* = 2.78 Hz), 124.39 (d, *J* = 3.36 Hz), 117.52 (d, *J* = 13.59 Hz), 115.85 (d, *J* = 21.57 Hz), 95.48 (2C), 92.08, 55.11 (2C), 49.29, 49.14, 46.72, 41.94, 41.76; HRMS calcd. for $C_{22}H_{25}N_5O_3F$ $[M+H]^+$ 426.1936, found 426.1977.

2-methyl-5-(4-fluorophenyl)-4-(4-(3,5-dimethylphenyl)piperazine-1-carbonyl)-2H-1,2,3-triazole (**8l**). Yellow solid, yield: 79 %; ^{1}H NMR (600 MHz, $CDCl_3$) δ 7.77 (2H, m), 7.11 (2H, m), 6.57 (1H, s), 6.52 (2H, s), 4.25 (3H, s), 3.96 (2H, t, *J* = 5.18 Hz), 3.51 (2H, t, *J* = 5.10 Hz), 3.23 (2H, t, *J* = 5.17 Hz), 2.98 (2H, t, *J* = 5.10 Hz), 2.27 (6H, s). ^{13}C NMR (150 MHz, $CDCl_3$) δ 163.00 (d, *J* = 247.59 Hz), 162.11, 150.84, 145.09, 138.73 (2C), 138.26, 129.18 (2C, d, *J* = 8.22 Hz), 125.60(d, *J* = 3.41 Hz), 122.56, 117.51, 115.76(2C, d, *J* = 21.75 Hz), 114.69 (2C), 110.71, 49.84, 49.58, 46.94, 42.09, 41.90, 21.48 (2C); HRMS calcd. for $C_{22}H_{25}N_5OF$ $[M+H]^+$ 394.2038, found 394.2065.

2-methyl-5-(4-fluorophenyl)-4-(4-(3,5-dimethoxyphenyl)piperazine-1-carbonyl)-2H-1,2,3-triazole (**8m**). Yellow solid, yield: 74 %; ^{1}H NMR (600 MHz, $CDCl_3$) δ 7.78 (1H, m), 7.63 (1H, t, *J* = 4.01 Hz), 7.32 (2H, d, *J* = 4.72 Hz), 6.03 (3H, s), 4.24 (3H, s), 3.95 (2H, t, *J* = 4.93 Hz), 3.74 (6H, s), 3.47 (2H, t, *J* = 4.75 Hz), 3.24 (2H, t, *J* = 5.00 Hz), 2.97 (2H, t, *J* = 4.83 Hz). ^{13}C NMR (150 MHz, $CDCl_3$) δ 163.02 (d, *J* = 247.50 Hz), 162.12, 161.43 (2C), 152.63, 145.17, 138.16, 129.20 (2C, d, *J* = 8.25 Hz), 125.56 (d, *J* = 2.67 Hz), 115.77(2C, d, *J* = 21.53 Hz), 95.61 (2C), 92.25, 55.17 (2C), 49.57, 49.29, 46.76, 41.95, 41.92; HRMS calcd. for $C_{22}H_{25}N_5O_3F$ $[M+H]^+$ 426.1936, found 426.2035.

2-methyl-5-(3-chlorophenyl)-4-(4-(3,5-dimethylphenyl)piperazine-1-carbonyl)-2H-1,2,3-triazole (**8n**). Yellow solid, yield: 81 %; ^{1}H NMR (600 MHz, $CDCl_3$) δ 7.81 (m, 1H), 7.66 (1H, m), 7.34

(2H, m), 6.57 (1H, s), 6.53 (2H, s), 4.26 (3H, s), 3.97 (2H, t, J = 5.14 Hz), 3.49 (2H, t, J = 5.10 Hz), 3.24 (2H, t, J = 5.17 Hz), 2.97 (2H, t, J = 5.10 Hz), 2.27 (6H, s). ^{13}C NMR (150 MHz, $CDCl_3$) δ 161.91, 150.82, 144.41, 138.70 (2C), 138.68, 134.65, 131.09, 130.01, 128.85, 127.21, 125.28, 122.56, 114.71 (2C), 49.82, 49.50, 46.91, 42.08, 41.97, 21.47 (2C); HRMS calcd. for $C_{22}H_{25}N_5OCl$ $[M+H]^+$ 410.1742, found 410.1943.

2-methyl-5-(3-chlorophenyl)-4-(4-(3,5-dimethoxyphenyl)piperazine-1-carbonyl)-2H-1,2,3-triazole (**8o**). Yellow solid, yield: 83 %; ^{1}H NMR (600 MHz, $CDCl_3$) δ 7.74 (2H, m), 7.09 (2H, m), 6.03 (3H, s), 4.24 (3H, s), 3.94 (2H, t, J = 4.93 Hz), 3.75 (6H, s), 3.50 (2H, t, J = 4.75 Hz), 3.23 (2H, t, J = 5.00 Hz), 2.98 (2H, t, J = 4.83 Hz). ^{13}C NMR (150 MHz, $CDCl_3$) δ 161.92, 161.42 (2C), 152.64, 144.49, 138.60, 134.67, 131.08, 130.03, 128.90, 127.24, 125.32, 95.63 (2C), 92.31, 55.17 (2C), 49.55, 49.26, 46.74, 42.00, 41.95; HRMS calcd. for $C_{22}H_{25}N_5O_3Cl$ $[M+H]^+$ 442.164, found 442.166.

An unambiguous synthetic route for 8g, 8h and 8i

Synthetic procedures for 4,5-dibromo-2H-1,2,3-triazole

To a suspended of 1,3-dibromo-5,5-dimethylimidazolidine-2,4-dione (DBDMH, 2860 mg, 10 mmol) in water (40 mL), 1,2,3-triazole (691 mg, 10 mmol) was added dropwise at 10 °C. The mixture was stirred 60 min at same temperature. After that, filtered and wash by water (40 mL).

Synthetic procedures for 4,5-dibromo-2-methyl-2H-1,2,3-triazole (**13**)

To a solution of 4,5-dibromo-2*H*-1,2,3-triazole (2269 mg, 10 mmol) in acetone (40 mL), K_2CO_3 (2764 mg, 20 mmol) and dimethyl sulfate (1387 mg, 11 mmol) was added. The solution stirred at room temperature for 2 h. After concentrated, the residual was purified by column chromatography (PE:EA = 12:1). ^{1}H NMR (600 MHz, $CDCl_3$) δ 4.17 (s, 3H).

Synthetic procedures for 5-bromo-2-methyl-2H-1,2,3-triazole-4-carbaldehyde (**14**)

To a solution of 4,5-dibromo-2-methyl-2*H*-1,2,3-triazole (**13**, 2410 mg, 10 mmol) in THF (40 mL) under nitrogen, 5 mL of i-PrMgCl (2M in THF) was added dropwise at -45 °C. The mixture was stirred for 30 minutes at -45 °C. Then, DMF (1.54 mL, 20 mmol) was added dropwise to the reaction mixture at -45 °C. After stirring for another 15 minutes at -45 °C, 18% HCl (10 mL) was added. The solution was extract by ethyl acetate, organic layers were washed with brine, dried over anhydrous Na_2SO_4, filtered and concentrated under reduced pressure. ^{1}H NMR (600 MHz, $CDCl_3$) δ 10.03 (s, 1H), 4.27 (s, 3H). MS (ESI) m/z 190.0 $[M+H]^+$.

General synthetic procedures for 5-aryl-2-methyl-2H-1,2,3-triazole-4-carbaldehyde (**15**)

5-bromo-2-methyl-2*H*-1,2,3-triazole-4-carbaldehyde (**14**, 190 mg, 1 mmol), 1.2 mmol of arylboronic acid, water/dioxane (4 mL/12 mL), K_2CO_3 (276 mg, 2 mmol) and $Pd(PPh_3)_4$ (58 mg, 0.05 mmol) was mixed under nitrogen. The reaction mixture was warmed to 100 °C and stirred for 3 h. The finally solution was filtered and concentrated under reduced pressure.

5-Phenyl-2-methyl-2H-1,2,3-triazole-4-carbaldehyde (**15a**): White solid, ^{1}H NMR (600 MHz, $CDCl_3$) δ 10.17 (s, 1H), 8.01(s, 2H, J = 7.76, 1.62 Hz), 7.45 (m, 3H), 4.29 (s, 3H). MS (ESI) m/z 188.1 $[M+H]^+$, m/z 210.1 $[M+Na]^+$.

5-(2-fluorophenyl)-2-methyl-2H-1,2,3-triazole-4-carbaldehyde (**15b**): White solid, ^{1}H NMR (600 MHz, $CDCl_3$) δ 10.08 (s, 1H), 7.56 (td, 1H, J = 7.44, 1.26 Hz), 7.41 (m, 1H), 7.21 (t, 1H, J = 7.52 Hz), 7.16 (t, 1H, J = 8.23 Hz), 4.28 (s, 3H). MS (ESI) m/z 206.1 $[M+H]^+$, m/z 228.1 $[M+Na]^+$.

General synthetic procedures for 5-aryl-2-methyl-2H-1,2,3-triazole-4-carboxylic acid (**16**)

Aqueous hydrogen peroxide (30%, 8.0 mL, 80 mmol) was added dropwise to a stirred solution of 50% aq. KOH (2.24 g KOH + 2.24 mL water, 40 mmol) and 10 mmol of 5-aryl-2-methyl-2*H*-1,2,3-triazole-4-carbaldehyde (**15a, 15b**) in methanol (25 mL) at 65 °C for 20 min. The mixture was then stirred at the same temperature for 10 min, cooled, acidified with 18% HCl to give 5-aryl-2-methyl-2*H*-1,2,3-triazole-4-carboxylic acid.

5-Phenyl-2-methyl-2H-1,2,3-triazole-4-carbaldehyde (**16a**): White solid, ^{1}H NMR (600 MHz, $CDCl_3$) δ 10.17 (s, 1H), 8.01(s, 2H, J = 7.76, 1.62 Hz), 7.45 (m, 3H), 4.29 (s, 3H). MS (ESI) m/z 204.1 $[M+H]^+$.

5-(2-fluorophenyl)-2-methyl-2H-1,2,3-triazole-4-carboxylic acid (**16b**): White solid, ^{1}H NMR (600 MHz, $CDCl_3$) δ 10.08 (s, 1H), 7.56 (td, 1H, J = 7.44, 1.26 Hz), 7.41 (m, 1H), 7.21 (t, 1H, J = 7.52 Hz), 7.16 (t, 1H, J = 8.23 Hz), 4.28 (s, 3H). MS (ESI) m/z 222.1 $[M+H]^+$, m/z 244.0 $[M+Na]^+$.

General synthetic procedures for 2-alkyl-5-aryl-4-(4-arylpiperazine-1-carbonyl)-2H-1,2,3-triazole (**8**) *by an unambiguous synthetic route*

A solution of 1 mmol of 5-aryl-2-methyl-2*H*-1,2,3-triazole-4-carboxylic acid (**16a, 16b**), 1-Ethyl-3-(3-dimethylaminopropyl)carbodiimide hydrochloride (192 mg, 1 mmol), 1-hydroxybenzotriazole (135 mg, 1 mmol) and 1 mmol of arylpiperazine, was stirred at room temperature overnight. After concentrated, the residual was purified by Preparative-TLC (PE: EA = 8:1~4:1). **8g**, **8h** and **8i** was obtained, and all experimental property is identical as products obtain by formal route.

^{1}H NMR and ^{13}C NMR spectra of the products

^{1}H NMR of 5-phenyl-4-(4-phenylpiperazine-1-carbonyl)-2*H*-1,2,3-triazole (**7a**)

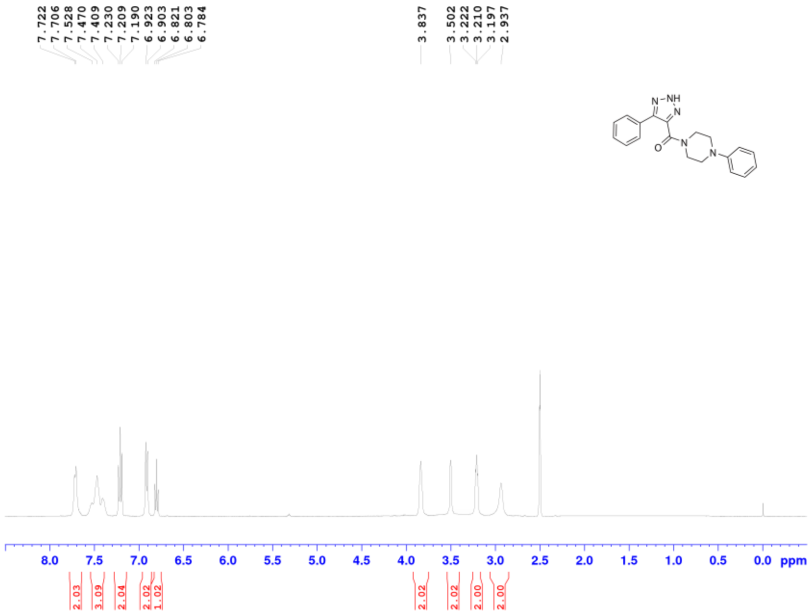

^{13}C NMR of 5-phenyl-4-(4-phenylpiperazine-1-carbonyl)-2*H*-1,2,3-triazole (**7a**)

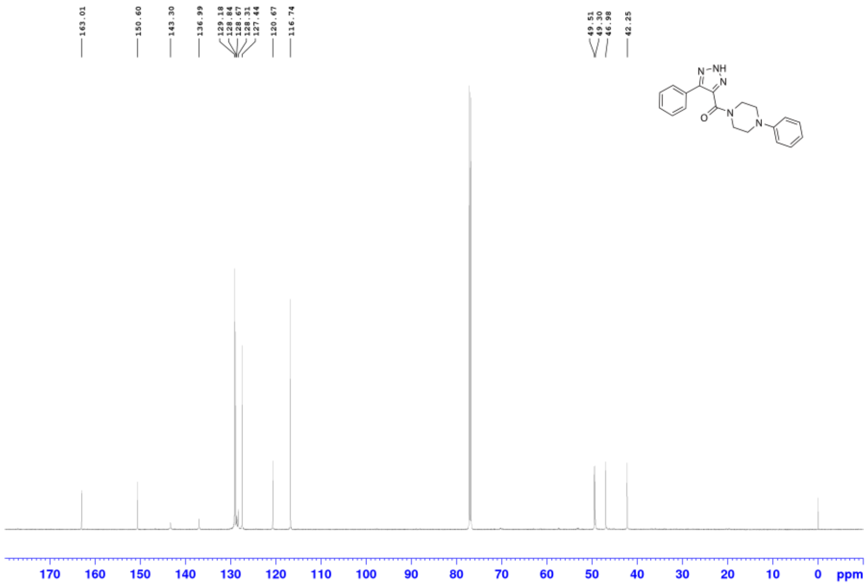

^{1}H NMR of 5-phenyl-4-(4-(3-chlorophenyl)piperazine-1-carbonyl)-2*H*-1,2,3-triazole (**7b**)

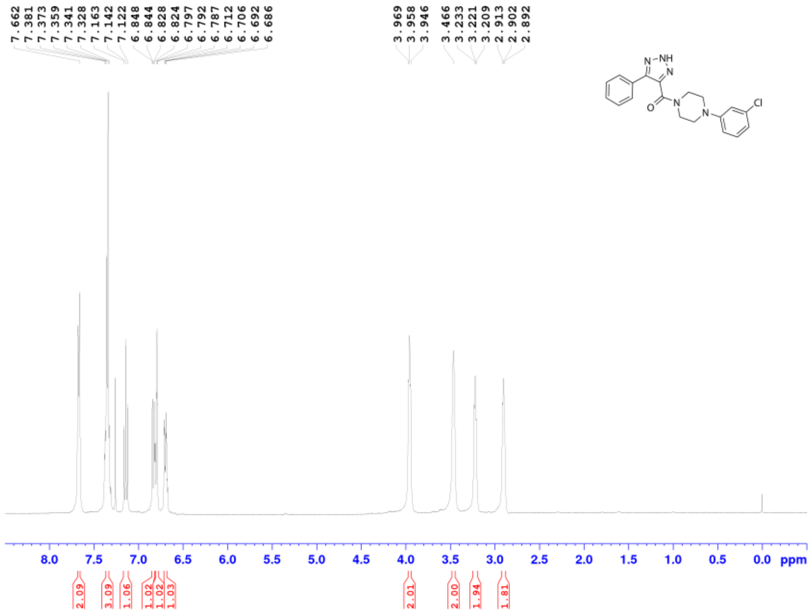

^{13}C NMR of 5-phenyl-4-(4-(3-chlorophenyl)piperazine-1-carbonyl)-2*H*-1,2,3-triazole (**7b**)

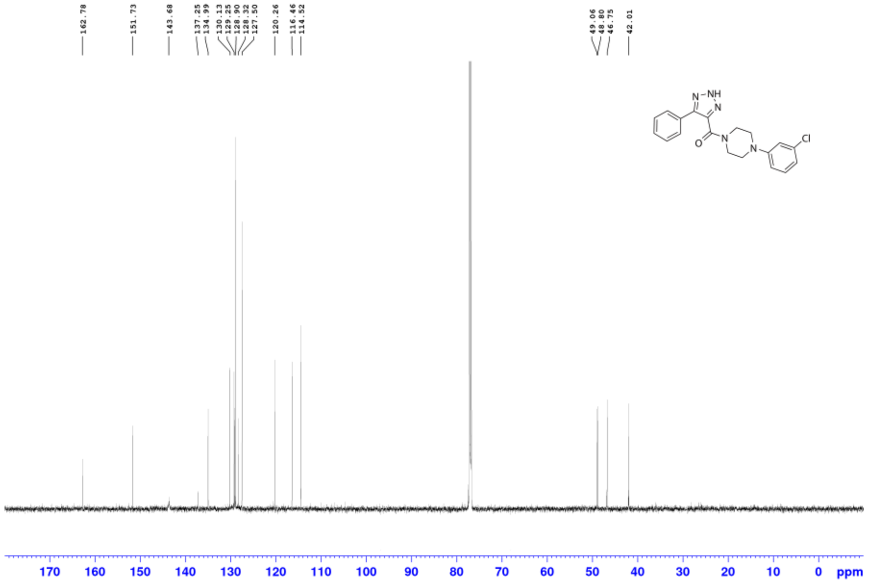

^{1}H NMR of 5-phenyl-4-(4-(4-methylphenyl)piperazine-1-carbonyl)-2*H*-1,2,3-triazole (**7c**)

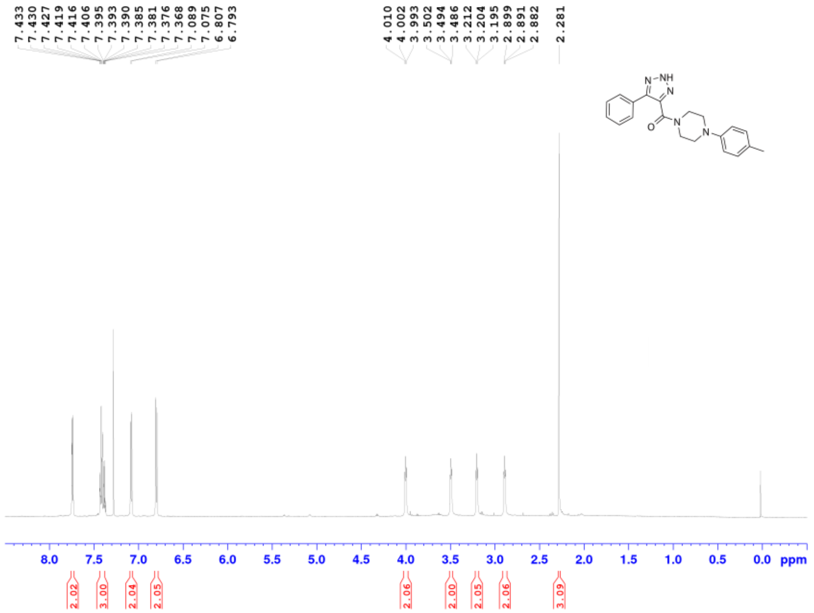

^{13}C NMR of 5-phenyl-4-(4-(4-methylphenyl)piperazine-1-carbonyl)-2*H*-1,2,3-triazole (**7c**)

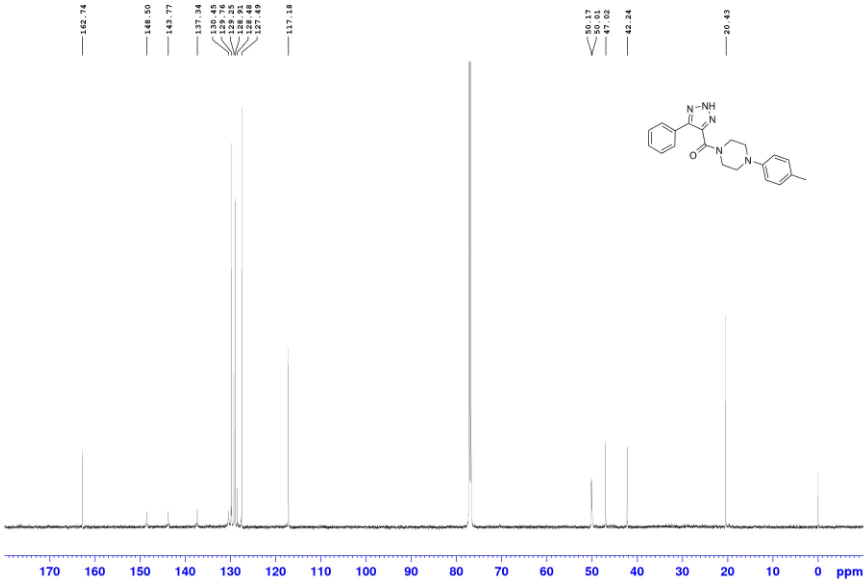

^{1}H NMR of 5-phenyl-4-(4-(3-methoxyphenyl)piperazine-1-carbonyl)-2*H*-1,2,3-triazole (**7d**)

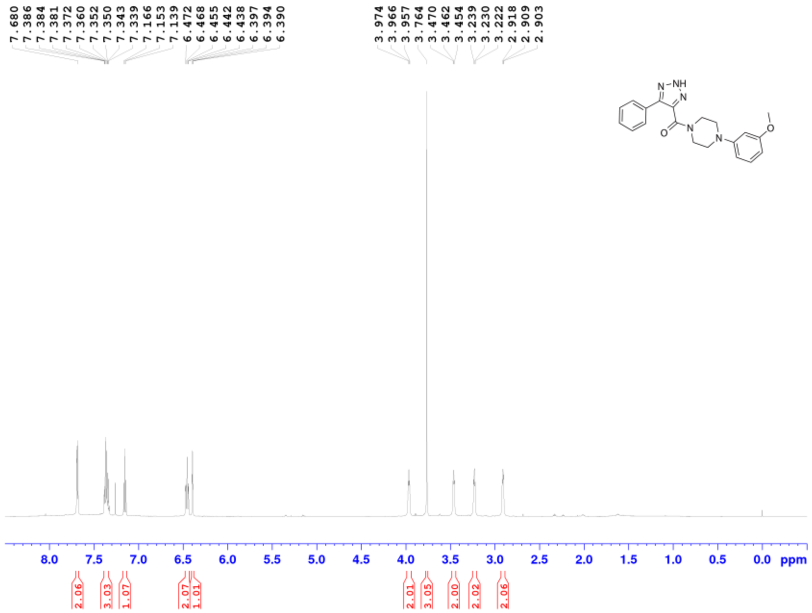

^{13}C NMR of 5-phenyl-4-(4-(3-methoxyphenyl)piperazine-1-carbonyl)-2*H*-1,2,3-triazole (**7d**)

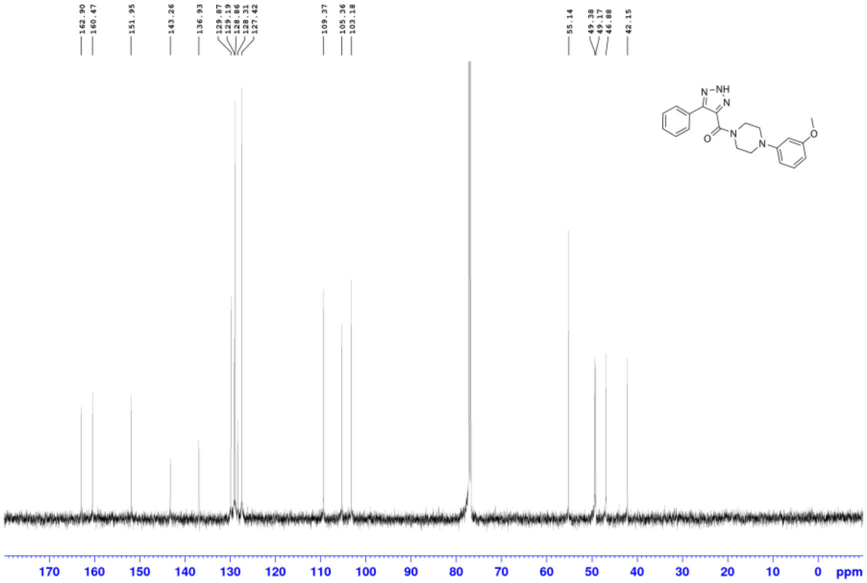

^{1}H NMR of 5-phenyl-4-(4-(2,4-dimethylphenyl)piperazine-1-carbonyl)-2*H*-1,2,3-triazole (**7e**)

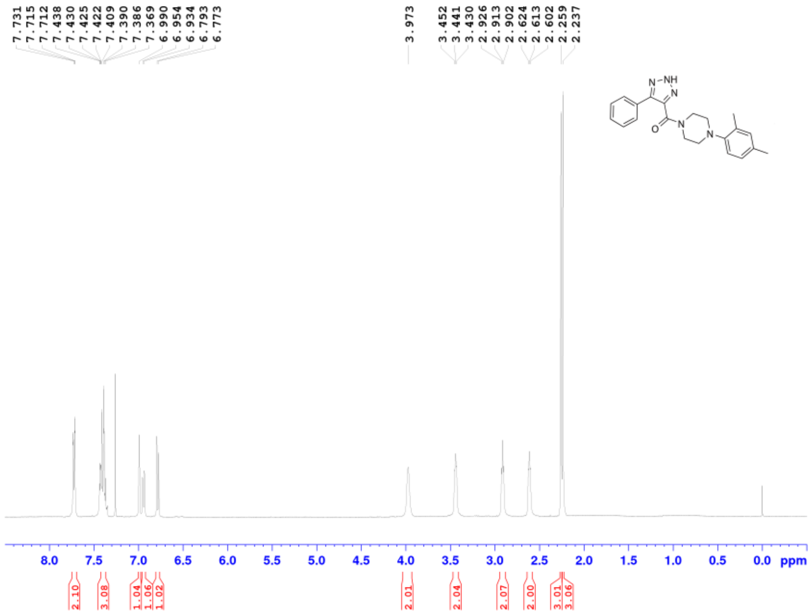

^{13}C NMR of 5-phenyl-4-(4-(2,4-dimethylphenyl)piperazine-1-carbonyl)-2*H*-1,2,3-triazole (**7e**)

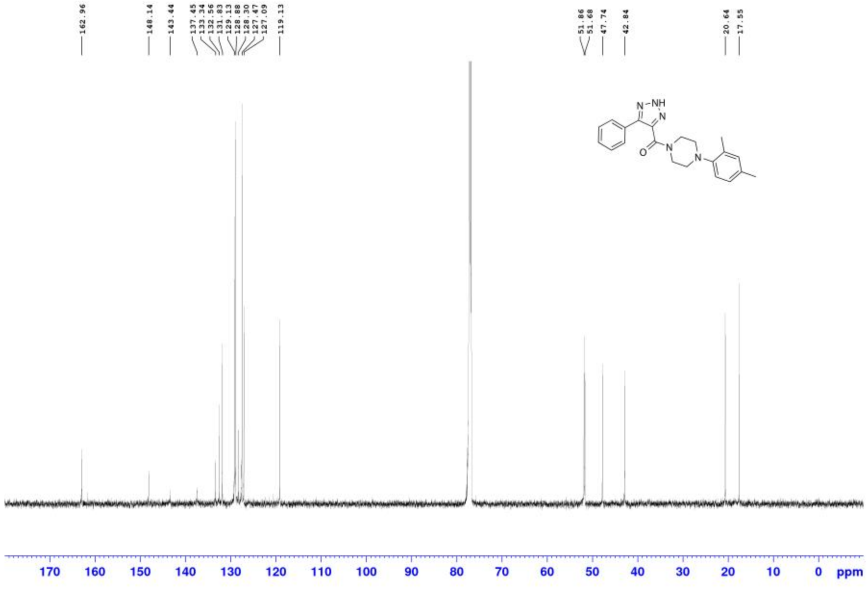

^{1}H NMR of 5-phenyl-4-(4-(2,5-dimethylphenyl)piperazine-1-carbonyl)-2*H*-1,2,3-triazole (**7f**)

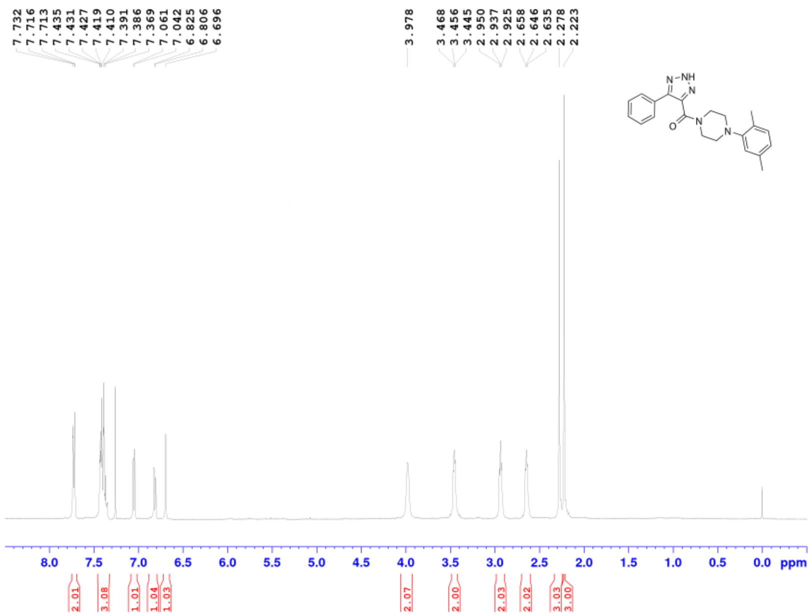

^{13}C NMR of 5-phenyl-4-(4-(2,5-dimethylphenyl)piperazine-1-carbonyl)-2*H*-1,2,3-triazole (**7f**)

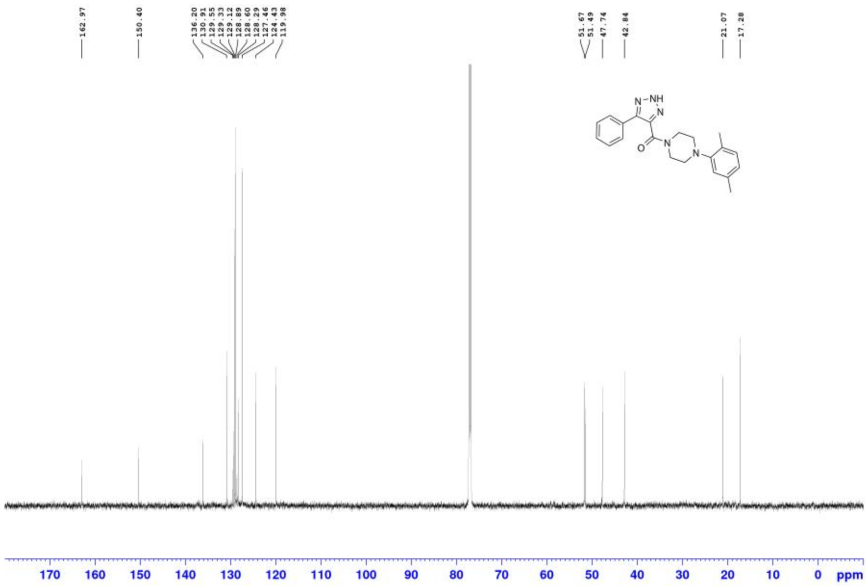

^{1}H NMR of 5-phenyl-4-(4-(3,4-dimethylphenyl)piperazine-1-carbonyl)-2*H*-1,2,3-triazole (**7g**)

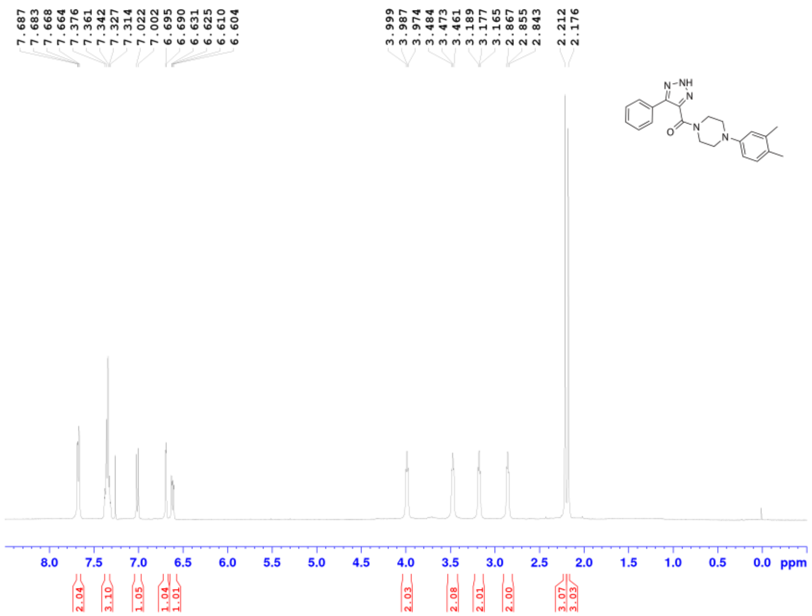

^{13}C NMR of 5-phenyl-4-(4-(3,4-dimethylphenyl)piperazine-1-carbonyl)-2*H*-1,2,3-triazole (**7g**)

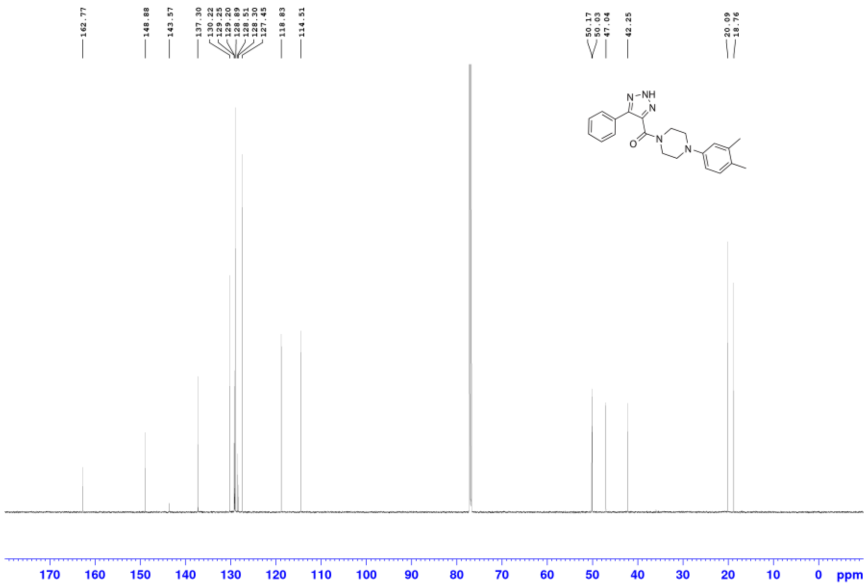

^{1}H NMR of 5-phenyl-4-(4-(3,5-dimethylphenyl)piperazine-1-carbonyl)-2*H*-1,2,3-triazole (**7h**)

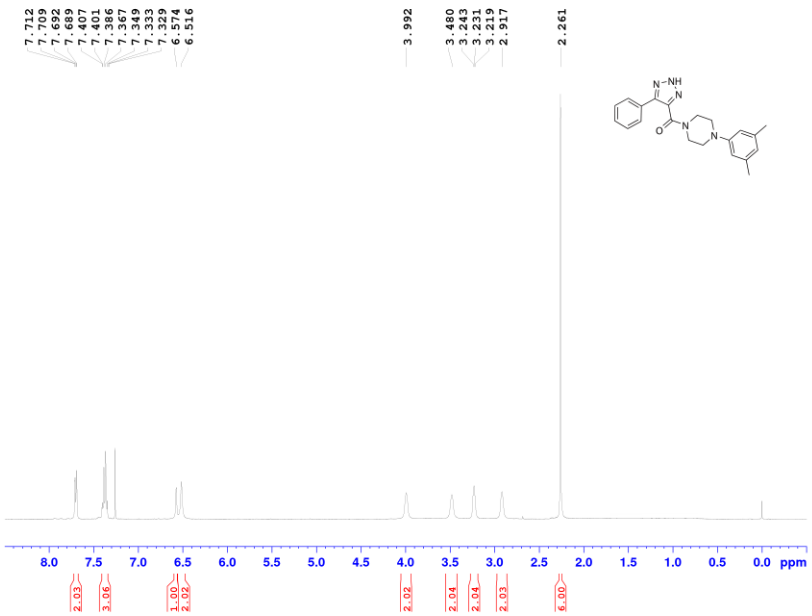

^{13}C NMR of 5-phenyl-4-(4-(3,5-dimethylphenyl)piperazine-1-carbonyl)-2*H*-1,2,3-triazole (**7h**)

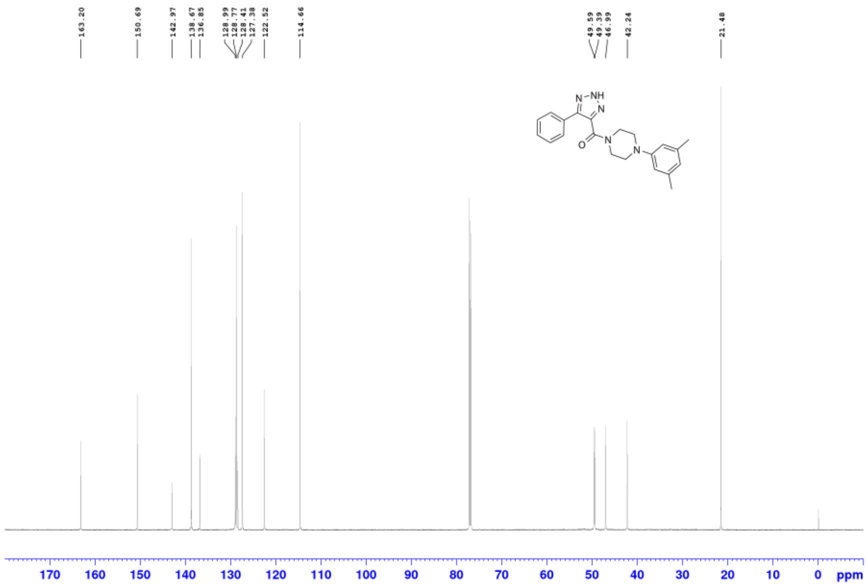

^{1}H NMR of 5-phenyl-4-(4-(2,5-dimethoxyphenyl)piperazine-1-carbonyl)-2*H*-1,2,3-triazole (**7i**)

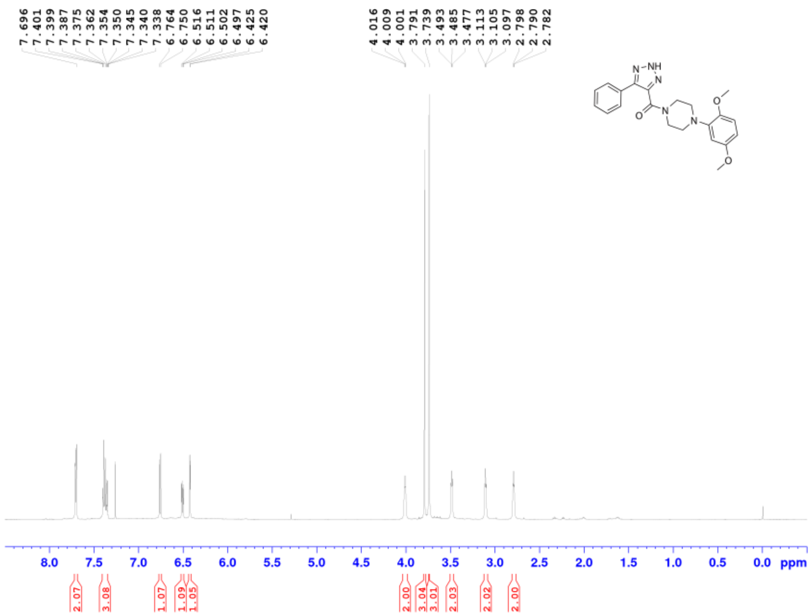

^{13}C NMR of 5-phenyl-4-(4-(2,5-dimethoxyphenyl)piperazine-1-carbonyl)-2*H*-1,2,3-triazole (**7i**)

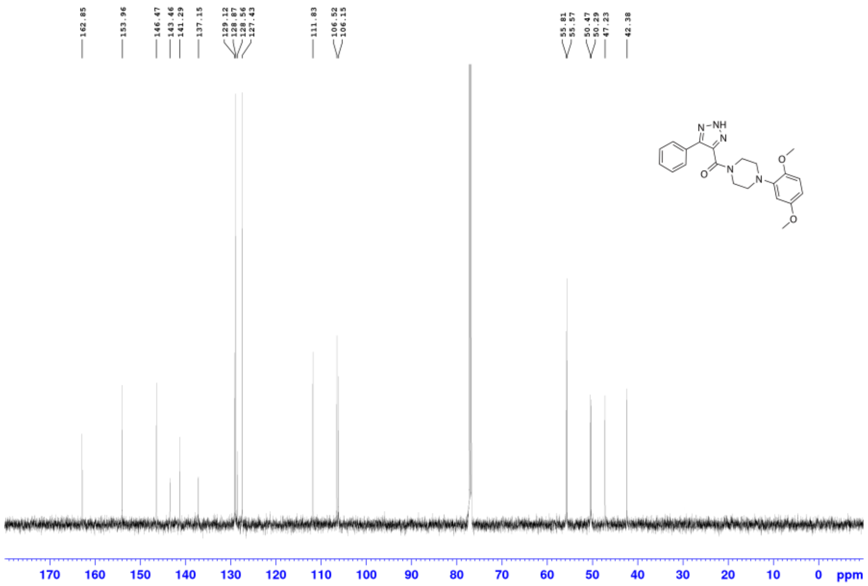

^{1}H NMR of 5-phenyl-4-(4-(3,5-dimethoxyphenyl)piperazine-1-carbonyl)-2*H*-1,2,3-triazole (**7j**)

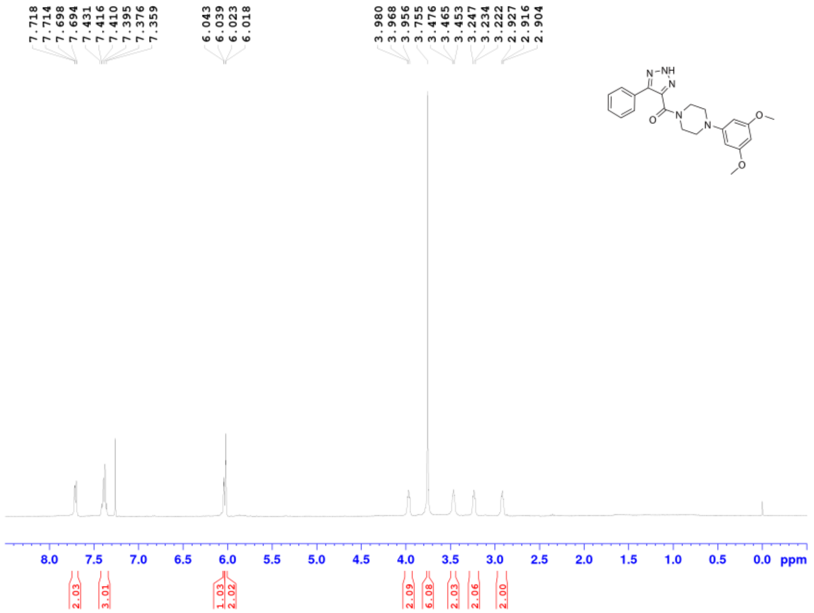

^{13}C NMR of 5-phenyl-4-(4-(3,5-dimethoxyphenyl)piperazine-1-carbonyl)-2*H*-1,2,3-triazole (**7j**)

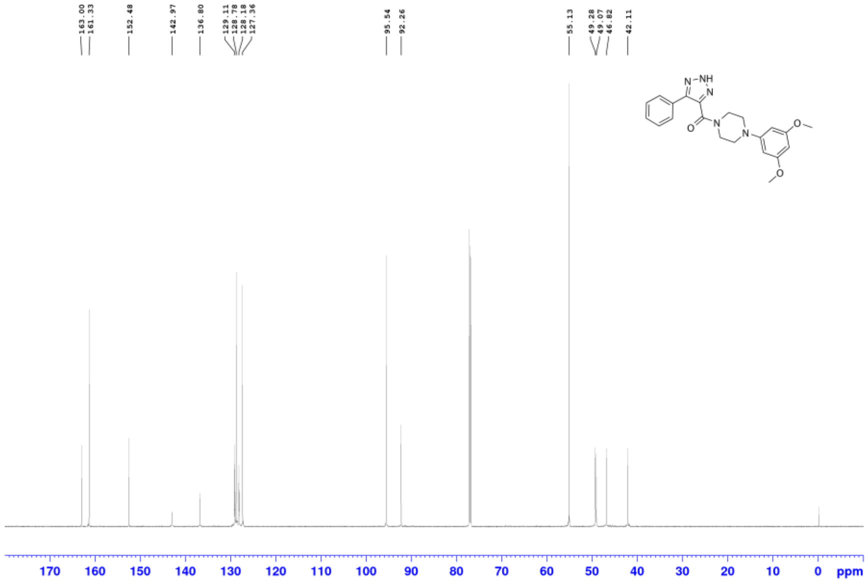

^{1}H NMR of 5-(2-fluorophenyl)-4-(4-(3,5-dimethylphenyl)piperazine-1-carbonyl)-2*H*-1,2,3-triazole (**7k**)

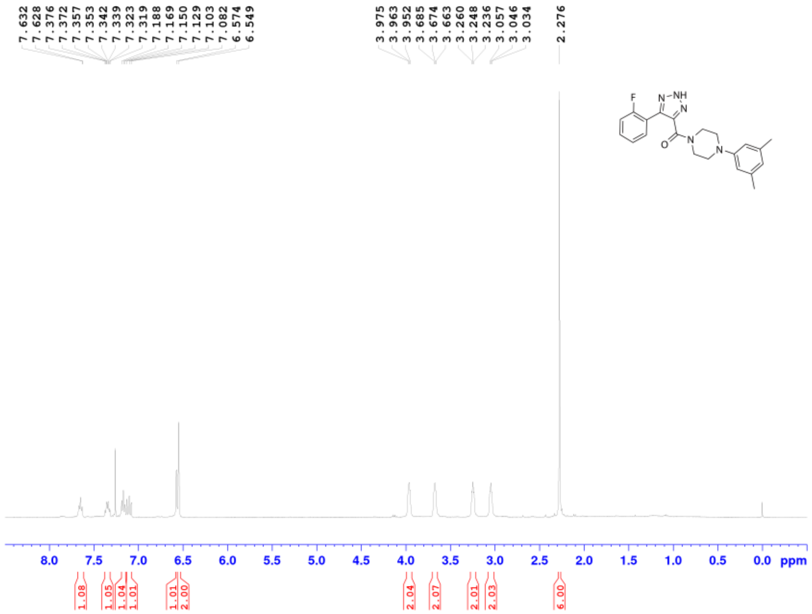

^{13}C NMR of 5-(2-fluorophenyl)-4-(4-(3,5-dimethylphenyl)piperazine-1-carbonyl)-2*H*-1,2,3-triazole (**7k**)

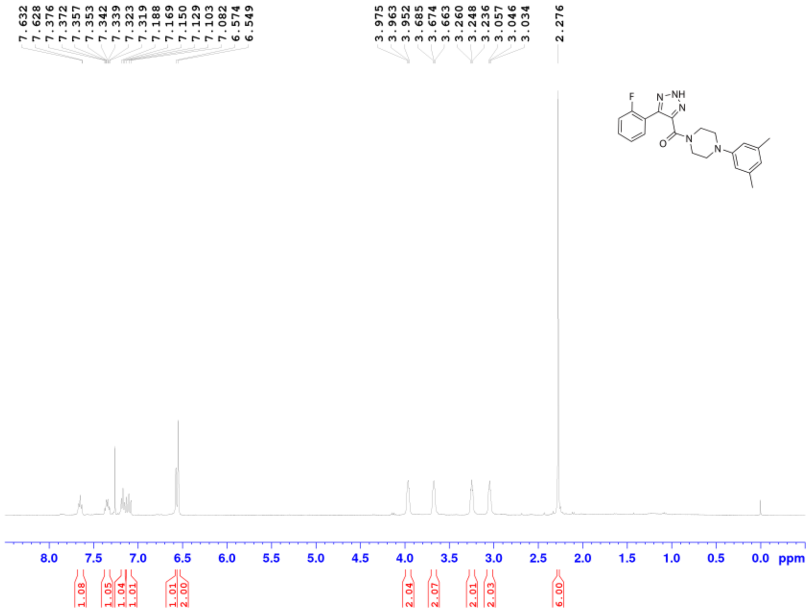

^{1}H NMR of 5-(2-fluorophenyl)-4-(4-(3,5-dimethoxyphenyl)piperazine-1-carbonyl)-2*H*-1,2,3-triazole (**7l**)

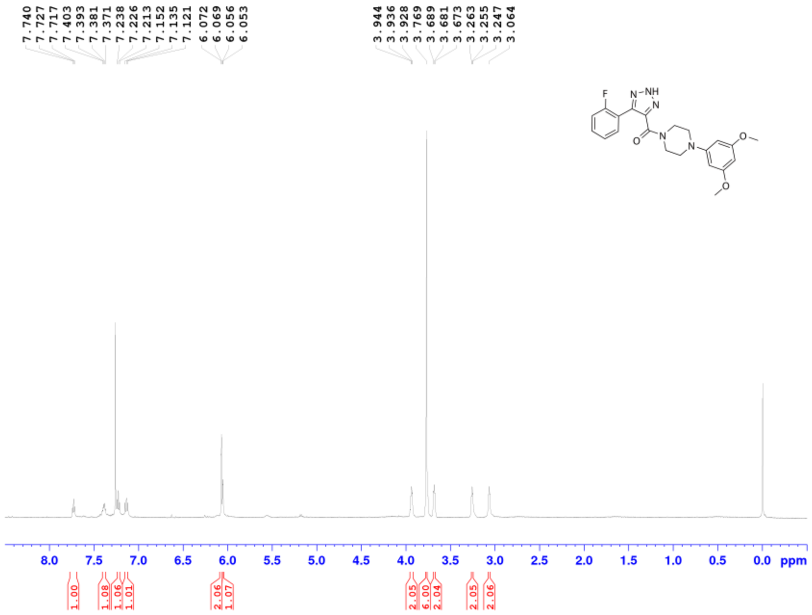

^{13}C NMR of 5-(2-fluorophenyl)-4-(4-(3,5-dimethoxyphenyl)piperazine-1-carbonyl)-2*H*-1,2,3-triazole (**7l**)

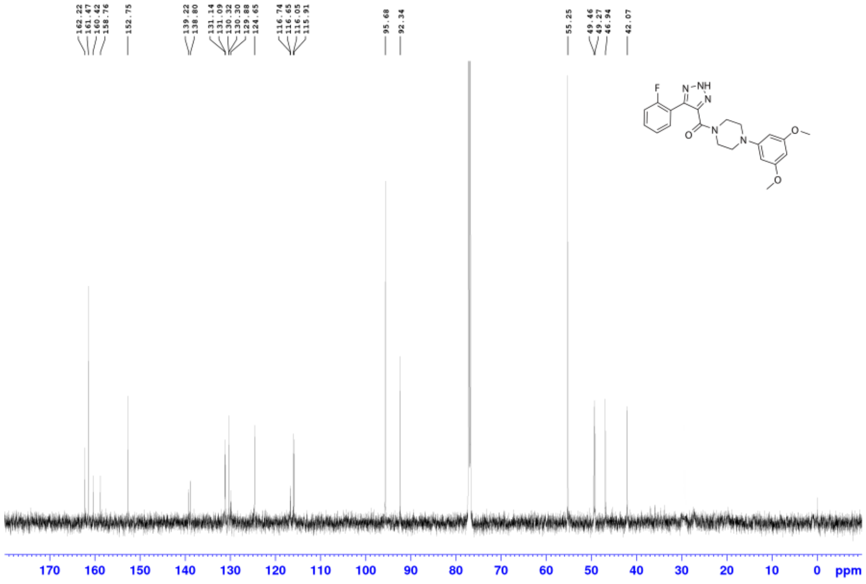

^{1}H NMR of 5-(3-fluorophenyl)-4-(4-(3,5-dimethoxyphenyl)piperazine-1-carbonyl)-2*H*-1,2,3-triazole (**7m**)

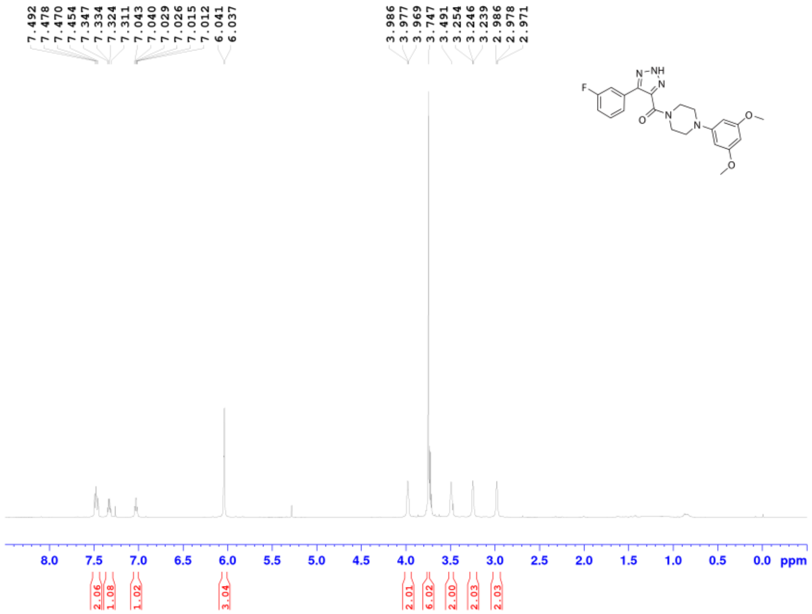

^{13}C NMR of 5-(3-fluorophenyl)-4-(4-(3,5-dimethoxyphenyl)piperazine-1-carbonyl)-2*H*-1,2,3-triazole (**7m**)

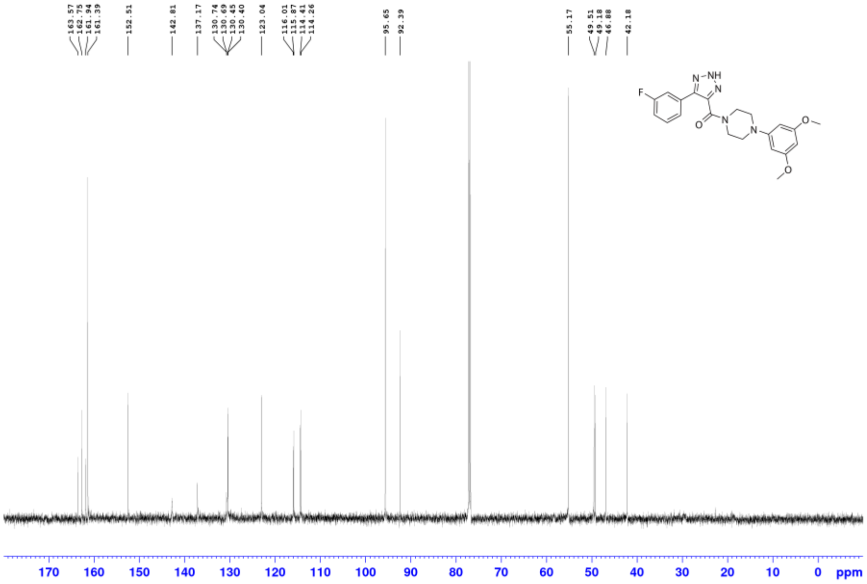

^{1}H NMR of 5-(4-fluorophenyl)-4-(4-(3,5-dimethylphenyl)piperazine-1-carbonyl)-2*H*-1,2,3-triazole (**7n**)

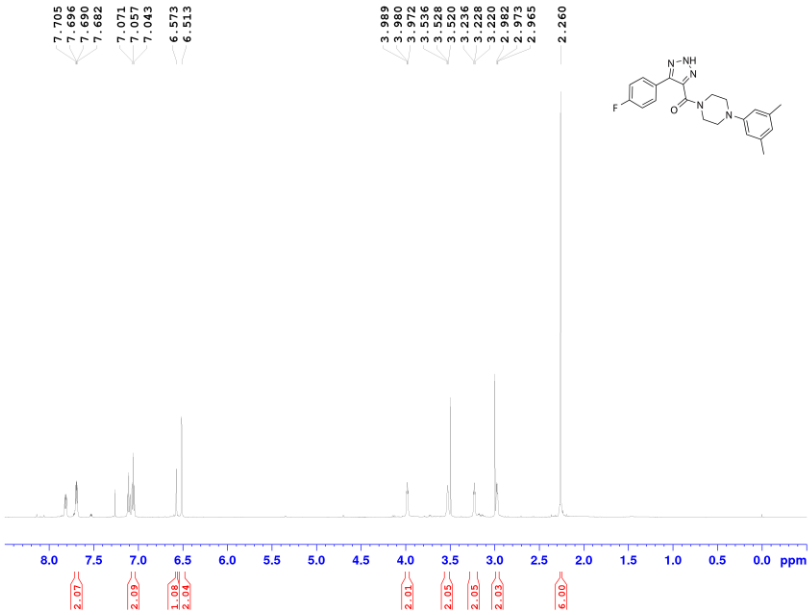

^{13}C NMR of 5-(4-fluorophenyl)-4-(4-(3,5-dimethylphenyl)piperazine-1-carbonyl)-2*H*-1,2,3-triazole (**7n**)

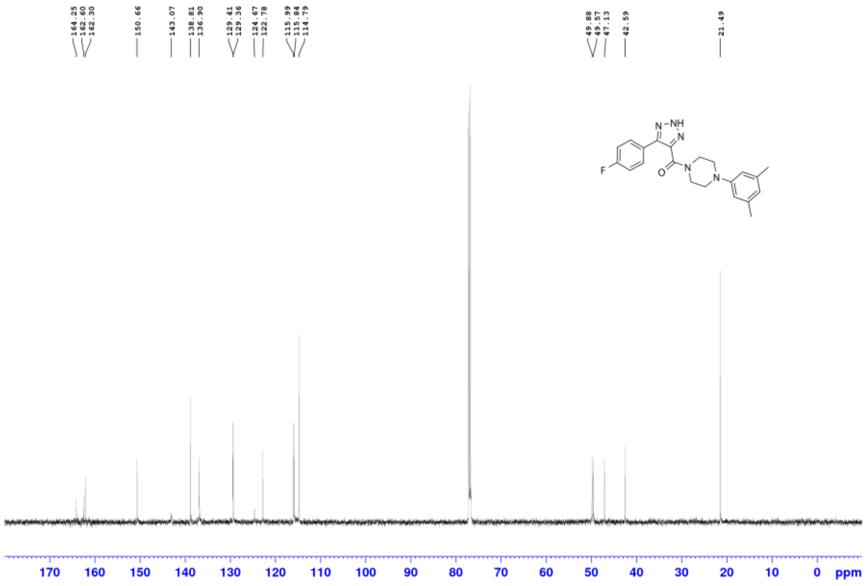

^{1}H NMR of 5-(4-fluorophenyl)-4-(4-(3,5-dimethoxyphenyl)piperazine-1-carbonyl)-2*H*-1,2,3-triazole (**7o**)

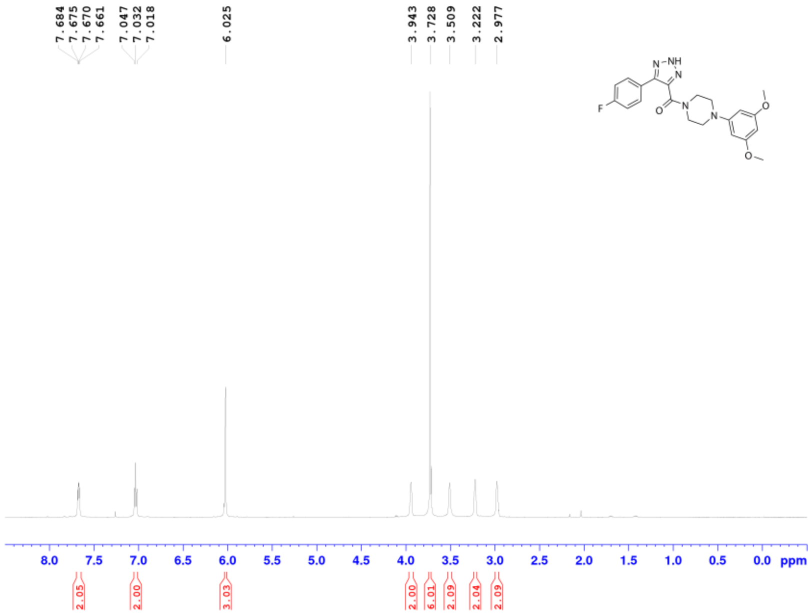

^{13}C NMR of 5-(4-fluorophenyl)-4-(4-(3,5-dimethoxyphenyl)piperazine-1-carbonyl)-2*H*-1,2,3-triazole (**7o**)

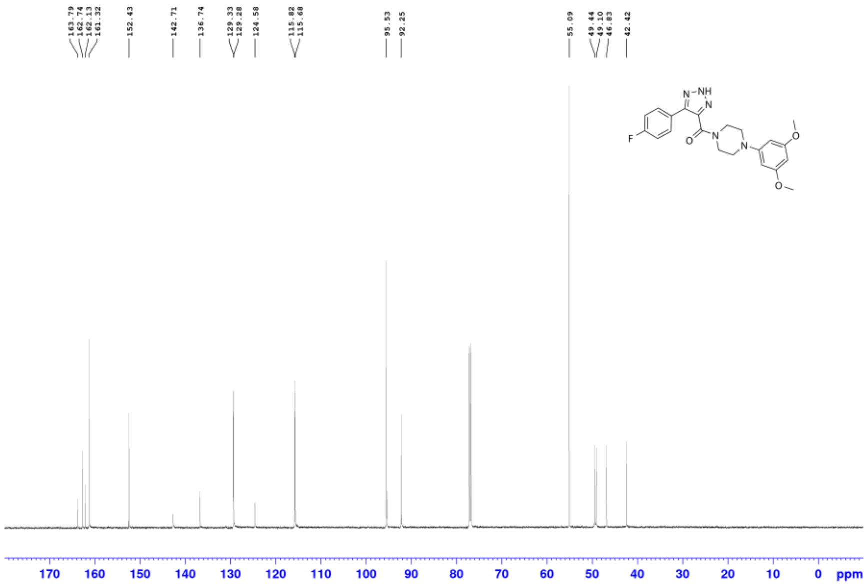

^{1}H NMR of 5-(2-chlorophenyl)-4-(4-(3,5-dimethoxyphenyl)piperazine-1-carbonyl)-2*H*-1,2,3-triazole (**7p**)

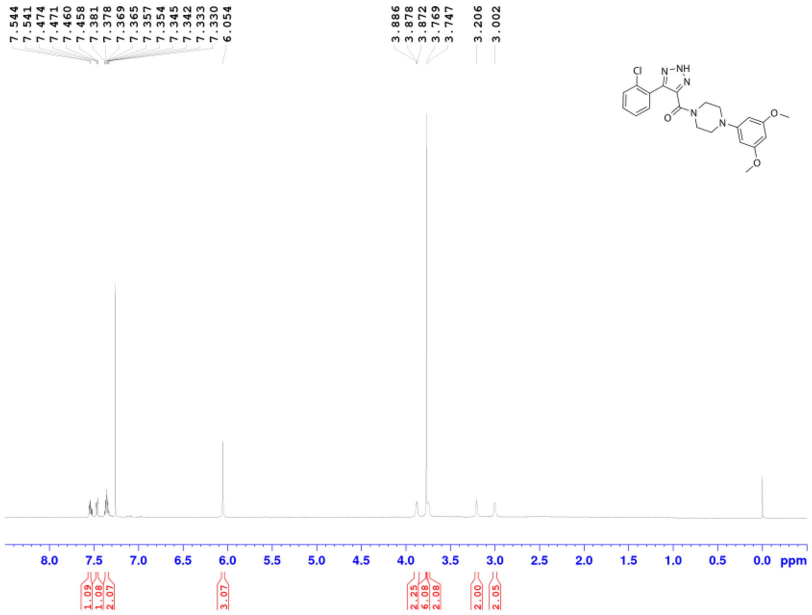

^{13}C NMR of 5-(2-chlorophenyl)-4-(4-(3,5-dimethoxyphenyl)piperazine-1-carbonyl)-2*H*-1,2,3-triazole (**7p**)

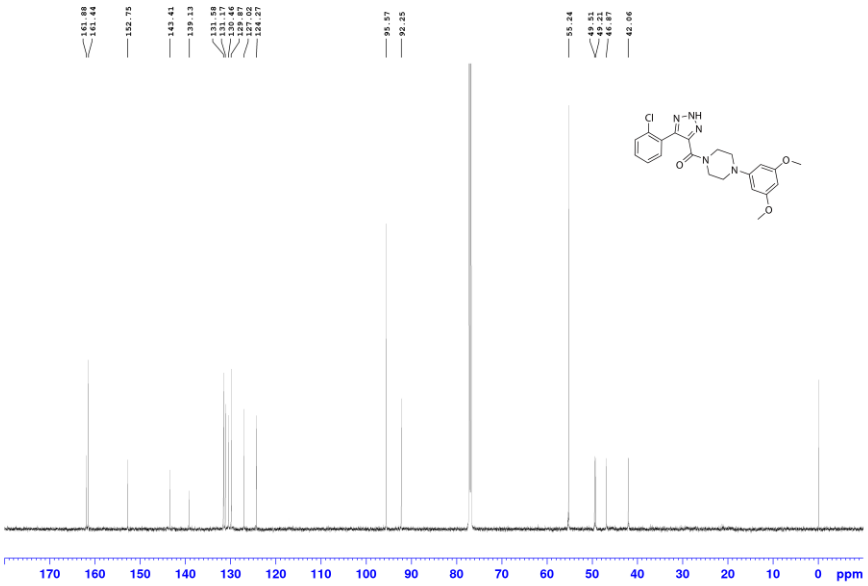

^{1}H NMR of 5-(3-chlorophenyl)-4-(4-(3,5-dimethylphenyl)piperazine-1-carbonyl)-2*H*-1,2,3-triazole (**7q**)

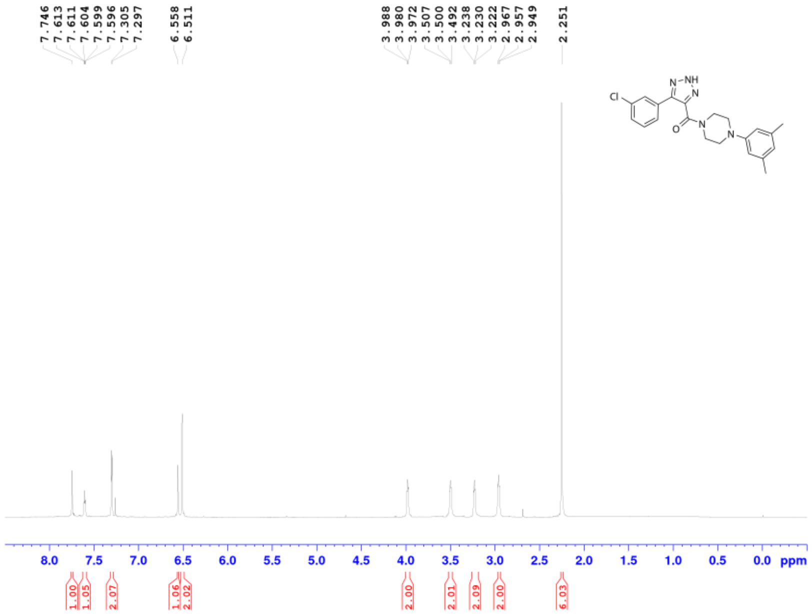

^{13}C NMR of 5-(3-chlorophenyl)-4-(4-(3,5-dimethylphenyl)piperazine-1-carbonyl)-2*H*-1,2,3-triazole (**7q**)

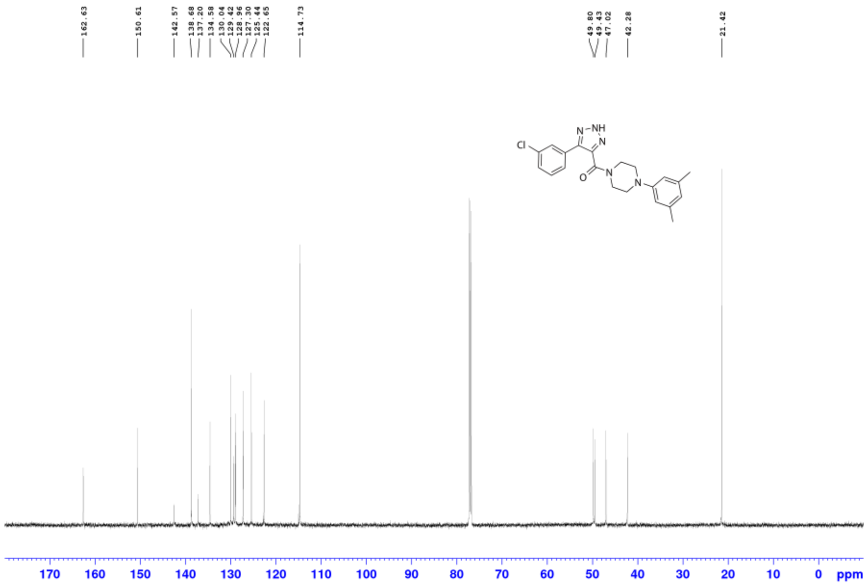

^{1}H NMR of 5-(3-chlorophenyl)-4-(4-(3,5-dimethoxyphenyl)piperazine-1-carbonyl)-2*H*-1,2,3-triazole (**7r**)

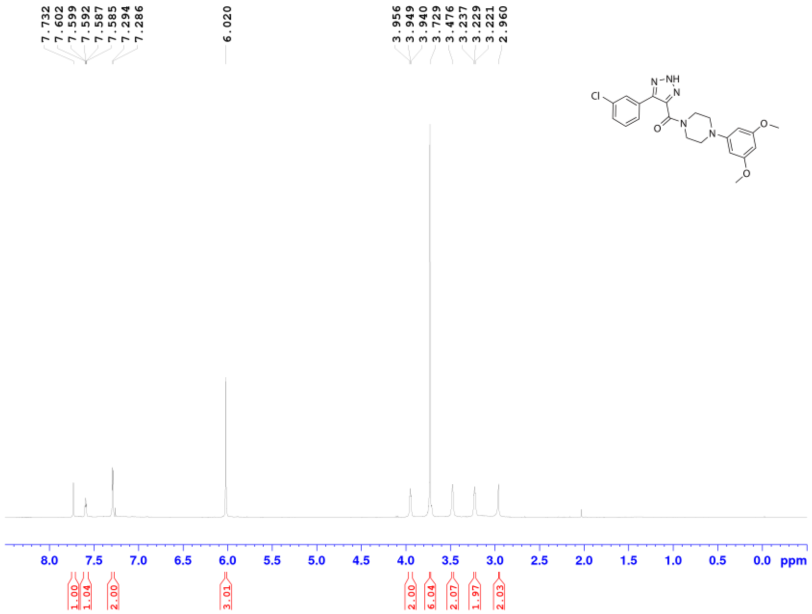

^{13}C NMR of 5-(3-chlorophenyl)-4-(4-(3,5-dimethoxyphenyl)piperazine-1-carbonyl)-2*H*-1,2,3-triazole (**7r**)

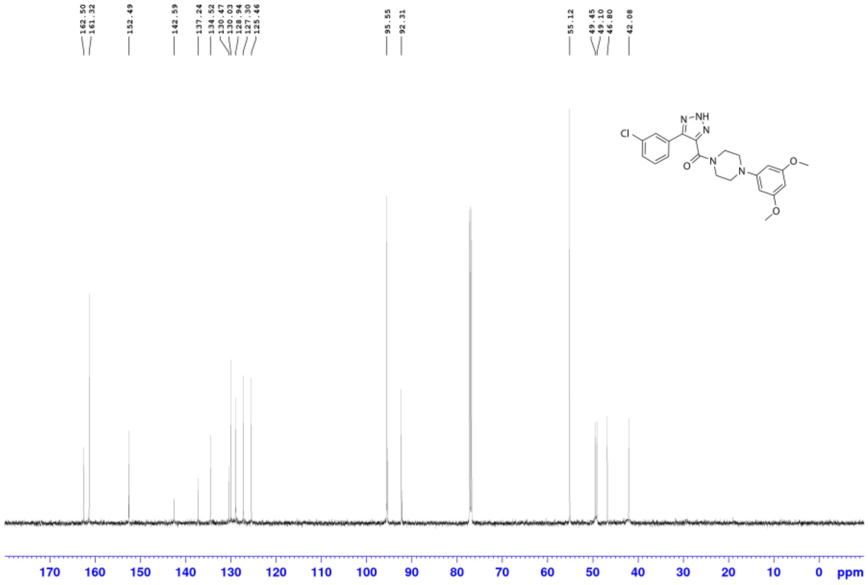

^{1}H NMR of 5-(3-bromophenyl)-4-(4-(3,5-dimethoxyphenyl)piperazine-1-carbonyl)-2*H*-1,2,3-triazole (**7s**)

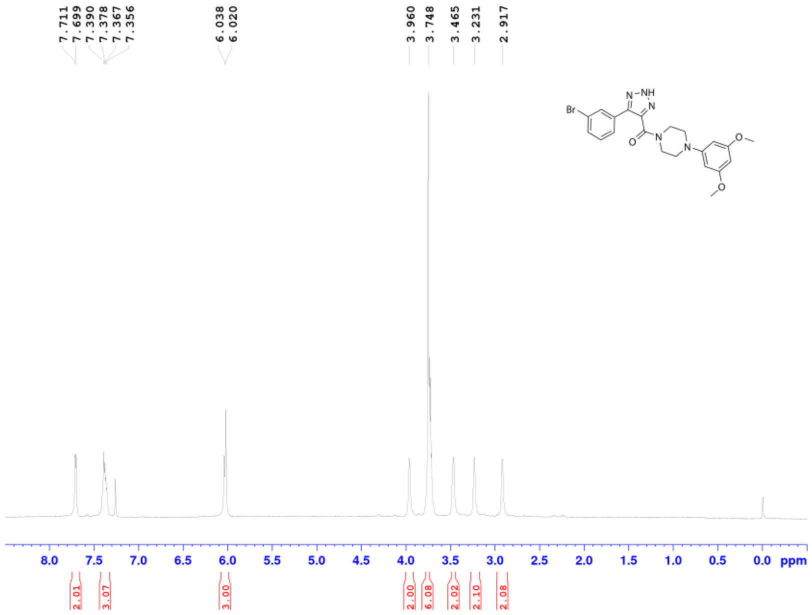

^{13}C NMR of 5-(3-bromophenyl)-4-(4-(3,5-dimethoxyphenyl)piperazine-1-carbonyl)-2*H*-1,2,3-triazole (**7s**)

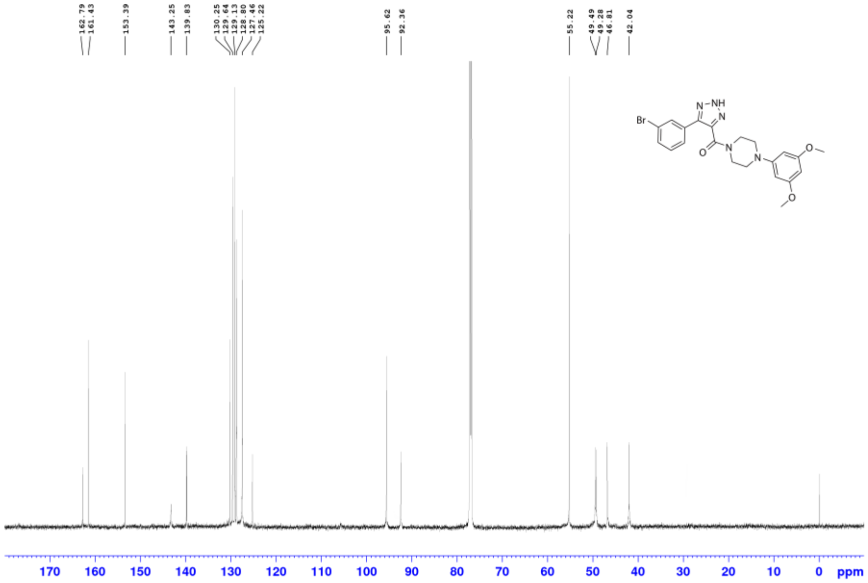

^{1}H NMR of 5-(3-methylphenyl)-4-(4-(3,5-dimethoxyphenyl)piperazine-1-carbonyl)-2*H*-1,2,3-triazole (**7t**)

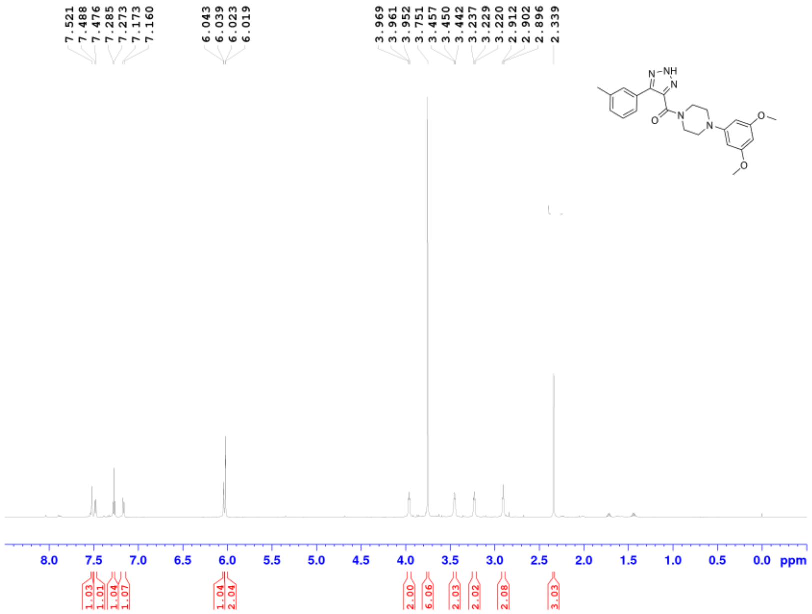

^{13}C NMR of 5-(3-methylphenyl)-4-(4-(3,5-dimethoxyphenyl)piperazine-1-carbonyl)-2*H*-1,2,3-triazole (**7t**)

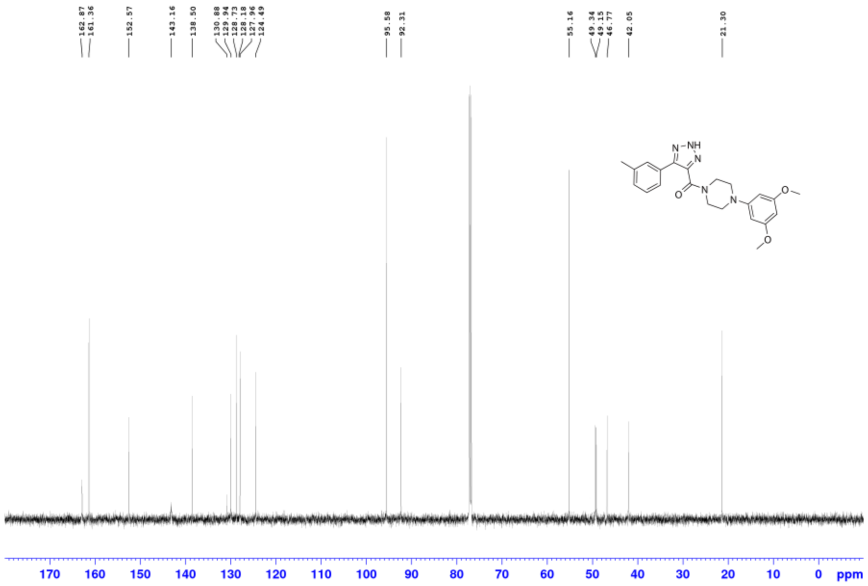

^{1}H NMR of 2-methyl-5-phenyl-4-(4-phenylpiperazine-1-carbonyl)-2*H*-1,2,3-triazole (**8a**)

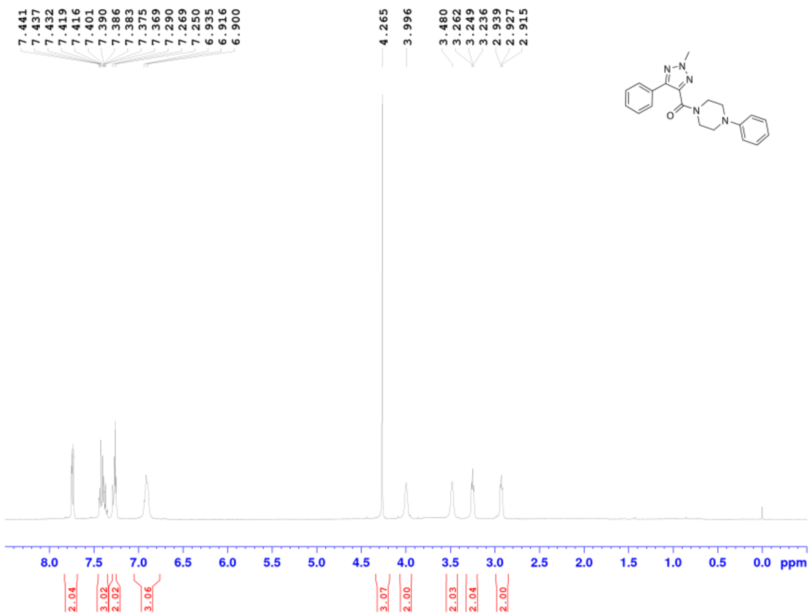

^{13}C NMR of 2-methyl-5-phenyl-4-(4-phenylpiperazine-1-carbonyl)-2*H*-1,2,3-triazole (**8a**)

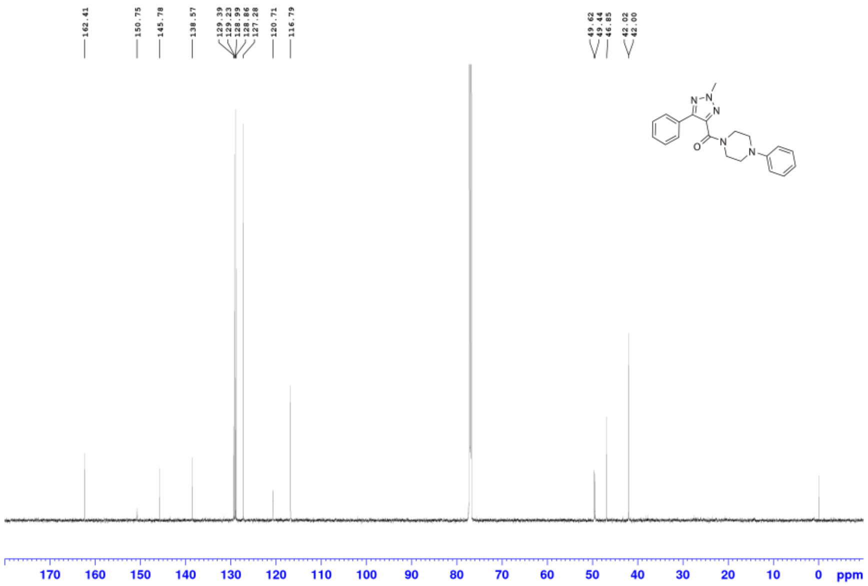

^{1}H NMR of 2-methyl-5-phenyl-4-(4-(3-chlorophenyl)piperazine-1-carbonyl)-2*H*-1,2,3-triazole (**8b**)

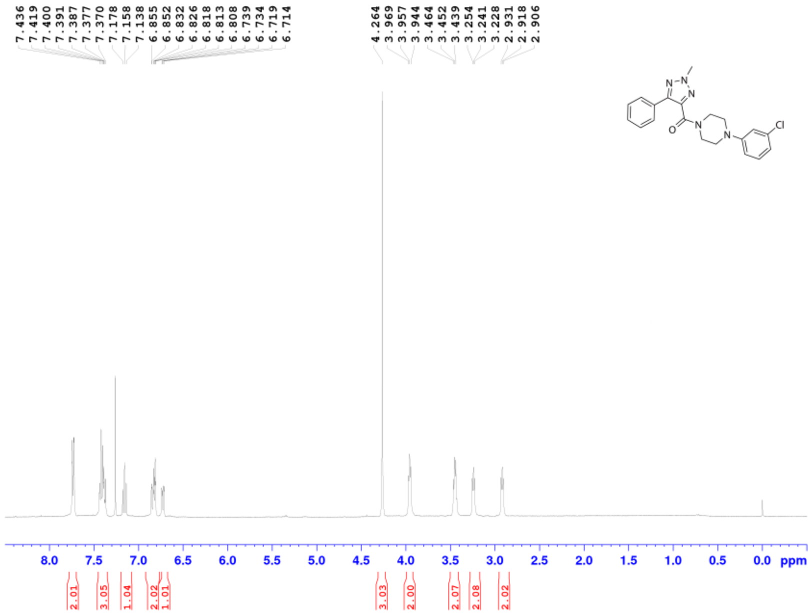

^{13}C NMR of 2-methyl-5-phenyl-4-(4-(3-chlorophenyl)piperazine-1-carbonyl)-2*H*-1,2,3-triazole (**8b**)

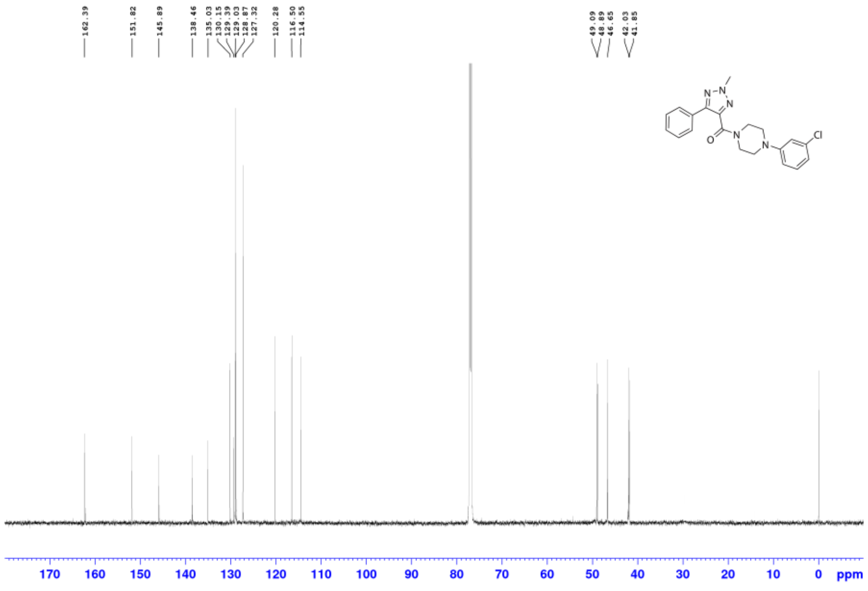

^{1}H NMR of 2-ethyl-5-phenyl-4-(4-(3-chlorophenyl)piperazine-1-carbonyl)-2*H*-1,2,3-triazole (**8c**)

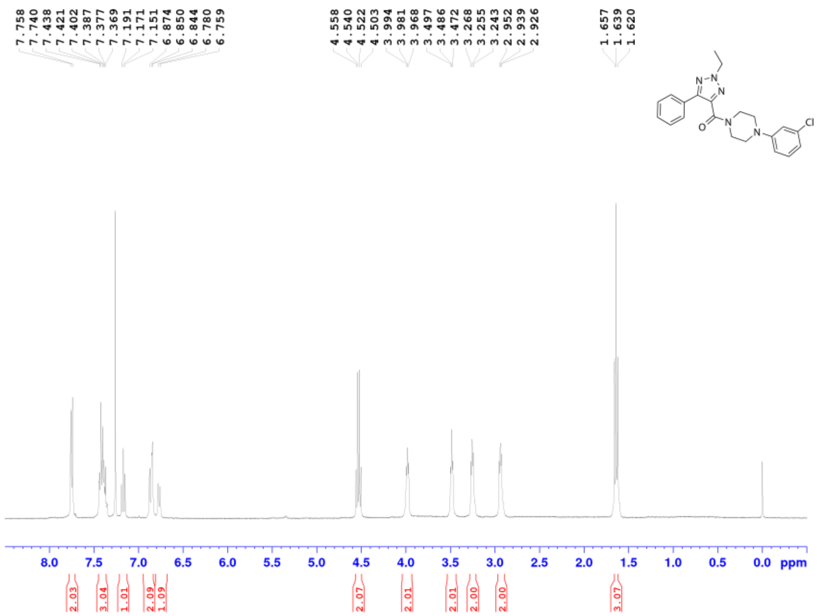

^{13}C NMR of 2-ethyl-5-phenyl-4-(4-(3-chlorophenyl)piperazine-1-carbonyl)-2*H*-1,2,3-triazole (**8c**)

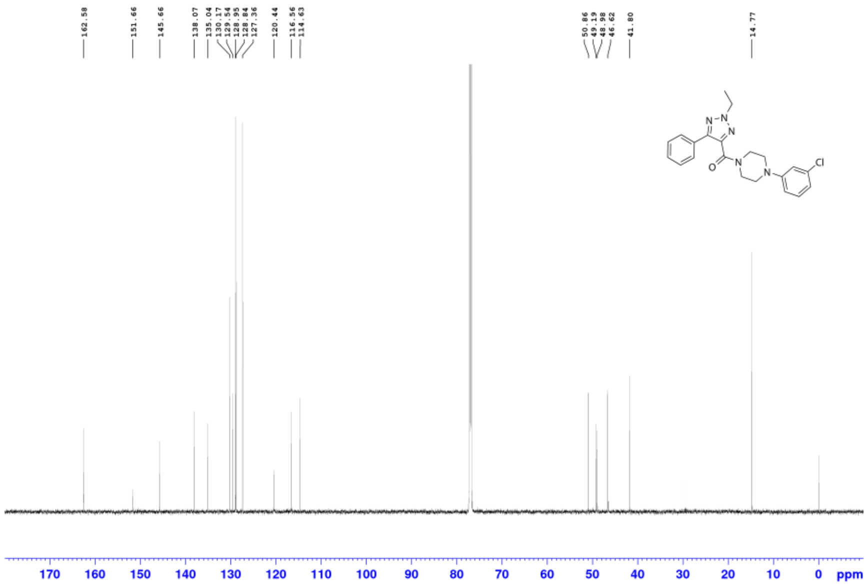

^{1}H NMR of 2-allyl-5-phenyl-4-(4-(3-chlorophenyl)piperazine-1-carbonyl)-2*H*-1,2,3-triazole (**8d**)

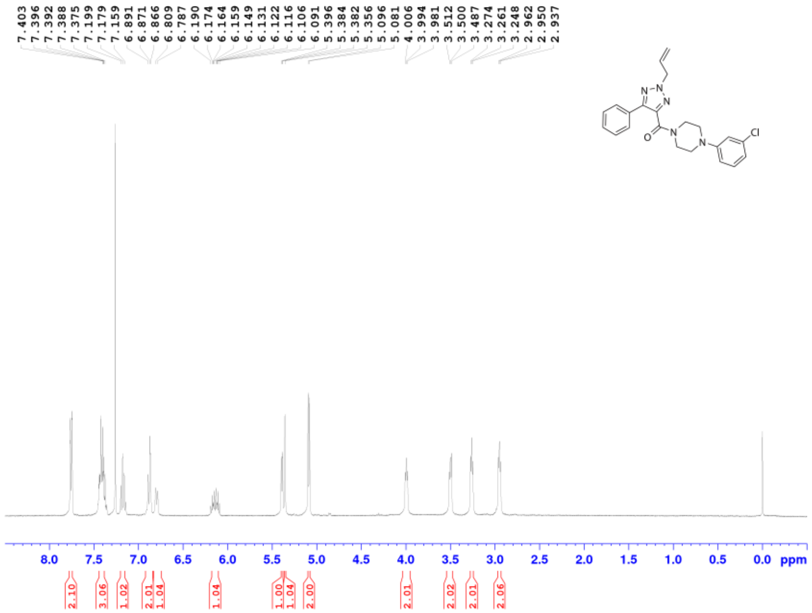

^{13}C NMR of 2-allyl-5-phenyl-4-(4-(3-chlorophenyl)piperazine-1-carbonyl)-2*H*-1,2,3-triazole (**8d**)

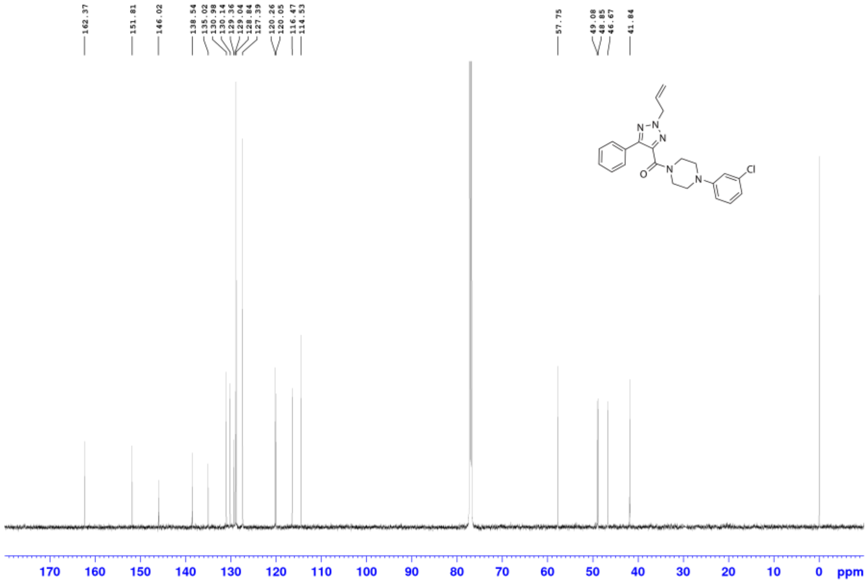

^{1}H NMR of 2-isopropyl-5-phenyl-4-(4-(3-chlorophenyl)piperazine-1-carbonyl)-2*H*-1,2,3-triazole (**8e**)

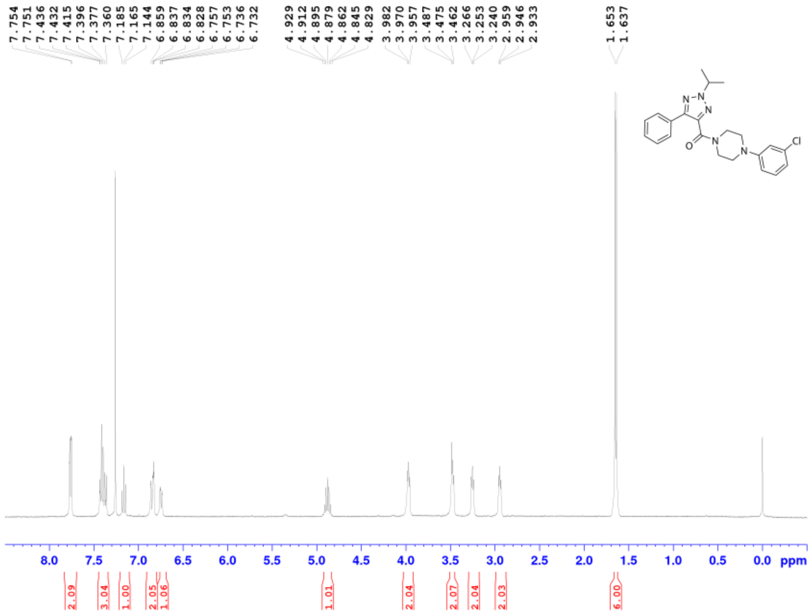

^{13}C NMR of 2-isopropyl-5-phenyl-4-(4-(3-chlorophenyl)piperazine-1-carbonyl)-2*H*-1,2,3-triazole (**8e**)

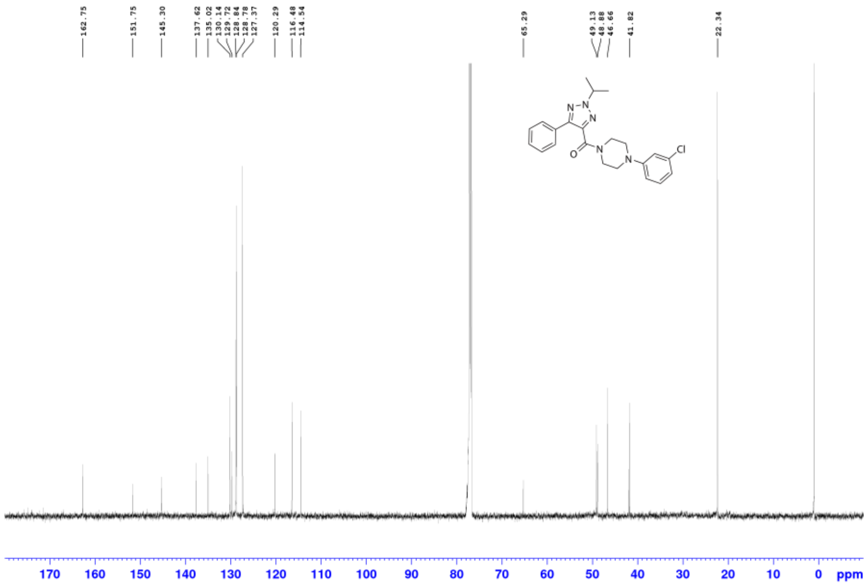

^{1}H NMR of 2-butyl-5-phenyl-4-(4-(3,5-chlorophenyl)piperazine-1-carbonyl)-2*H*-1,2,3-triazole (**8f**)

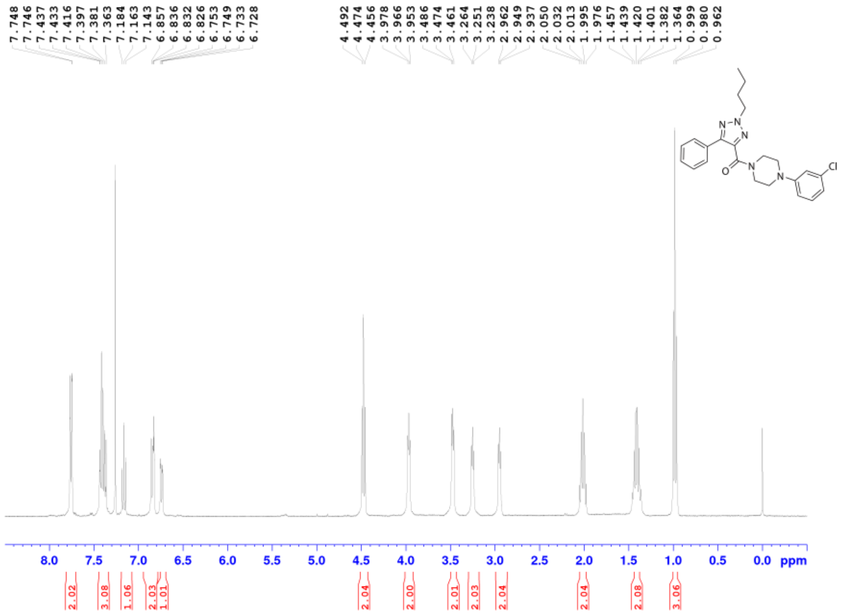

^{13}C NMR of 2-butyl-5-phenyl-4-(4-(3,5-chlorophenyl)piperazine-1-carbonyl)-2*H*-1,2,3-triazole (**8f**)

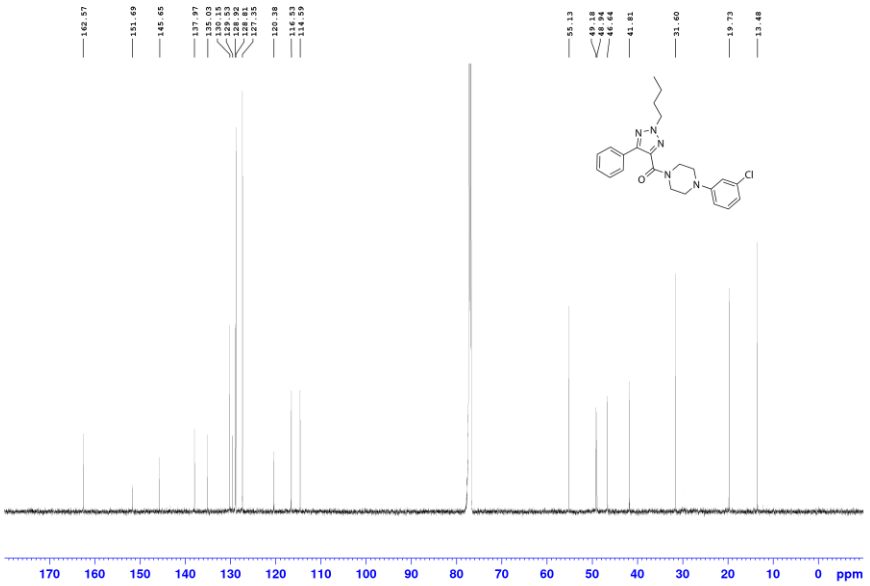

^{1}H NMR of 2-methyl-5-phenyl-4-(4-(3,5-dimethylphenyl)piperazine-1-carbonyl)-2*H*-1,2,3-triazole (**8g**)

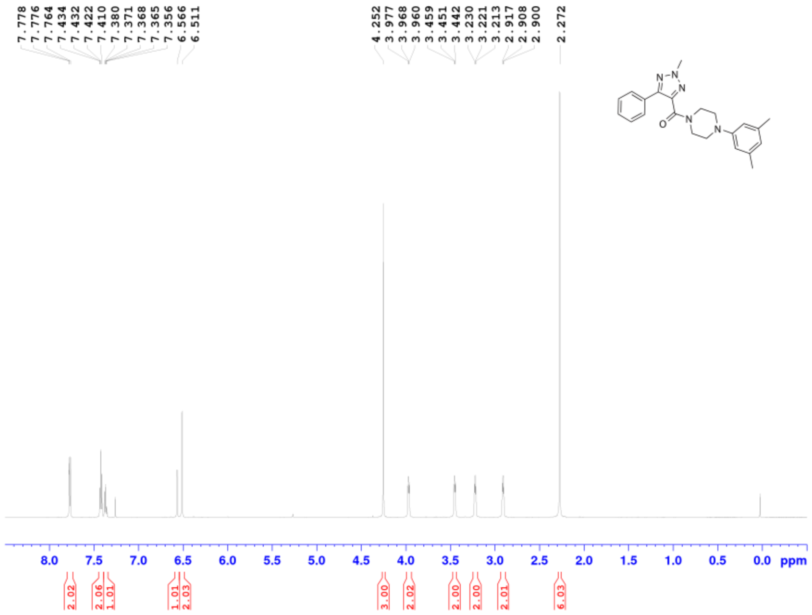

^{13}C NMR of 2-methyl-5-phenyl-4-(4-(3,5-dimethylphenyl)piperazine-1-carbonyl)-2*H*-1,2,3-triazole (**8g**)

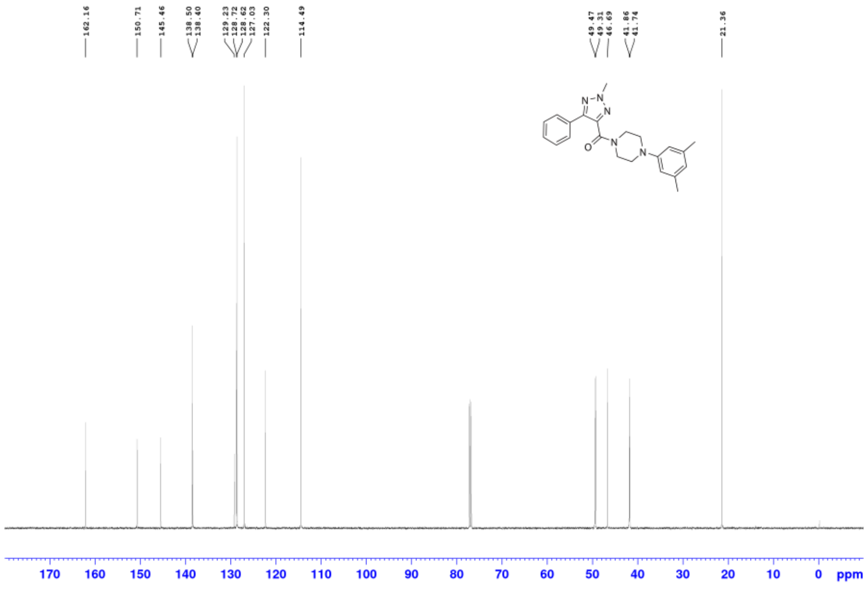

^{1}H NMR of 2-methyl-5-phenyl-4-(4-(3,5-dimethoxyphenyl)piperazine-1-carbonyl)-2*H*-1,2,3-triazole (**8h**)

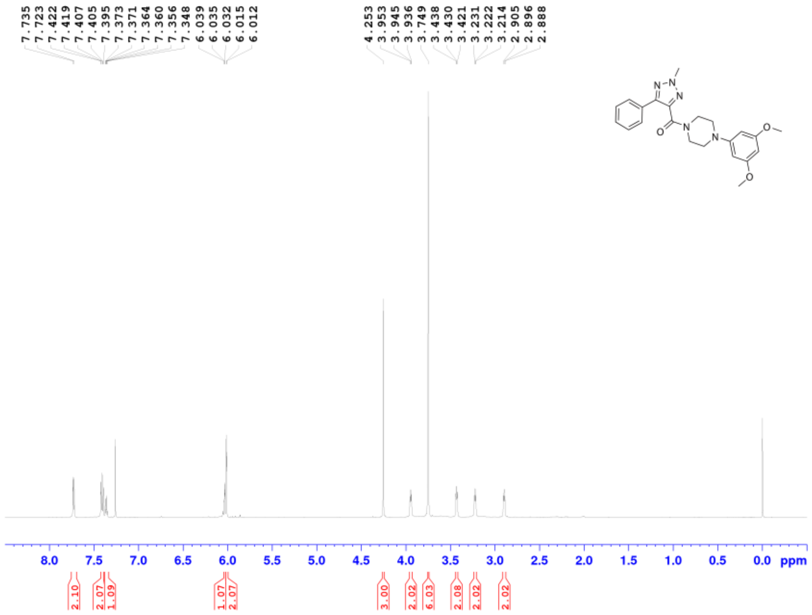

^{13}C NMR of 2-methyl-5-phenyl-4-(4-(3,5-dimethoxyphenyl)piperazine-1-carbonyl)-2*H*-1,2,3-triazole (**8h**)

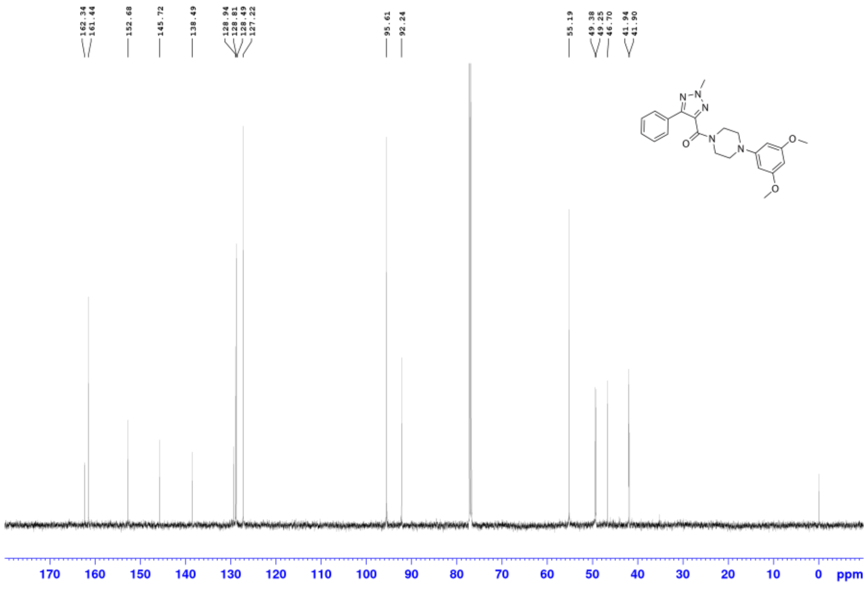

^{1}H NMR of 2-methyl-5-(2-fluorophenyl)-4-(4-(3,5-dimethylphenyl)piperazine-1-carbonyl)-2*H*-1,2,3-triazole (**8i**)

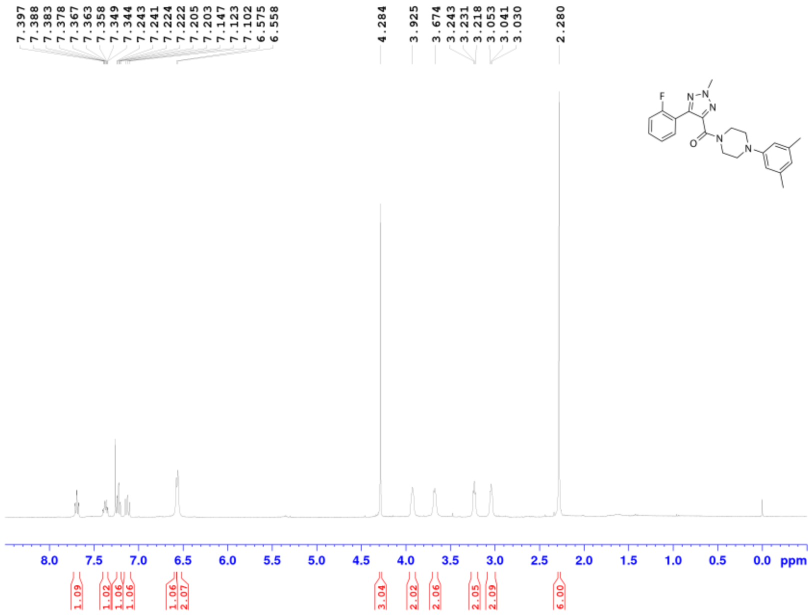

^{13}C NMR of 2-methyl-5-(2-fluorophenyl)-4-(4-(3,5-dimethylphenyl)piperazine-1-carbonyl)-2*H*-1,2,3-triazole (**8i**)

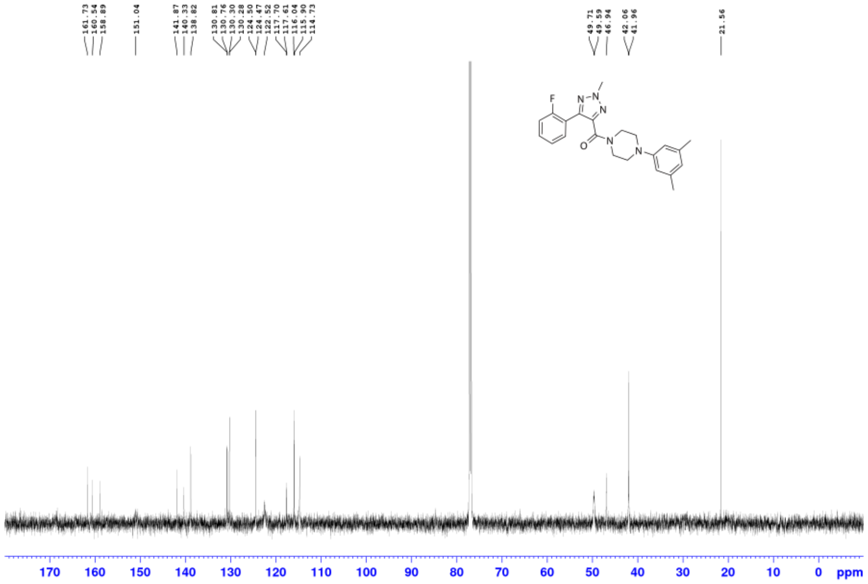

^{1}H NMR of 2-ethyl-5-(2-fluorophenyl)-4-(4-(3,5-dimethylphenyl)piperazine-1-carbonyl)-2*H*-1,2,3-triazole (**8j**)

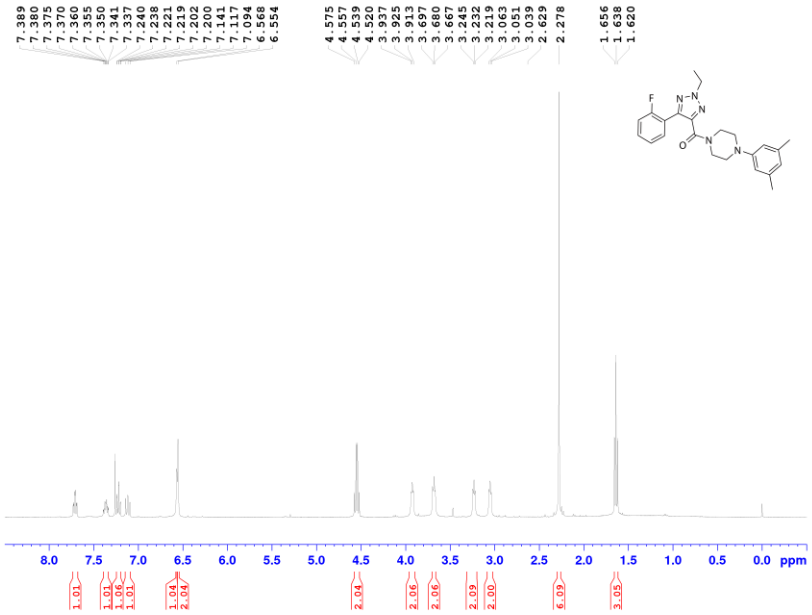

^{13}C NMR of 2-ethyl-5-(2-fluorophenyl)-4-(4-(3,5-dimethylphenyl)piperazine-1-carbonyl)-2*H*-1,2,3-triazole (**8j**)

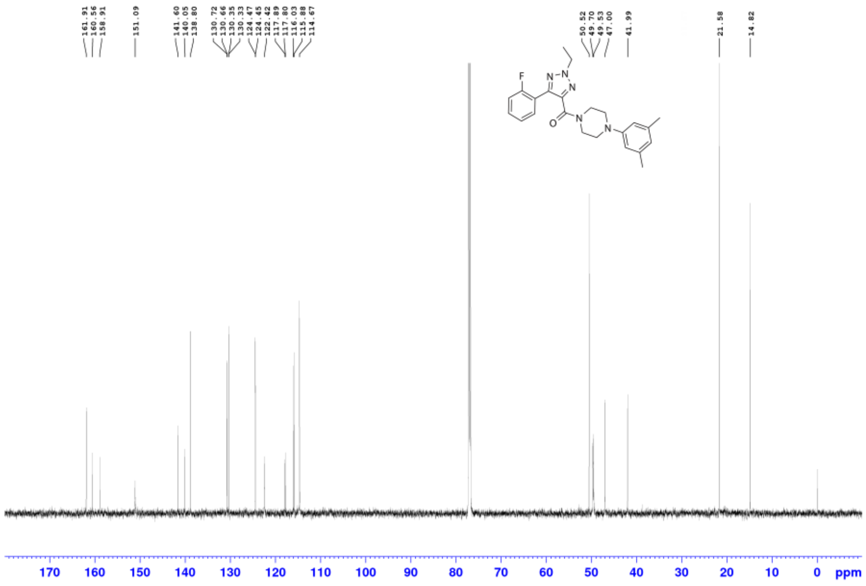

^{1}H NMR of 2-methyl-5-(2-fluorophenyl)-4-(4-(3,5-dimethoxyphenyl)piperazine-1-carbonyl)-2*H*-1,2,3-triazole (**8k**)

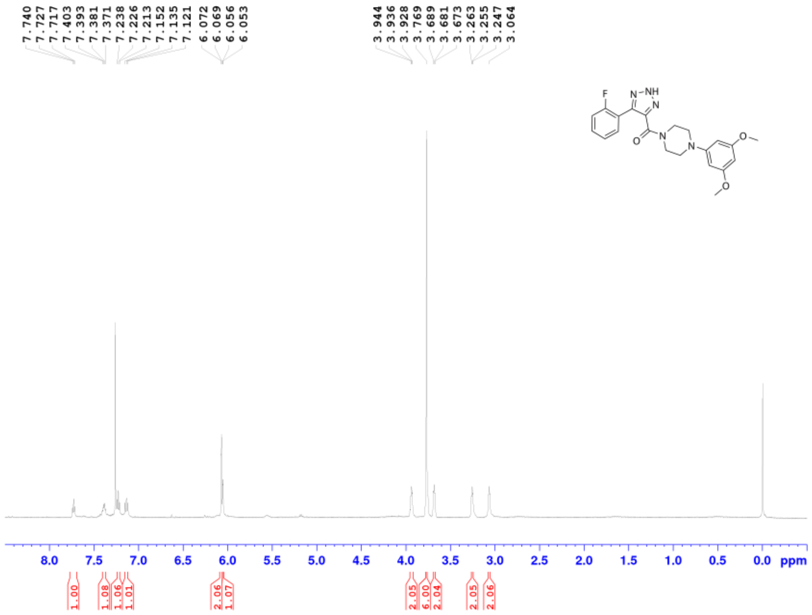

^{13}C NMR of 2-methyl-5-(2-fluorophenyl)-4-(4-(3,5-dimethoxyphenyl)piperazine-1-carbonyl)-2*H*-1,2,3-triazole (**8k**)

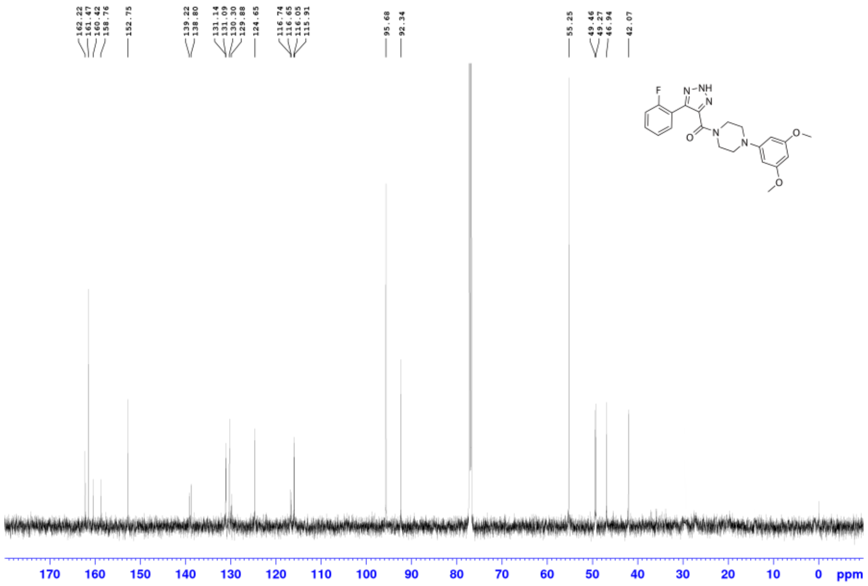

^{1}H NMR of 2-methyl-5-(4-fluorophenyl)-4-(4-(3,5-dimethylphenyl)piperazine-1-carbonyl)-2*H*-1,2,3-triazole (**8l**)

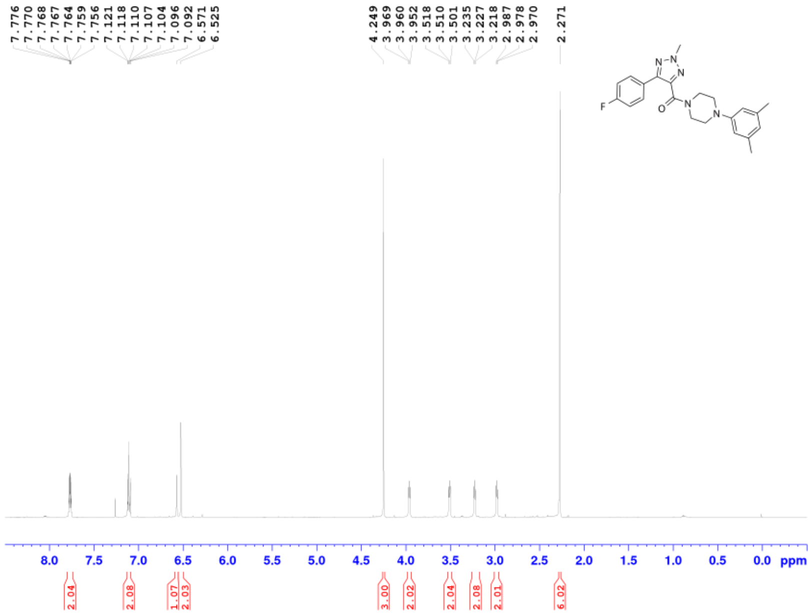

^{13}C NMR of 2-methyl-5-(4-fluorophenyl)-4-(4-(3,5-dimethylphenyl)piperazine-1-carbonyl)-2*H*-1,2,3-triazole (**8l**)

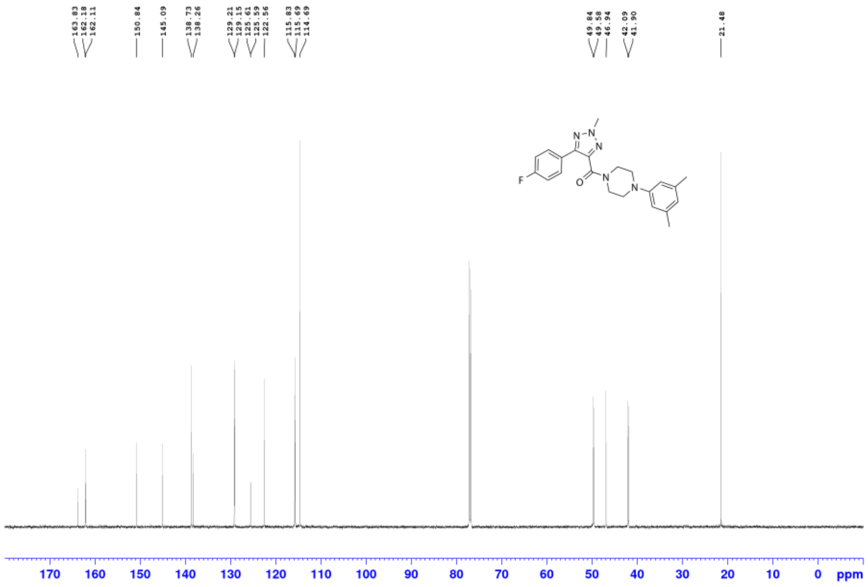

^{1}H NMR of 2-methyl-5-(4-fluorophenyl)-4-(4-(3,5-dimethoxyphenyl)piperazine-1-carbonyl)-2*H*-1,2,3-triazole (**8m**)

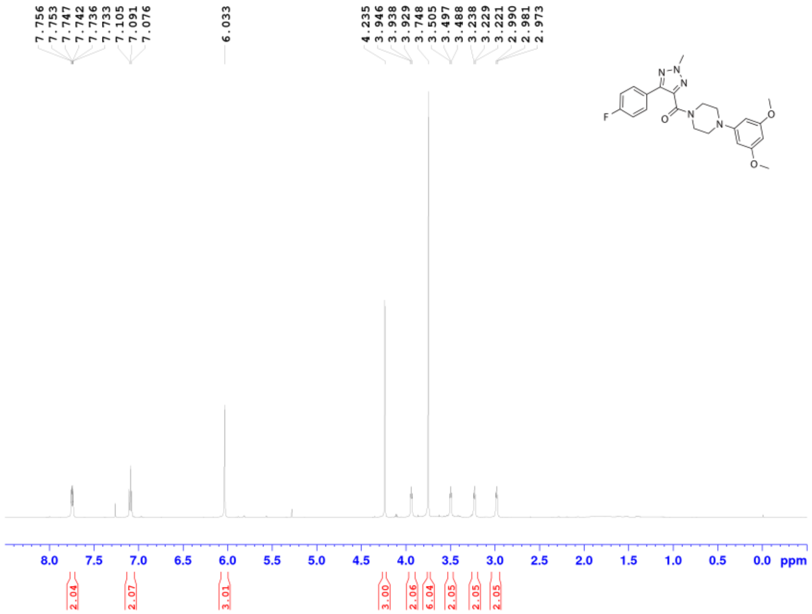

^{13}C NMR of 2-methyl-5-(4-fluorophenyl)-4-(4-(3,5-dimethoxyphenyl)piperazine-1-carbonyl)-2*H*-1,2,3-triazole (**8m**)

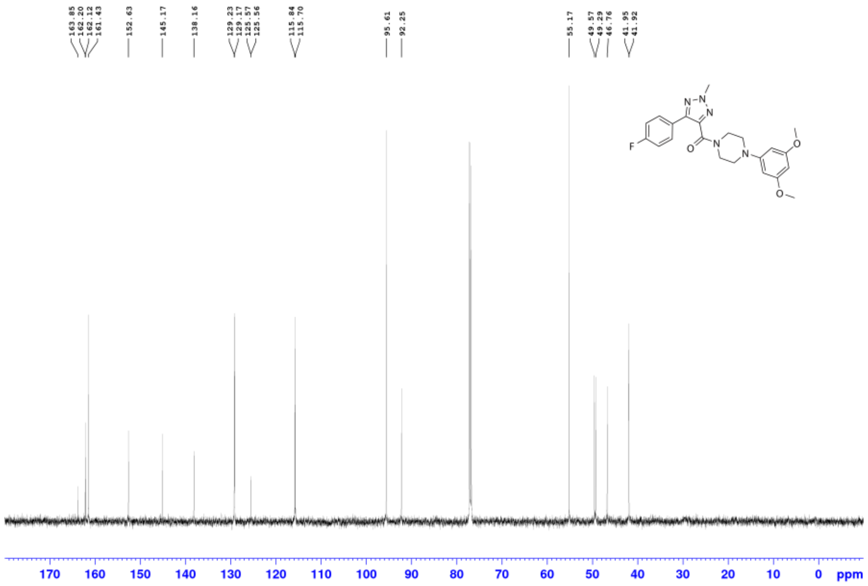

^1H NMR of 2-methyl-5-(3-chlorophenyl)-4-(4-(3,5-dimethylphenyl)piperazine-1-carbonyl)-2*H*-1,2,3-triazole (**8n**)

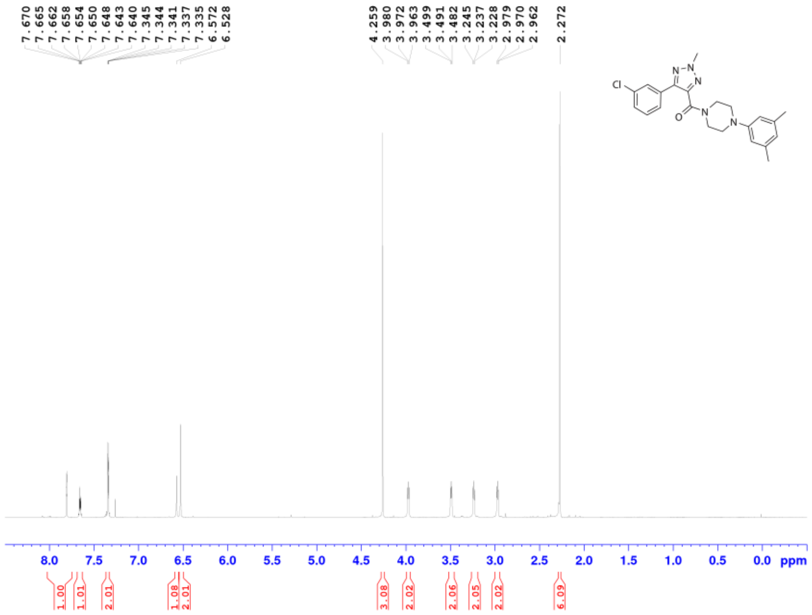

^{13}C NMR of 2-methyl-5-(3-chlorophenyl)-4-(4-(3,5-dimethylphenyl)piperazine-1-carbonyl)-2*H*-1,2,3-triazole (**8n**)

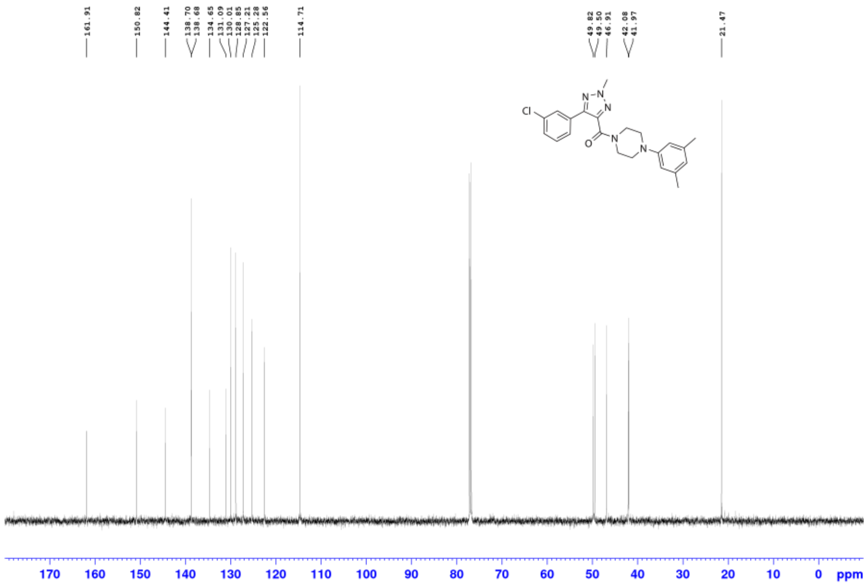

^{1}H NMR of 2-methyl-5-(3-chlorophenyl)-4-(4-(3,5-dimethoxyphenyl)piperazine-1-carbonyl)-2*H*-1,2,3-triazole (**8o**)

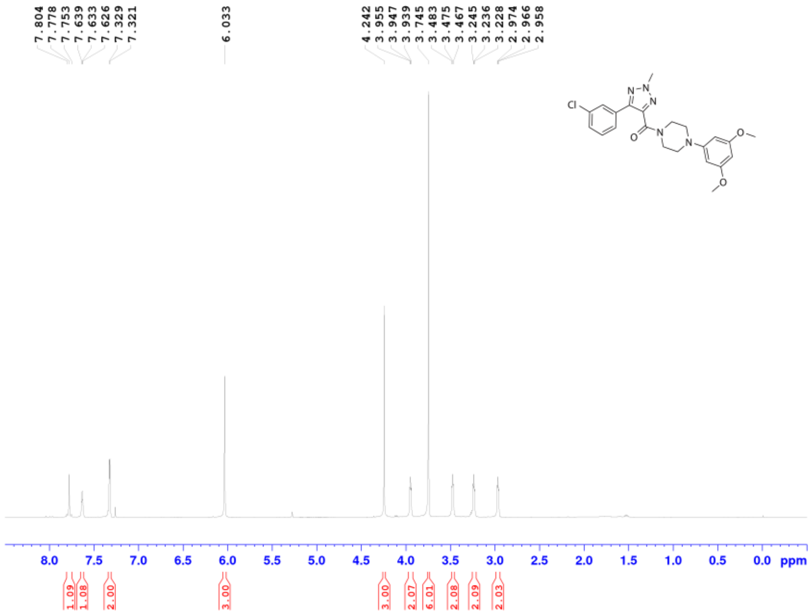

^{13}C NMR of 2-methyl-5-(3-chlorophenyl)-4-(4-(3,5-dimethoxyphenyl)piperazine-1-carbonyl)-2*H*-1,2,3-triazole (**8o**)

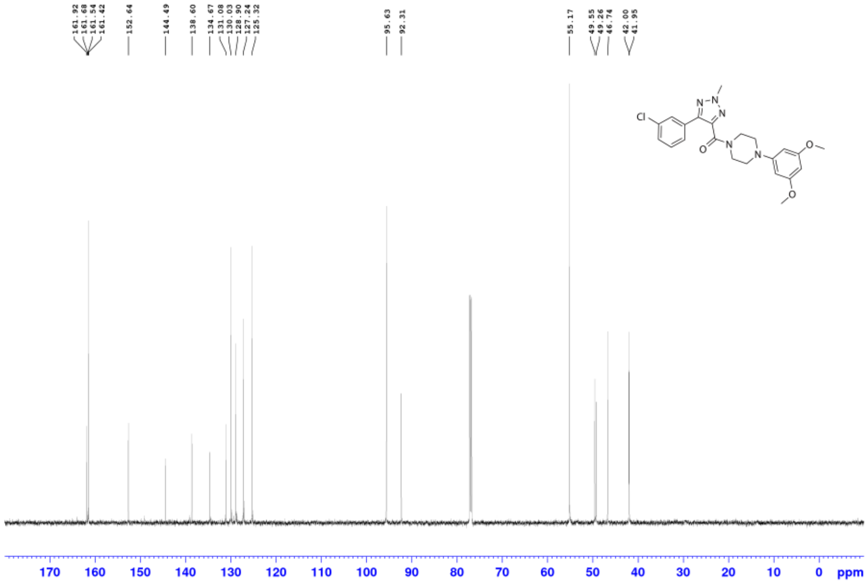

^{1}H NMR of 4,5-dibromo-2-methyl-2*H*-1,2,3-triazole (**13**)

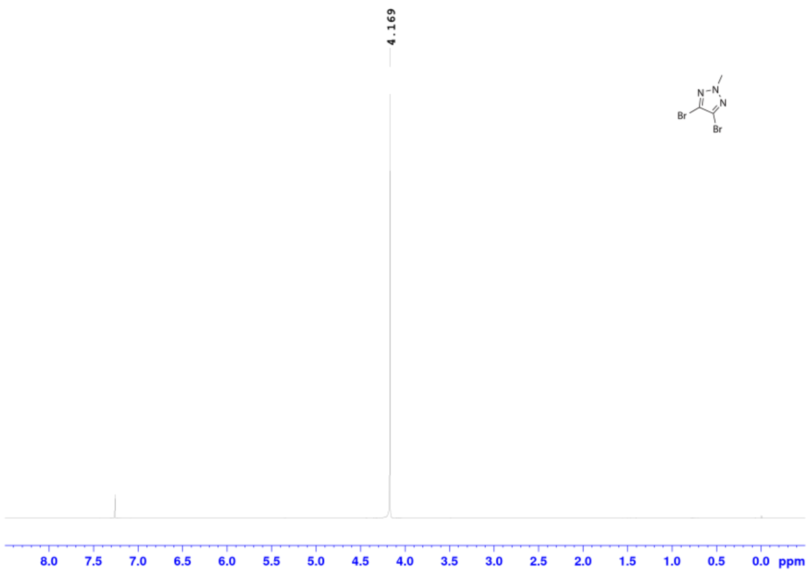

^{1}H NMR of 5-bromo-2-methyl-2*H*-1,2,3-triazole-4-carbaldehyde (**14**)

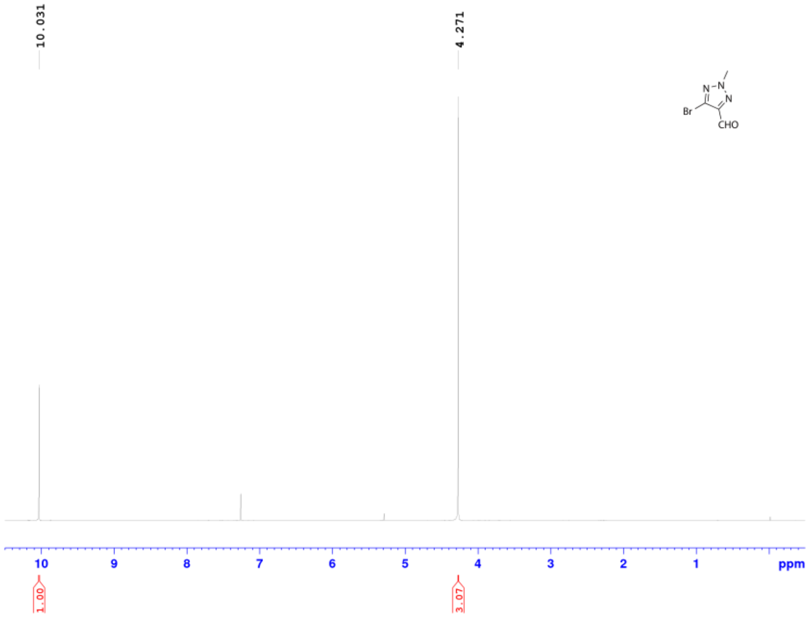

^{1}H NMR of 2-methyl-5-phenyl-2H-1,2,3-triazole-4-carbaldehyde (**15a**)

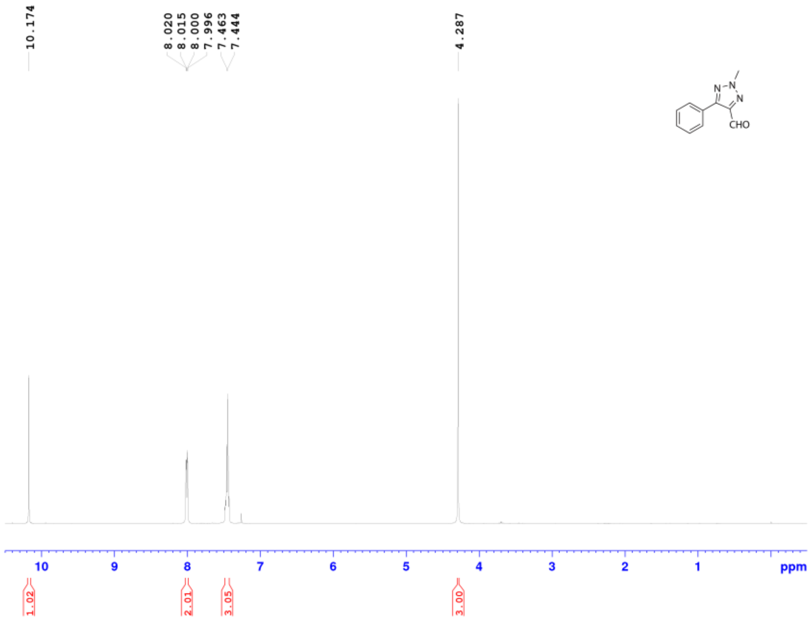

^{1}H NMR of 5-(2-fluorophenyl)-2-methyl-2H-1,2,3-triazole-4-carbaldehyde (**15b**)

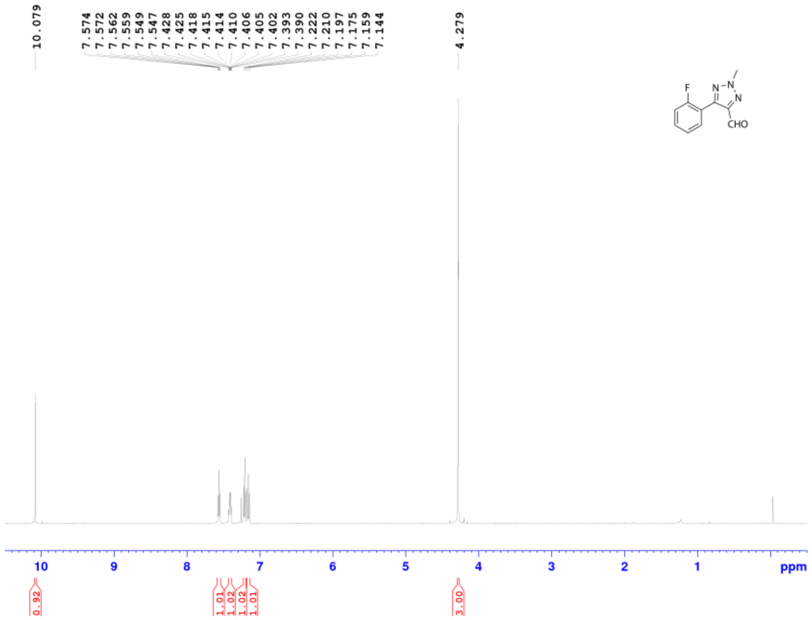

Information of DFT computation

Energy comparison for three tautomers of **7**

	1*H* (a.u.)	2*H* (a.u.)	3*H* (a.u.)	E(1*H*) - E(2*H*) (kJ/mol)	E(3*H*) - E(2*H*) (kJ/mol)
**7a**	-1084.43491	-1084.436671	-1084.43201	4.62	12.24
**7b**	-1544.031948	-1544.033675	-1544.028955	4.53	12.39
**7h**	-1163.070713	-1163.072527	-1163.067757	4.76	12.53
**7j**	-1313.483977	-1313.485726	-1313.481178	4.59	11.94
**7k**	-1262.304008	-1262.306718	-1262.302693	7.11	10.57
**7l**	-1412.717398	-1412.720169	-1412.715935	7.28	11.12
**7n**	-1262.303656	-1262.305807	-1262.301055	5.65	12.47
**7o**	-1412.716982	-1412.719114	-1412.714363	5.60	12.47
**7q**	-1622.665541	-1622.668352	-1622.664616	7.38	9.81
**7r**	-1773.078875	-1773.081723	-1773.077033	7.48	12.31

Coordinate of optimized molecules

Tautomers of **7a**

5-phenyl-4-(4-phenylpiperazine-1-carbonyl)-1*H*-1,2,3-triazole

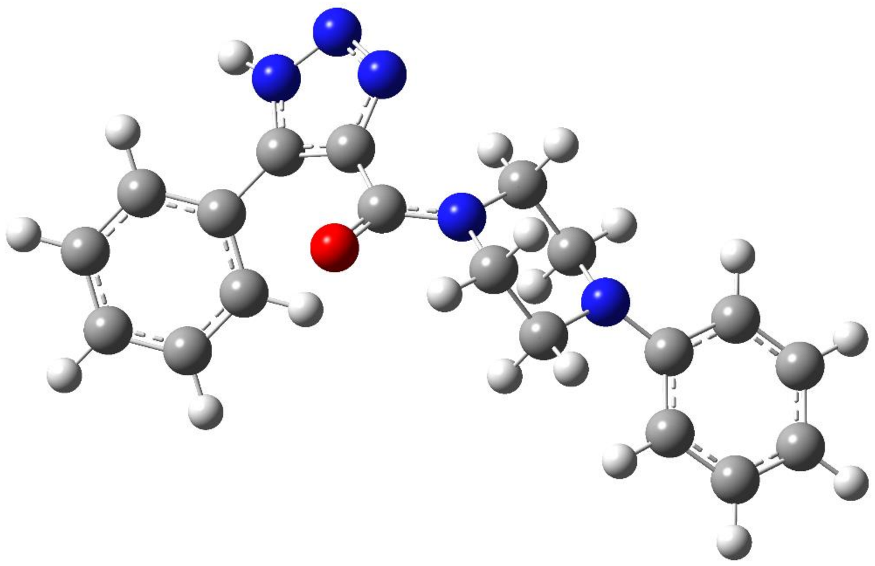

C	5.84546200	1.83722200	-0.38438500
C	5.06626700	2.99432000	-0.44751200
C	3.67241700	2.89199700	-0.46751000
C	3.05836400	1.64206000	-0.42799300
C	3.83488000	0.47312800	-0.36574900
C	5.23581900	0.58380300	-0.34200900
C	3.19727500	-0.84899100	-0.34963400
C	2.01833200	-1.36245700	0.19391200
N	1.93642800	-2.69121300	-0.12029500
N	2.97563000	-3.03608700	-0.82972300
N	3.73252400	-1.93060900	-0.97084900

C	1.08064300	-0.68096900	1.13678800
O	1.54109700	0.10288500	1.97593100
N	-0.24899000	-0.98099200	1.06382000
C	-0.93301000	-1.70264100	-0.01008800
C	-2.01029000	-0.81581900	-0.64008300
N	-2.94520900	-0.31295500	0.37605600
C	-2.24440700	0.43743900	1.41667200
C	-1.16678600	-0.43549500	2.06421700
C	-4.18097600	0.19793000	-0.08759500
C	-4.67308400	1.45748100	0.30269800
C	-5.92386200	1.90250900	-0.13464200
C	-6.70741700	1.11904100	-0.98032500
C	-6.22409800	-0.13192900	-1.37868400
C	-4.98658600	-0.59021400	-0.93664100
H	6.92901000	1.90775900	-0.36090000
H	5.54155700	3.97051100	-0.47937500
H	3.06078000	3.78804600	-0.52010500
H	1.97650400	1.56975000	-0.45755900
H	5.85147000	-0.30814100	-0.26498600
H	-0.21825200	-2.00877300	-0.77020100
H	-1.39688200	-2.60598500	0.40723900
H	-1.52270100	0.02366000	-1.16768800
H	-2.55109200	-1.40395500	-1.38285100
H	-2.95872700	0.72780800	2.19190100
H	-1.78016100	1.35806200	1.01944600
H	-1.64633200	-1.27319200	2.58768200
H	-0.58532100	0.14516800	2.77902100
H	-4.08181000	2.10640600	0.93865100
H	-6.27605800	2.88014600	0.18499500
H	-7.67674400	1.47114800	-1.32119400
H	-4.65137700	-1.58040100	-1.22987300
H	4.57307400	-1.97283600	-1.53467600
H	-6.82205800	-0.76575200	-2.02865800

5-phenyl-4-(4-phenylpiperazine-1-carbonyl)-2*H*-1,2,3-triazole

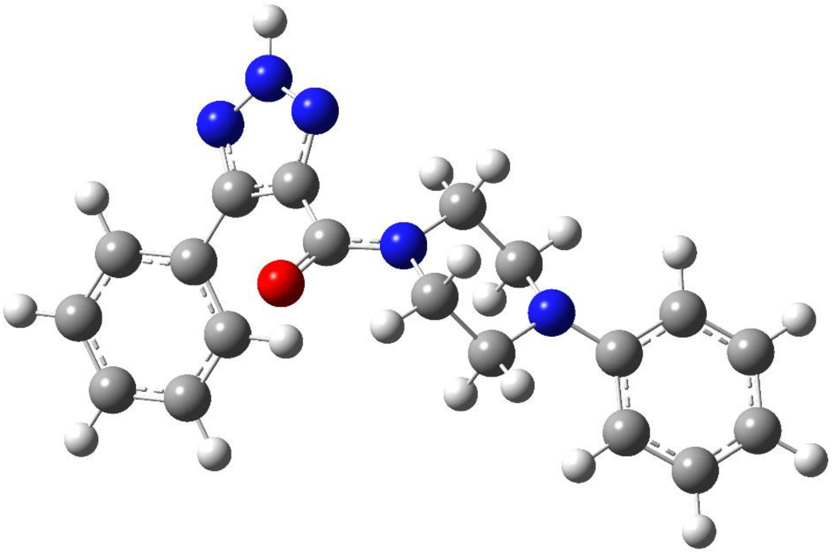

C	5.59748700	2.12237600	-0.50956700
C	4.72077900	3.19948300	-0.35489000
C	3.35160300	2.96071200	-0.21388800
C	2.85992300	1.65578400	-0.22459200
C	3.73404400	0.56725200	-0.37314400
C	5.10939300	0.81678000	-0.51830700
C	3.23552200	-0.81795600	-0.40482200
C	2.08544800	-1.42051900	0.17749200
N	2.06737400	-2.71875600	-0.15619000
N	3.15337400	-2.85992700	-0.89945400
N	3.88629000	-1.77098700	-1.09130400
C	1.13072100	-0.84721400	1.18007400
O	1.59026900	-0.19625200	2.12483500
N	-0.20068100	-1.09395100	1.02749900
C	-0.85431300	-1.70999100	-0.12812300
C	-1.92099500	-0.77030100	-0.69565400
N	-2.88024900	-0.37038100	0.34319600
C	-2.20595500	0.28336200	1.46308100
C	-1.14173000	-0.64389900	2.05452200
C	-4.10853900	0.17806700	-0.09885600
C	-4.61082300	1.39934200	0.38760700
C	-5.85371500	1.87923400	-0.03519400
C	-6.61821300	1.17009100	-0.96013600
C	-6.12422600	-0.04205000	-1.45411900
C	-4.89478700	-0.53553300	-1.02771800
H	6.66410200	2.29809200	-0.61873400
H	5.10150000	4.21696400	-0.34502200
H	2.66177100	3.79240400	-0.10084600
H	1.79254200	1.48589300	-0.13096700
H	5.79131500	-0.02017800	-0.63088900

H	-0.11848600	-1.93529200	-0.89722600
H	-1.32198400	-2.65186100	0.18701800
H	-1.42579800	0.11690300	-1.12939100
H	-2.44243000	-1.28634800	-1.50304200
H	-2.93889100	0.50249400	2.24434600
H	-1.73511100	1.23635300	1.16174800
H	-1.62971700	-1.52572300	2.49011600
H	-0.57647300	-0.13093600	2.83161600
H	-4.03386400	1.99179900	1.08858600
H	-6.21469400	2.82566600	0.35965700
H	-7.58136600	1.54942500	-1.28908200
H	-6.70762300	-0.61823000	-2.16780800
H	-4.55066400	-1.49674300	-1.39729000
H	3.41121100	-3.75277200	-1.30029900

5-phenyl-4-(4-phenylpiperazine-1-carbonyl)-3*H*-1,2,3-triazole

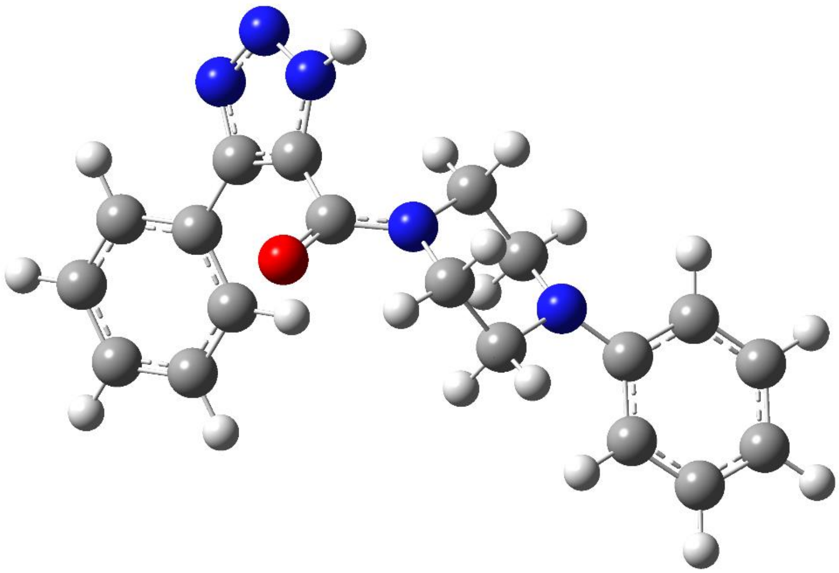

C	5.18712500	2.52440000	-0.45302000
C	4.22007900	3.45839400	-0.07184700
C	2.91299700	3.03414400	0.17943700
C	2.57375800	1.68736200	0.05374100
C	3.54148100	0.73992900	-0.31887600
C	4.85263200	1.17692700	-0.57512700
C	3.21334000	-0.68668200	-0.45811700
C	2.18580300	-1.44396600	0.09809500
N	2.39648900	-2.68861700	-0.40487800
N	3.47087700	-2.72870100	-1.20979500
N	3.95953400	-1.51857300	-1.25139200
C	1.18145900	-1.14068000	1.17094600
O	1.58771900	-0.73178600	2.26113900
N	-0.12992400	-1.37281000	0.89048700

C	-0.69560900	-1.65521800	-0.43097800
C	-1.73956200	-0.59649400	-0.79589400
N	-2.76972000	-0.49515900	0.24571700
C	-2.18149200	-0.15614000	1.54048900
C	-1.13964600	-1.20523700	1.93768400
C	-3.98141400	0.14290000	-0.11885200
C	-4.53586100	1.19682900	0.62977700
C	-5.76042400	1.76356400	0.26478600
C	-6.45245900	1.31045000	-0.85721600
C	-5.90525100	0.26672200	-1.61118000
C	-4.69426200	-0.31431400	-1.24664100
H	6.20619400	2.84471400	-0.65186000
H	4.48192900	4.50832000	0.02492400
H	2.15132200	3.75391800	0.46607800
H	1.54784000	1.37848900	0.22801500
H	5.60460400	0.45048600	-0.86549300
H	0.09401000	-1.65676700	-1.18252100
H	-1.16520900	-2.64682700	-0.41381600
H	-1.23266800	0.37448300	-0.93922500
H	-2.19703000	-0.87571600	-1.74585300
H	-2.96658900	-0.15178000	2.30125200
H	-1.71037500	0.84309400	1.53156000
H	-1.63623300	-2.17102400	2.09508800
H	-0.63126000	-0.91186000	2.85540300
H	-4.01259500	1.59172600	1.49310000
H	-6.16397100	2.57659200	0.86303800
H	-7.40128400	1.75716200	-1.13989000
H	-6.43265400	-0.11064400	-2.48356000
H	-4.30831600	-1.14864900	-1.82450400
H	1.88357700	-3.54174900	-0.21506600

Tautomers of **7b**
5-phenyl-4-(4-(3-chlorophenyl)piperazine-1-carbonyl)-1*H*-1,2,3-triazole

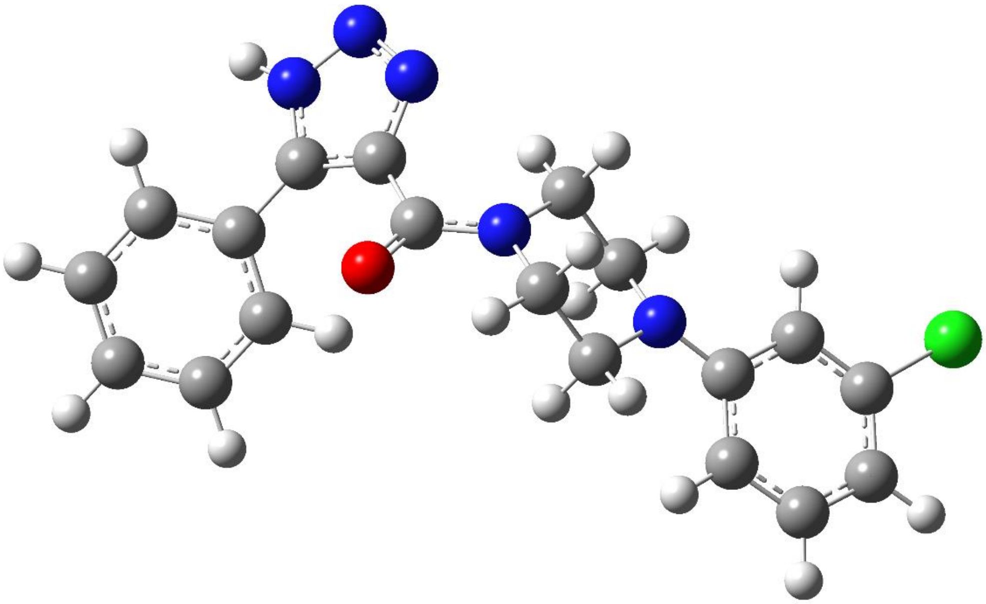

C	6.43911400	1.60455100	-0.89869400
C	5.68940800	2.77176700	-1.05818100
C	4.29505600	2.71768400	-0.97632700
C	3.65127100	1.50517200	-0.73949300
C	4.39801900	0.32629300	-0.57909200
C	5.79972600	0.38864600	-0.65899500
C	3.72509100	-0.95889600	-0.35252400
C	2.58526100	-1.35588000	0.34883600
N	2.43191300	-2.70707000	0.20201100
N	3.39018700	-3.17503200	-0.54958800
N	4.16662000	-2.12643200	-0.88551400
C	1.76081500	-0.52543700	1.27779500
O	2.32006700	0.33865100	1.96376400
N	0.42160000	-0.77766100	1.36550400
C	-0.38576700	-1.59069400	0.45553800
C	-1.45671300	-0.72694400	-0.21623900
N	-2.27678700	-0.02382300	0.78146000
C	-1.45755600	0.80098100	1.66994600
C	-0.38426500	-0.05434100	2.34848600
C	-3.51714700	0.49237600	0.35783700
C	-3.92935100	1.80816400	0.64628600
C	-5.19002300	2.26154800	0.25586100
C	-6.07504300	1.44211300	-0.44300600
C	-5.65539900	0.14306400	-0.72846300
C	-4.41471400	-0.34421000	-0.34127900
H	7.52328000	1.63887100	-0.95349600
H	6.18805000	3.71879000	-1.24360000
H	3.70612100	3.62148600	-1.10311500

H	2.56825100	1.46799900	-0.69075100
H	6.39381300	-0.50870800	-0.50976700
H	0.24615000	-2.04705900	-0.30253000
H	-0.86606700	-2.39371100	1.02946700
H	-0.96593100	-0.00240600	-0.88929500
H	-2.08676300	-1.37329600	-0.82833900
H	-2.09353500	1.22771100	2.44989700
H	-0.97638500	1.63339200	1.12710900
H	-0.86861400	-0.78745300	3.00654200
H	0.28031000	0.57272900	2.94118300
H	-3.26620100	2.49264800	1.16075100
H	-5.48073900	3.28210300	0.48908200
H	-7.05085500	1.79583200	-0.75525400
H	-4.15755900	-1.37497000	-0.55361100
H	4.94937600	-2.26956400	-1.51267700
Cl	-6.75321500	-0.93936600	-1.59874000

5-phenyl-4-(4-(3-chlorophenyl)piperazine-1-carbonyl)-2*H*-1,2,3-triazole

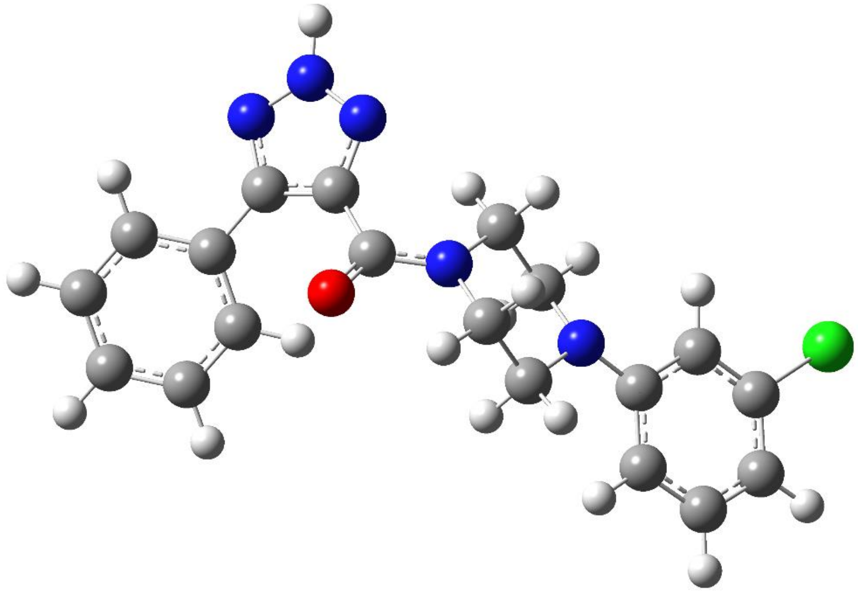

C	6.20395600	1.85629200	-1.11170100
C	5.38760100	2.98545700	-1.00523200
C	4.03264800	2.83178500	-0.70221000
C	3.49551600	1.56019600	-0.50389500
C	4.30944900	0.42049500	-0.60285400
C	5.67062400	0.58413400	-0.91208100
C	3.76108600	-0.93324500	-0.41589600
C	2.64828300	-1.40661400	0.33410000
N	2.55322600	-2.73349600	0.16789700
N	3.56090400	-3.01426200	-0.64270500
N	4.31174200	-1.99212600	-1.03134700
C	1.80815700	-0.67478300	1.33563200

O	2.37096400	0.06329800	2.15129800
N	0.46059900	-0.88036400	1.32873700
C	-0.31540600	-1.58985800	0.31137000
C	-1.38281500	-0.66466800	-0.27798000
N	-2.23131600	-0.09375600	0.77784400
C	-1.44066400	0.63946900	1.76601800
C	-0.36921000	-0.27222800	2.36925800
C	-3.47655300	0.44223100	0.39442100
C	-3.91910200	1.71084100	0.81697000
C	-5.18366500	2.17960000	0.45835600
C	-6.04218400	1.42347700	-0.33814300
C	-5.59241800	0.17098600	-0.75536600
C	-4.34724500	-0.33154100	-0.40370500
H	7.25915200	1.96580300	-1.34631100
H	5.80390000	3.97715200	-1.15868100
H	3.38872400	3.70342300	-0.62528900
H	2.43819000	1.45547100	-0.28512600
H	6.30640900	-0.29228700	-0.98834400
H	0.34042500	-1.94098000	-0.48246800
H	-0.79553000	-2.46276000	0.77248700
H	-0.88746000	0.13948700	-0.84969100
H	-1.99165800	-1.24047100	-0.97572000
H	-2.09640000	0.97136100	2.57504800
H	-0.96155900	1.53199900	1.32663200
H	-0.85506600	-1.07438600	2.93998300
H	0.28054600	0.29429300	3.03504800
H	-3.27682800	2.34798900	1.41259400
H	-5.49852900	3.16319900	0.79566500
H	-7.02097000	1.79015500	-0.62516700
Cl	-6.65644300	-0.83183000	-1.75334600
H	-4.06575300	-1.32844500	-0.72105800
H	3.74980000	-3.96041400	-0.94844300

5-phenyl-4-(4-(3-chlorophenyl)piperazine-1-carbonyl)-3*H*-1,2,3-triazole

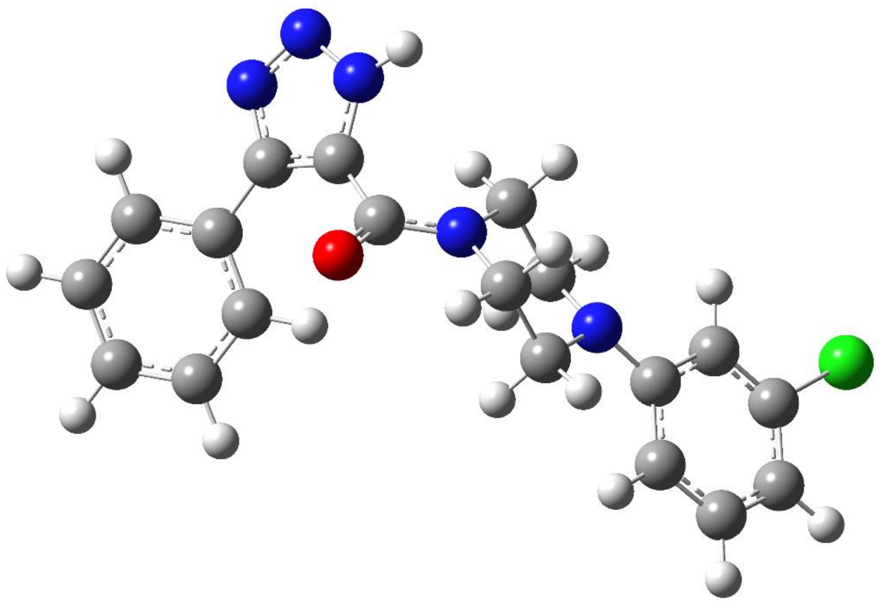

C	5.74671800	2.41070400	-0.98917500
C	4.86153700	3.38853600	-0.52763500
C	3.59212100	3.01330600	-0.08132300
C	3.20904700	1.67248300	-0.09356100
C	4.09518700	0.68156700	-0.54732100
C	5.36863300	1.06930700	-0.99905700
C	3.72059400	-0.74046300	-0.57546100
C	2.74524300	-1.44413700	0.12672200
N	2.87210800	-2.71595400	-0.33528800
N	3.84875700	-2.82169600	-1.25055000
N	4.35506800	-1.62820900	-1.40496700
C	1.86606900	-1.06872200	1.28270900
O	2.38900100	-0.60750600	2.29947300
N	0.52988200	-1.30039000	1.15804500
C	-0.17975600	-1.63177000	-0.07928300
C	-1.23560800	-0.56601200	-0.38443500
N	-2.14485200	-0.38444600	0.75549300
C	-1.41802600	-0.01097400	1.96892200
C	-0.35945700	-1.06668600	2.29720400
C	-3.38276000	0.24289800	0.50517400
C	-3.86852600	1.30312600	1.29401200
C	-5.12413100	1.85868100	1.04410400
C	-5.92921100	1.39992200	0.00281300
C	-5.43618400	0.35292200	-0.77571200
C	-4.19918200	-0.23269900	-0.54348400
H	6.73604800	2.69207000	-1.33941800
H	5.15713100	4.43391400	-0.51855800
H	2.89279200	3.76666600	0.27058700
H	2.21082800	1.40242600	0.23577300
H	6.05705700	0.30921200	-1.35363500

H	0.52304200	-1.68697100	-0.91047200
H	-0.66304200	-2.60992300	0.03682700
H	-0.72906400	0.38357800	-0.62979600
H	-1.79673100	-0.87984800	-1.26513700
H	-2.11776300	0.03584800	2.80700500
H	-0.93567400	0.97702100	1.86980000
H	-0.85436100	-2.01338700	2.54796500
H	0.24763500	-0.74719400	3.14343000
H	-3.26687300	1.71379100	2.09558700
H	-5.47372700	2.67778200	1.66628100
H	-6.90067200	1.83644100	-0.19872500
Cl	-6.43187500	-0.27361800	-2.09793900
H	-3.88349100	-1.07170000	-1.15207500
H	2.36615700	-3.54525600	-0.04666800

Tautomers of **7h**

5-phenyl-4-(4-(3,5-dimethylphenyl)piperazine-1-carbonyl)-1*H*-1,2,3-triazole

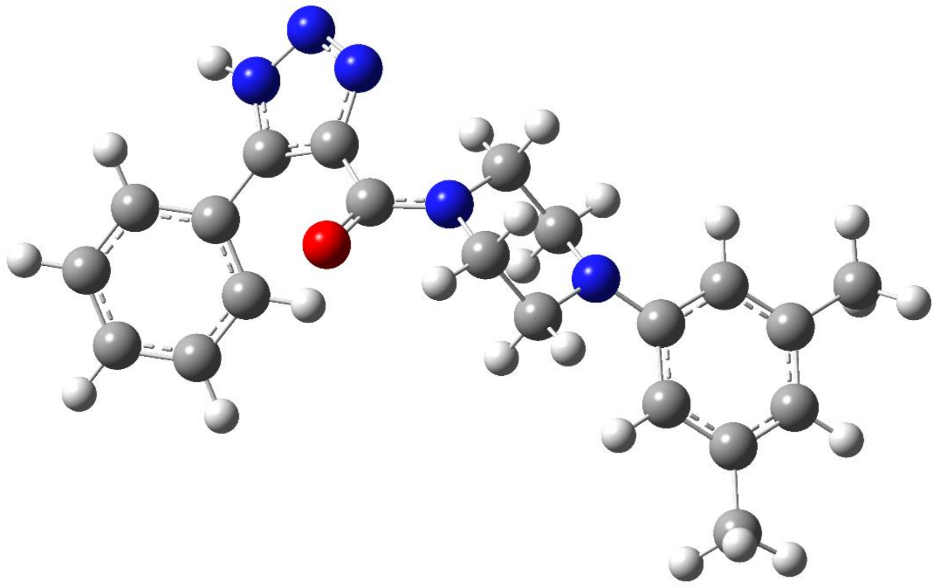

C	6.20311500	2.03950200	-0.74071200
C	5.33531000	3.12908300	-0.83964800
C	3.95462300	2.92548600	-0.76355900
C	3.44159600	1.64151300	-0.59266300
C	4.30768300	0.53988100	-0.49279600
C	5.69501600	0.75268100	-0.56657400
C	3.77257000	-0.81797700	-0.33689200
C	2.66426000	-1.36186900	0.31365600
N	2.65646400	-2.71369300	0.10653300
N	3.67756700	-3.04602200	-0.63508700
N	4.34768200	-1.90885600	-0.90454000
C	1.73239400	-0.67138400	1.25647400
O	2.18663300	0.17692600	2.03397700
N	0.41752700	-1.03621300	1.25079100
C	-0.26855800	-1.83549400	0.23410400
C	-1.38797000	-1.01665700	-0.41440000
N	-2.31415200	-0.49017600	0.59737300
C	-1.61200700	0.33731400	1.57696000
C	-0.49388800	-0.46763100	2.24420400
C	-3.57391700	-0.03727500	0.13129200
C	-4.07935100	1.23366600	0.44019700
C	-5.35523500	1.63283300	0.00916400
C	-6.12189900	0.75310700	-0.75470100
C	-5.63914600	-0.52438800	-1.08605600
C	-4.37859900	-0.90817000	-0.63425500
H	7.27757700	2.18953200	-0.79177100
H	5.73194800	4.13131500	-0.97401300
H	3.27408600	3.76819000	-0.84406700

H	2.36833500	1.48959900	-0.55079900
H	6.38057300	-0.08399800	-0.46443500
H	0.43798800	-2.16073400	-0.52603200
H	-0.69362600	-2.72791300	0.71158700
H	-0.93878200	-0.19167400	-0.99665800
H	-1.92790900	-1.65854000	-1.11203300
H	-2.31764900	0.65173800	2.35087300
H	-1.18280400	1.24655000	1.11834100
H	-0.93493100	-1.29151400	2.82033100
H	0.08496600	0.16694000	2.91394400
H	-3.48254000	1.93778000	1.01034800
C	-5.87580000	3.00520300	0.37417000
H	-7.10960100	1.05719900	-1.09513500
C	-6.47913200	-1.46582200	-1.91985900
H	-4.02197100	-1.91057500	-0.85415400
H	5.15592800	-1.94170100	-1.51438300
H	-6.82518500	3.21860600	-0.12672700
H	-5.16278100	3.79075000	0.09600200
H	-6.04125900	3.09398900	1.45558800
H	-6.08241400	-2.48570100	-1.89631600
H	-6.50869500	-1.14661600	-2.96999800
H	-7.51631500	-1.49552900	-1.56644300

5-phenyl-4-(4-(3,5-dimethylphenyl)piperazine-1-carbonyl)-2*H*-1,2,3-triazole

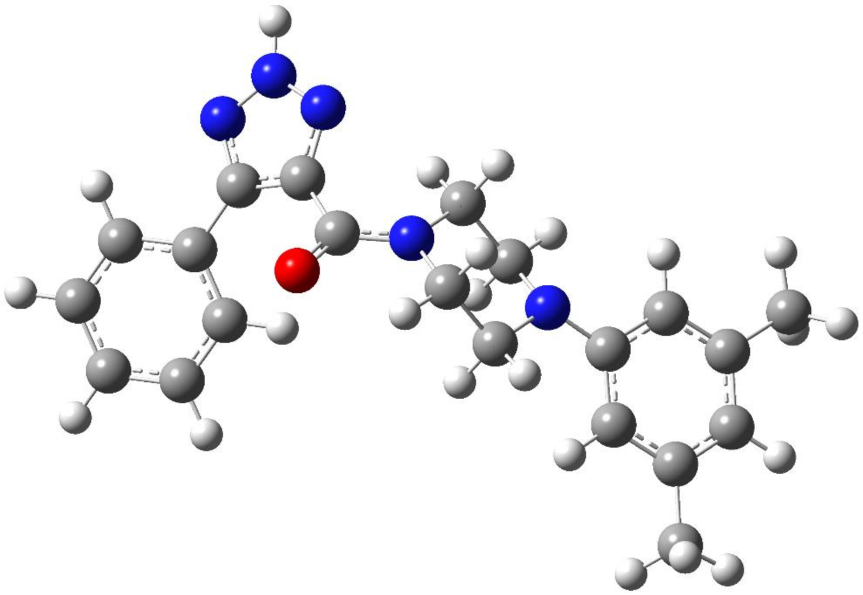

C	6.01636300	2.21318900	-0.92030400
C	5.09662500	3.26119400	-0.82763600
C	3.75095300	2.97906800	-0.58088800
C	3.32514900	1.66018100	-0.42517700
C	4.24323700	0.60136900	-0.51066000
C	5.59442900	0.89414600	-0.76284800

C	3.81465900	-0.80015800	-0.36847300
C	2.72002900	-1.38672900	0.32598200
N	2.75398100	-2.71332000	0.13615200
N	3.81592600	-2.88598300	-0.63480700
N	4.48521700	-1.79267400	-0.97565100
C	1.77578800	-0.75351500	1.30212400
O	2.23813500	0.00633900	2.16036500
N	0.45267400	-1.06857000	1.22372800
C	-0.21005500	-1.81529500	0.15327000
C	-1.33308600	-0.97535300	-0.46034400
N	-2.27582900	-0.51976300	0.57015400
C	-1.59402400	0.26421700	1.59823900
C	-0.47410800	-0.56061800	2.23645000
C	-3.53857600	-0.06015100	0.11781200
C	-4.06457000	1.18506200	0.48934800
C	-5.34256900	1.58883300	0.06820800
C	-6.08947000	0.74081400	-0.74874800
C	-5.58588200	-0.51088700	-1.14263800
C	-4.32383800	-0.90028300	-0.70061600
H	7.06525700	2.42282100	-1.11101100
H	5.42582000	4.28952700	-0.94806700
H	3.02743500	3.78689700	-0.51529300
H	2.27407100	1.45530200	-0.25172300
H	6.30993000	0.08056100	-0.82822300
H	0.51072900	-2.08197100	-0.61689600
H	-0.62743900	-2.74103200	0.57039800
H	-0.88964400	-0.11313000	-0.99063600
H	-1.85699000	-1.58401300	-1.19872700
H	-2.31083400	0.53031600	2.37982700
H	-1.17105600	1.20057700	1.19137600
H	-0.91054500	-1.41621000	2.76843100
H	0.09276200	0.04508600	2.94236200
H	-3.48269000	1.86662500	1.10086900
C	-5.88631900	2.93199200	0.50157200
H	-7.07807600	1.04934900	-1.08256300
C	-6.40964800	-1.42075800	-2.02617200
H	-3.95037600	-1.88411400	-0.97069800
H	4.10303700	-3.80509600	-0.94648300
H	-6.82567700	3.16622700	-0.00884100
H	-5.17523400	3.73934900	0.28894000
H	-6.07904500	2.95376500	1.58196200
H	-5.93969600	-2.40255900	-2.14048900
H	-6.53558600	-0.99278000	-3.02905500
H	-7.41552700	-1.57192400	-1.61642000

5-phenyl-4-(4-(3,5-dimethylphenyl)piperazine-1-carbonyl)-3*H*-1,2,3-triazole

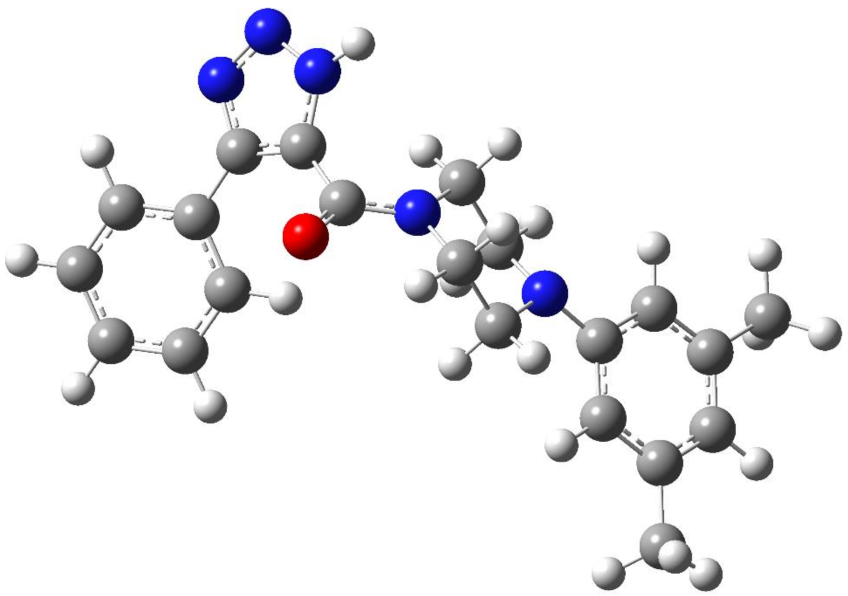

C	5.51427900	2.65476900	-0.94283400
C	4.50462500	3.56564900	-0.62083600
C	3.24694800	3.09351300	-0.23766300
C	2.99901200	1.72271000	-0.17438300
C	4.01044500	0.79950800	-0.48707200
C	5.27133900	1.28402700	-0.87604700
C	3.77795100	-0.65136200	-0.42925700
C	2.82873400	-1.39517600	0.26662900
N	3.10600600	-2.67803900	-0.08406200
N	4.14808000	-2.75275500	-0.92852000
N	4.54863000	-1.52933100	-1.14608800
C	1.84650200	-1.02345600	1.33920900
O	2.26895200	-0.48388100	2.36457000
N	0.53905000	-1.33719500	1.13173600
C	-0.05013100	-1.80060600	-0.12722300
C	-1.16030600	-0.84585800	-0.57391700
N	-2.16184300	-0.67547900	0.48584500
C	-1.55222100	-0.15132000	1.70600000
C	-0.44559500	-1.09299300	2.18827000
C	-3.41718800	-0.14466300	0.09099800
C	-3.99303900	0.97199000	0.71234300
C	-5.26208200	1.43964100	0.33231600
C	-5.94767200	0.78962600	-0.69350900
C	-5.39211800	-0.32802300	-1.33946500
C	-4.14060800	-0.78738700	-0.93584000
H	6.49561900	3.01191700	-1.24294500
H	4.69531600	4.63400800	-0.67089300
H	2.45219600	3.79332200	0.00530800
H	2.00878000	1.37389400	0.10135400
H	6.05672600	0.57633700	-1.12058300

H	0.71634000	-1.85099100	-0.90079300
H	-0.46470800	-2.80572800	0.02171300
H	-0.71088200	0.12585900	-0.84739400
H	-1.63129200	-1.25666400	-1.46790300
H	-2.31281600	-0.08937600	2.48886000
H	-1.13312500	0.86028900	1.55794700
H	-0.88639100	-2.05515500	2.47836600
H	0.07515400	-0.66688300	3.04503100
H	-3.45814200	1.50381900	1.49234600
C	-5.86206100	2.63843900	1.03269900
H	-6.92926300	1.14960300	-0.99512400
C	-6.14708800	-1.02033600	-2.45190700
H	-3.72780900	-1.67624700	-1.40485900
H	2.66185300	-3.53012200	0.23781500
H	-6.79911000	2.94820100	0.55997800
H	-5.17679600	3.49465300	1.01848500
H	-6.07550400	2.41888500	2.08664700
H	-5.68088400	-1.97338700	-2.72063700
H	-6.17793600	-0.39910700	-3.35652600
H	-7.18675200	-1.21864600	-2.16618700

Tautomers of **7j**

5-phenyl-4-(4-(3,5-dimethoxyphenyl)piperazine-1-carbonyl)-1*H*-1,2,3-triazole

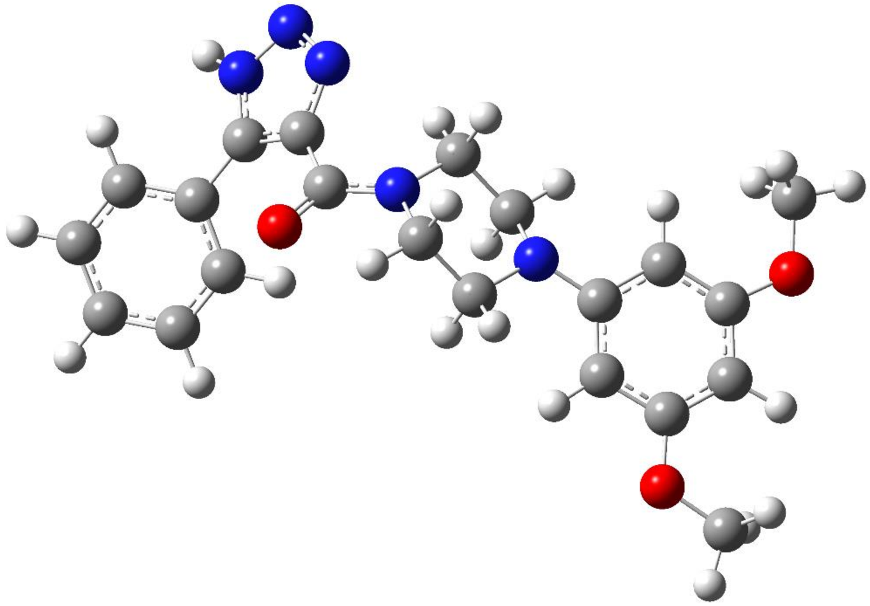

C	6.77762300	1.94172300	-0.90613200
C	5.93463000	3.03260600	-1.12784300
C	4.54854500	2.86205300	-1.06787200
C	4.00576600	1.60931600	-0.79078400
C	4.84675700	0.50653500	-0.56790000
C	6.23936100	0.68598300	-0.62634600
C	4.28218700	-0.82214000	-0.29959900
C	3.16819000	-1.28971100	0.40024400
N	3.13628400	-2.65382500	0.30641500
N	4.14518100	-3.06507200	-0.41138700
N	4.83340000	-1.96628000	-0.77810000
C	2.26008600	-0.49492900	1.28120200
O	2.73218300	0.45200800	1.92269200
N	0.94888100	-0.86260100	1.37608600
C	0.22838000	-1.80434000	0.51720300
C	-0.92810700	-1.09714100	-0.19362200
N	-1.81486000	-0.42933900	0.76979900
C	-1.07894600	0.53482400	1.58631300
C	0.07319400	-0.16065200	2.31531700
C	-3.09860200	-0.05828200	0.30500100
C	-3.59856100	1.23687300	0.46367300
C	-4.90162100	1.54798300	0.03483700
C	-5.70752600	0.58806700	-0.56897200
C	-5.19441500	-0.71246100	-0.73009300
C	-3.91414900	-1.04560300	-0.29802100
H	7.85582200	2.06703600	-0.94340200
H	6.35452600	4.01070900	-1.34439700
H	3.88742800	3.70590700	-1.24286200

H	2.92917500	1.48106800	-0.75816500
H	6.90442000	-0.15027300	-0.42910400
H	0.90485500	-2.23485300	-0.21710700
H	-0.16503400	-2.61943700	1.13860200
H	-0.51625600	-0.36589600	-0.91222800
H	-1.48983200	-1.83863600	-0.76339300
H	-1.75470400	0.95766300	2.33454300
H	-0.67902600	1.36702500	0.97967100
H	-0.33559300	-0.89345300	3.02338000
H	0.67197500	0.56690100	2.86152900
H	-3.00513000	2.03449200	0.89199800
O	-5.28037200	2.84400900	0.24354500
H	-6.71187900	0.79675300	-0.91422600
O	-6.05758800	-1.59515200	-1.31693100
H	-3.55106500	-2.06130500	-0.37866200
C	-6.57629100	3.24514100	-0.19126300
C	-5.62140900	-2.93510500	-1.52327300
H	-6.66223700	4.30295000	0.06218000
H	-7.36375200	2.68190300	0.32410800
H	-6.69169800	3.11900100	-1.27467600
H	-6.45103400	-3.44637400	-2.01418500
H	-5.39806100	-3.43495300	-0.57278400
H	-4.73582400	-2.97358200	-2.16924800
H	5.63637900	-2.06420300	-1.38813600

5-phenyl-4-(4-(3,5-dimethoxyphenyl)piperazine-1-carbonyl)-2*H*-1,2,3-triazole

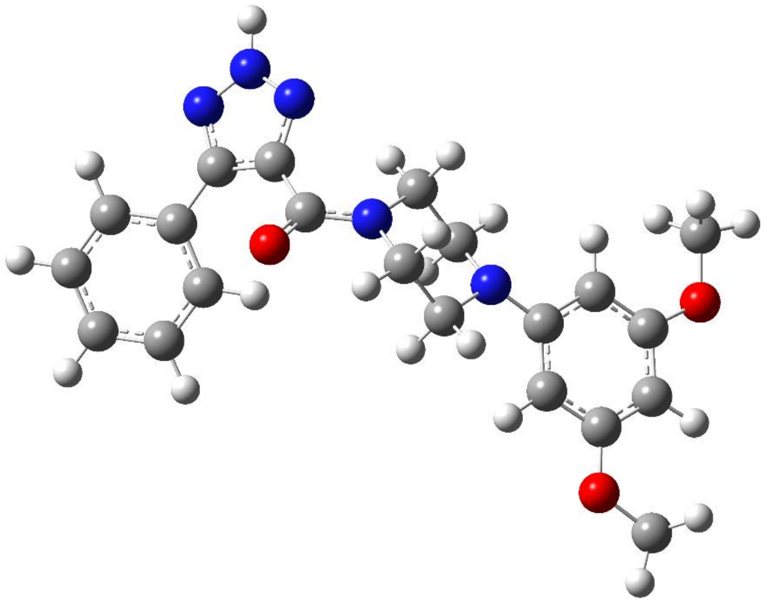

C	6.37239900	2.24323700	-1.27669000
C	5.41521600	3.26135100	-1.26372200
C	4.09112700	2.96026000	-0.93595000
C	3.72421600	1.65226800	-0.62069300

C	4.68048200	0.62422200	-0.62518300
C	6.00947600	0.93541400	-0.95973500
C	4.31139600	-0.76661800	-0.31381500
C	3.26242200	-1.30739500	0.48041100
N	3.33672100	-2.64492900	0.43569300
N	4.37870900	-2.86787200	-0.35008500
N	4.99682800	-1.79820200	-0.83278700
C	2.32953800	-0.60230700	1.41792900
O	2.79688500	0.23905400	2.19307200
N	1.01116400	-0.94464400	1.39906100
C	0.34059600	-1.79644300	0.41520600
C	-0.80836600	-1.03348000	-0.24835100
N	-1.73843000	-0.50101500	0.75664400
C	-1.05101600	0.38504800	1.69503000
C	0.09638700	-0.36035200	2.38076000
C	-3.02107800	-0.11548000	0.29978800
C	-3.56145200	1.14170800	0.58143900
C	-4.86238800	1.46254300	0.15298600
C	-5.62565400	0.55135600	-0.57000200
C	-5.07179800	-0.71086300	-0.85383200
C	-3.79297900	-1.05431200	-0.42539500
H	7.40484200	2.46812000	-1.52966500
H	5.69878100	4.28110300	-1.50873400
H	3.33855500	3.74380400	-0.93155000
H	2.68832100	1.42964700	-0.38675300
H	6.75426000	0.14581400	-0.96338800
H	1.05072000	-2.12036700	-0.34304700
H	-0.05211400	-2.68688600	0.92311400
H	-0.39079500	-0.21474400	-0.86126500
H	-1.33511600	-1.71346300	-0.91897200
H	-1.75763400	0.70821100	2.46393300
H	-0.65537300	1.28702000	1.19471000
H	-0.31404200	-1.16981500	2.99845300
H	0.66627900	0.31713900	3.01540800
H	-3.00154000	1.90419300	1.10771100
O	-5.28329900	2.71860500	0.48697900
H	-6.62691500	0.76933100	-0.91846700
O	-5.89525200	-1.54852100	-1.55272100
H	-3.39857600	-2.04590900	-0.60256400
C	-6.57946000	3.12973900	0.06221800
C	-5.41539100	-2.84670000	-1.88878400
H	-6.70273200	4.15119000	0.42555800
H	-7.36222900	2.49311100	0.49215100
H	-6.66444900	3.11878300	-1.03123700
H	-6.21800300	-3.32436000	-2.45296400
H	-5.19971500	-3.43930000	-0.99128400

H	-4.51423800	-2.79209300	-2.51159400
H	4.68805000	-3.80617600	-0.56955400

5-phenyl-4-(4-(3,5-dimethoxyphenyl)piperazine-1-carbonyl)-3*H*-1,2,3-triazole

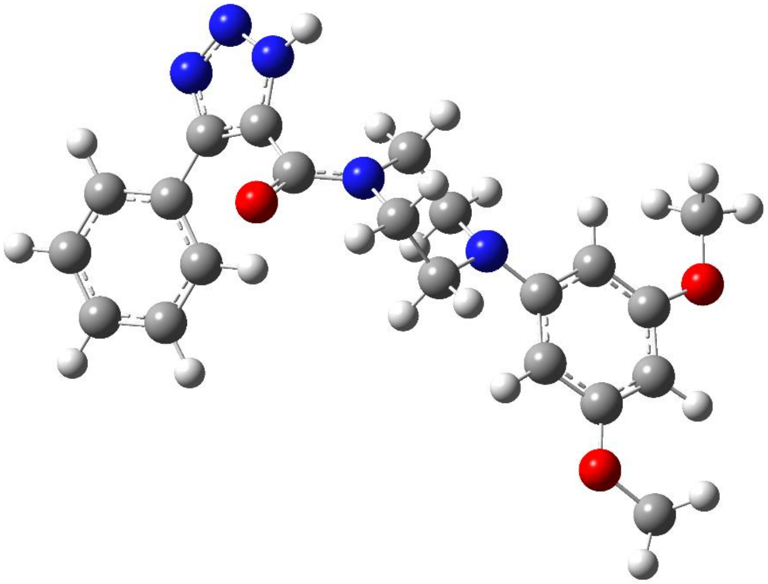

C	5.97786600	2.49091200	-1.47478000
C	4.97052900	3.44494800	-1.30799700
C	3.72109300	3.05046700	-0.82342500
C	3.47905800	1.71442700	-0.50559300
C	4.48833500	0.74991200	-0.66120500
C	5.74067900	1.15540400	-1.15426100
C	4.26315900	-0.66613500	-0.33366000
C	3.33249100	-1.27182000	0.50683600
N	3.61035000	-2.59689100	0.39088500
N	4.63507200	-2.82277200	-0.44727300
N	5.02374300	-1.65902800	-0.89398000
C	2.37089700	-0.70827300	1.51124600
O	2.80436800	0.05090400	2.38109700
N	1.06946600	-1.09781900	1.42769900
C	0.45041700	-1.82177200	0.31488800
C	-0.68992600	-0.99562100	-0.28630300
N	-1.66171000	-0.61316800	0.74647900
C	-1.02404200	0.15533600	1.81433300
C	0.11102000	-0.65584300	2.44312400
C	-2.93745900	-0.19178000	0.29948200
C	-3.51202500	1.00782200	0.72634400
C	-4.80551600	1.36079300	0.30062800
C	-5.52572400	0.54162800	-0.56298300
C	-4.93659200	-0.66164800	-0.99349100
C	-3.66547600	-1.03847600	-0.56963700
H	6.95285000	2.78745500	-1.85174700

H	5.15657600	4.48612200	-1.55625800
H	2.92804500	3.78264400	-0.69924100
H	2.49558000	1.42193800	-0.15182900
H	6.52448200	0.41535300	-1.27847900
H	1.19226500	-2.02596700	-0.45712400
H	0.05854300	-2.77712900	0.68607300
H	-0.26723000	-0.09909600	-0.77385900
H	-1.17955800	-1.59165300	-1.05727400
H	-1.76220000	0.36828200	2.59173700
H	-0.62659500	1.11858000	1.44791300
H	-0.30342300	-1.54440700	2.93592800
H	0.64715500	-0.05778600	3.17904500
H	-2.98516200	1.70326900	1.36699500
O	-5.26362200	2.55313300	0.78464300
H	-6.51967900	0.78754100	-0.91379300
O	-5.71827000	-1.41090700	-1.82715600
H	-3.24531300	-1.99138700	-0.86253500
C	-6.55625700	2.99276800	0.37774000
C	-5.19645700	-2.63937500	-2.32431100
H	-6.71288600	3.95446100	0.86880000
H	-7.33599300	2.28999000	0.69540400
H	-6.60980500	3.12529200	-0.70967000
H	-5.96687700	-3.04640100	-2.98109000
H	-4.99942300	-3.34976900	-1.51209800
H	-4.27505900	-2.47994600	-2.89740200
H	3.17903900	-3.37671800	0.87330000

Tautomers of **7k**

5-(2-fluorophenyl)-4-(4-(3,5-dimethylphenyl)piperazine-1-carbonyl)-1*H*-1,2,3-triazole

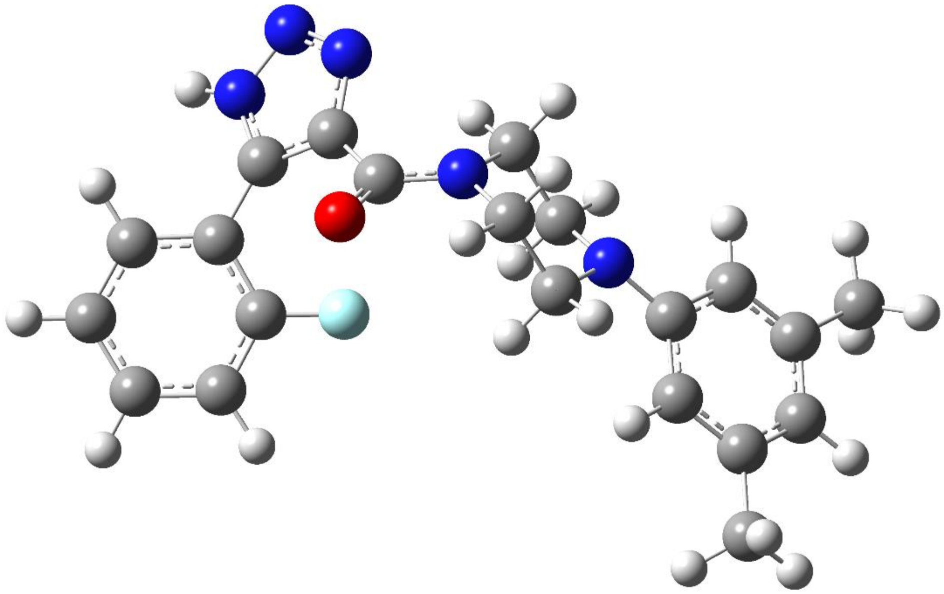

C	5.60917900	2.43024900	-0.55788100
C	4.59089500	3.38672300	-0.56171800
C	3.25388700	2.98693900	-0.52441100
C	2.96115300	1.63196800	-0.48453800
C	3.95408800	0.64366000	-0.47412800
C	5.29200000	1.07403300	-0.51295100
C	3.60773900	-0.77922600	-0.44157600
C	2.63769400	-1.51908600	0.22880400
N	2.77505900	-2.83379800	-0.11467300
N	3.76450400	-2.95902400	-0.95931500
N	4.26545300	-1.72580200	-1.15917900
C	1.71174400	-1.01920200	1.29080700
O	2.15465000	-0.24966600	2.15073900
N	0.42392900	-1.46627900	1.28626400
C	-0.25798100	-2.11447700	0.16400700
C	-1.30811100	-1.17013100	-0.42787500
N	-2.24977300	-0.71808900	0.60681300
C	-1.55453400	-0.05313600	1.70791100
C	-0.50419500	-0.99017000	2.31292100
C	-3.46707300	-0.14890000	0.15627300
C	-3.92663000	1.09841800	0.60317500
C	-5.16470900	1.61060100	0.18132800
C	-5.93835400	0.87146300	-0.71287100
C	-5.50117300	-0.37873600	-1.18348900
C	-4.27852400	-0.87729700	-0.74030800
H	6.64949600	2.73864200	-0.58103300
H	4.83265200	4.44466500	-0.59202500
H	2.44031100	3.70437400	-0.53293200

F	1.66085300	1.25564500	-0.47202300
H	6.08724600	0.33493800	-0.48365400
H	0.46266700	-2.39821600	-0.59995300
H	-0.74472000	-3.02626100	0.53189600
H	-0.79172400	-0.30901800	-0.88901300
H	-1.84936900	-1.69508300	-1.21639300
H	-2.27947900	0.20285200	2.48554300
H	-1.06090200	0.88004100	1.38166200
H	-1.00256600	-1.86020500	2.75782500
H	0.06992400	-0.47406200	3.08138400
H	-3.32213900	1.69755800	1.27609300
C	-5.63902000	2.95042000	0.69868800
H	-6.89580700	1.26567400	-1.04774100
C	-6.35484100	-1.17036500	-2.14862900
H	-3.95880000	-1.86111400	-1.07203900
H	5.02004600	-1.59744900	-1.82320400
H	-6.52482000	3.29617800	0.15695700
H	-4.86058400	3.71634500	0.60010200
H	-5.89974900	2.89645500	1.76372600
H	-5.92744200	-2.15850100	-2.34526200
H	-6.45223500	-0.65085500	-3.11050100
H	-7.37048600	-1.31196500	-1.75973800

5-(2-fluorophenyl)-4-(4-(3,5-dimethylphenyl)piperazine-1-carbonyl)-2*H*-1,2,3-triazole

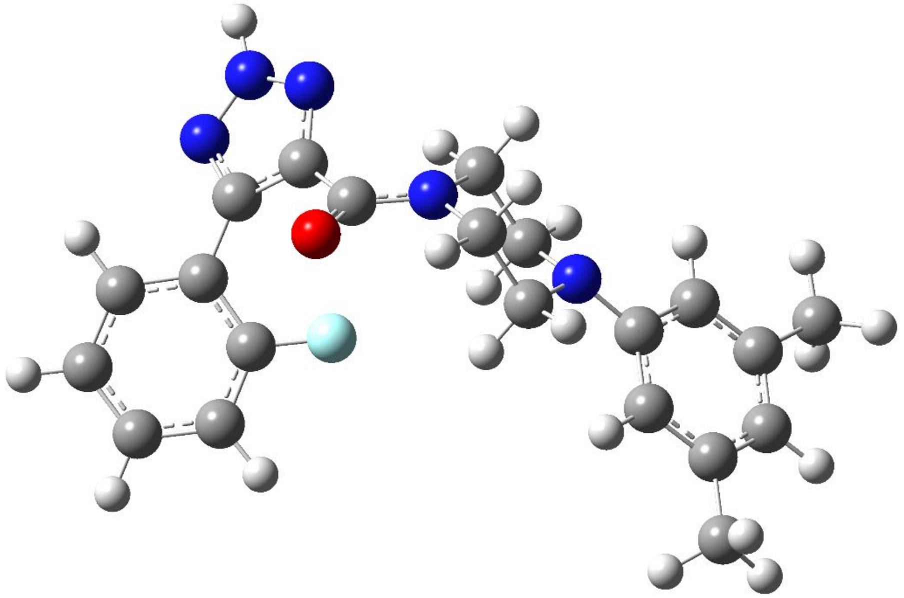

C	5.35698800	2.65931500	-0.66421600
C	4.27723400	3.53646600	-0.52951400
C	2.98377000	3.03287600	-0.38474400
C	2.79846600	1.65780000	-0.37501700
C	3.85364200	0.74651000	-0.49255900
C	5.14416400	1.28248200	-0.64528900

C	3.63969400	-0.70768300	-0.49297500
C	2.70853000	-1.51985600	0.20639300
N	2.92337100	-2.79933800	-0.12490700
N	3.93408000	-2.72802300	-0.98000500
N	4.40240800	-1.51640200	-1.24555500
C	1.76696500	-1.11216000	1.29853000
O	2.21109700	-0.42839900	2.22610100
N	0.47811600	-1.54509800	1.23693300
C	-0.18143500	-2.12287200	0.06407900
C	-1.23432800	-1.15347100	-0.48007100
N	-2.19224700	-0.77728000	0.57009200
C	-1.51602800	-0.17637500	1.71902900
C	-0.46677900	-1.14164000	2.27969900
C	-3.41016400	-0.19305700	0.14087700
C	-3.88858200	1.01916900	0.65971500
C	-5.12620700	1.54504300	0.25480800
C	-5.87994000	0.85770800	-0.69636800
C	-5.42352800	-0.35532300	-1.23943100
C	-4.20205600	-0.87026400	-0.81109900
H	6.36462800	3.04712200	-0.77733000
H	4.43696500	4.61038500	-0.53759100
H	2.12134100	3.68412700	-0.28961200
F	1.52965000	1.18674300	-0.26667000
H	5.98023600	0.59726900	-0.74114700
H	0.55331700	-2.34572300	-0.70722200
H	-0.66171500	-3.06290700	0.36281300
H	-0.72272600	-0.26053900	-0.88035100
H	-1.76071000	-1.63470900	-1.30555900
H	-2.25274100	0.02597600	2.50134100
H	-1.02526000	0.77823800	1.45737600
H	-0.96331800	-2.04115700	2.66376300
H	0.09402000	-0.67023600	3.08587900
H	-3.29859500	1.58049300	1.37651200
C	-5.62590400	2.84039000	0.85495600
H	-6.83649100	1.26377700	-1.01945300
C	-6.25302200	-1.08948700	-2.26904400
H	-3.86762100	-1.82735300	-1.20134500
H	4.32870400	-3.55622700	-1.40742300
H	-6.45438500	3.25620100	0.27303200
H	-4.83103400	3.59372700	0.90201700
H	-5.98510200	2.69017700	1.88165800
H	-5.83098300	-2.07309900	-2.49743500
H	-6.30990600	-0.52488200	-3.20862900
H	-7.28323600	-1.23459700	-1.92251600

5-(2-fluorophenyl)-4-(4-(3,5-dimethylphenyl)piperazine-1-carbonyl)-3*H*-1,2,3-triazole

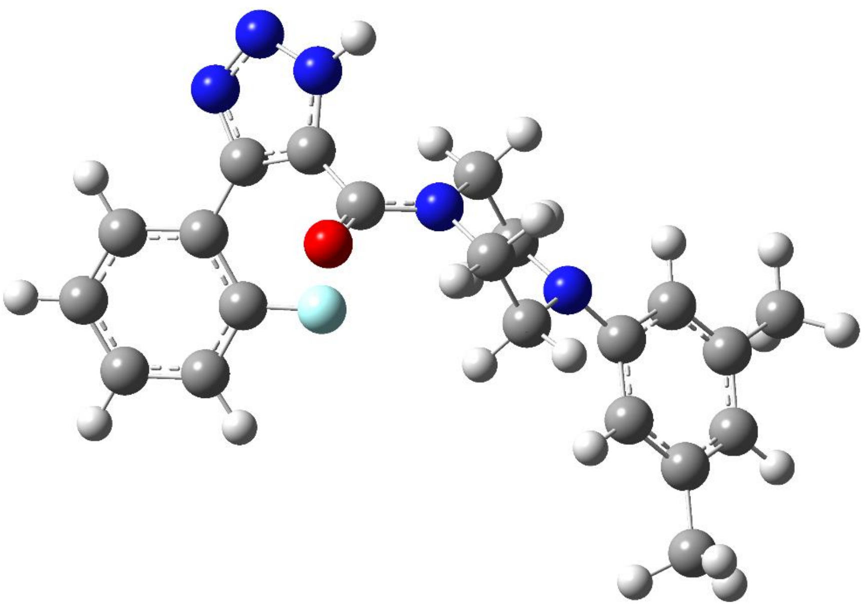

C	5.20911300	2.83050000	-0.70256200
C	4.11776300	3.65851800	-0.42472800
C	2.86864500	3.09599300	-0.15968800
C	2.74042600	1.71463600	-0.17602600
C	3.80865100	0.84957900	-0.43678200
C	5.05349600	1.44634800	-0.70786600
C	3.66641000	-0.61142200	-0.47039900
C	2.78262200	-1.46984200	0.17600700
N	3.16359600	-2.70067000	-0.25190000
N	4.20546900	-2.63855900	-1.09874600
N	4.50573500	-1.37666800	-1.23853900
C	1.78286800	-1.25386700	1.27571900
O	2.17833400	-0.78614500	2.34487900
N	0.50380800	-1.65116600	1.04286700
C	-0.07101000	-1.95256900	-0.27097500
C	-1.14718500	-0.92336600	-0.62865300
N	-2.16672000	-0.84521900	0.42698800
C	-1.57035400	-0.49028500	1.71410000
C	-0.50138000	-1.51734200	2.09918800
C	-3.39760800	-0.23435800	0.07613700
C	-3.96032600	0.80926500	0.82448400
C	-5.20861100	1.35565800	0.48414300
C	-5.88626500	0.86225100	-0.63064200
C	-5.34361900	-0.17794300	-1.40408600
C	-4.11316800	-0.71960500	-1.03907800
H	6.18304100	3.26293500	-0.91027000
H	4.23395600	4.73796400	-0.41450900
H	1.99629500	3.70598900	0.04983000
F	1.50902500	1.18808900	0.05821100
H	5.89830500	0.79980100	-0.92026300

H	0.70946600	-1.94036200	-1.03195500
H	-0.51243200	-2.95617400	-0.24002000
H	-0.66568900	0.05744100	-0.78207600
H	-1.61075000	-1.21979400	-1.57048900
H	-2.34708600	-0.49905400	2.48341400
H	-1.11765800	0.51720900	1.69752500
H	-0.97221200	-2.49635500	2.25065200
H	0.00523700	-1.21816900	3.01612200
H	-3.42913800	1.22167700	1.67575400
C	-5.79956500	2.46368400	1.32700300
H	-6.85095900	1.28528400	-0.90368400
C	-6.08993100	-0.70017800	-2.61124500
H	-3.71197400	-1.55170000	-1.61088700
H	2.78389700	-3.60320700	0.00963200
H	-6.65869500	2.92710300	0.83206300
H	-5.06197500	3.24845600	1.53221700
H	-6.14094600	2.08466000	2.29919600
H	-5.63661000	-1.61833300	-2.99774600
H	-6.09278900	0.03665900	-3.42502500
H	-7.13822800	-0.91364000	-2.37140900

Tautomers of **7l**

5-(2-fluorophenyl)-4-(4-(3,5-dimethoxyphenyl)piperazine-1-carbonyl)-1*H*-1,2,3-triazole

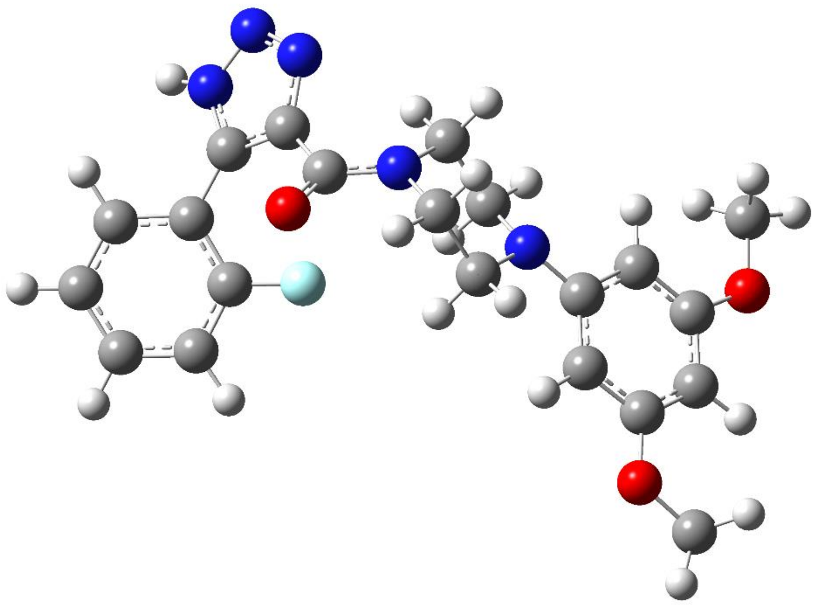

C	5.82575200	2.57957100	-1.00971200
C	4.74632700	3.46566100	-1.04990500
C	3.44514400	2.99268300	-0.87174900
C	3.24920400	1.63679300	-0.65685400
C	4.30559700	0.71799000	-0.60427500
C	5.60567400	1.22172500	-0.78772300
C	4.06133600	-0.71049800	-0.39298200
C	3.17228200	-1.43235000	0.39827500
N	3.38644900	-2.76559100	0.19357600
N	4.34901900	-2.91993700	-0.67715200
N	4.75478500	-1.68723000	-1.03282400
C	2.25462200	-0.88558700	1.44448300
O	2.68195500	-0.02556400	2.22202700
N	0.99221600	-1.39554300	1.51959400
C	0.30929100	-2.14856200	0.46564600
C	-0.77461500	-1.28152600	-0.18114900
N	-1.71190200	-0.76858000	0.82995200
C	-1.01796400	-0.00946300	1.87084700
C	0.06680200	-0.87140300	2.52426700
C	-2.94613600	-0.26210400	0.36039300
C	-3.43121500	0.98847800	0.75385900
C	-4.68955100	1.43050200	0.30732700
C	-5.46508900	0.65037200	-0.54434800
C	-4.96728900	-0.60457600	-0.94116500
C	-3.73267200	-1.06867800	-0.49713300
H	6.83888700	2.94527900	-1.14254800
H	4.91267100	4.52516900	-1.21811400
H	2.58521000	3.65306800	-0.90431300

F	1.98013800	1.18769500	-0.51052800
H	6.45025500	0.54137100	-0.73190600
H	1.02412300	-2.47415300	-0.28724800
H	-0.14619000	-3.03980000	0.91464900
H	-0.28898300	-0.44807400	-0.71862300
H	-1.31260400	-1.88221700	-0.91546700
H	-1.73842800	0.28117900	2.63990600
H	-0.55586900	0.91008900	1.46955800
H	-0.39887700	-1.72022000	3.03966300
H	0.63654700	-0.28389600	3.24313400
H	-2.85868400	1.65727700	1.38356500
O	-5.05678400	2.66673700	0.75912900
H	-6.43389700	0.96454600	-0.91058400
O	-5.79810000	-1.31054300	-1.76576100
H	-3.38679300	-2.05750200	-0.76655200
C	-6.30444300	3.19911200	0.32417200
C	-5.36910900	-2.58685000	-2.22978500
H	-6.38886300	4.18181200	0.79087700
H	-7.14275300	2.56961000	0.64648500
H	-6.33385900	3.30955100	-0.76666200
H	-6.16264900	-2.94755800	-2.88617700
H	-5.23594900	-3.29179300	-1.39998600
H	-4.43258300	-2.51299100	-2.79574600
H	5.47381400	-1.58461700	-1.73928200

5-(2-fluorophenyl)-4-(4-(3,5-dimethoxyphenyl)piperazine-1-carbonyl)-2*H*-1,2,3-triazole

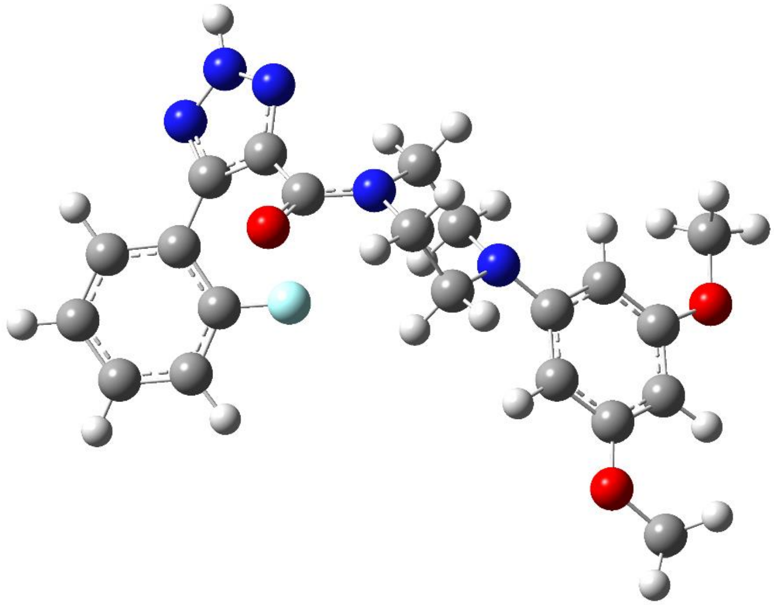

C	5.65744300	2.70555800	-1.10335400
C	4.54275400	3.54714900	-1.05282500
C	3.27724900	3.01421200	-0.80352400
C	3.15490400	1.64619100	-0.60663800

C	4.24689200	0.77194200	-0.63533400
C	5.50773600	1.33580400	-0.89658000
C	4.09841900	-0.67711900	-0.43903600
C	3.22541500	-1.42699100	0.39213100
N	3.48477100	-2.72939100	0.22060600
N	4.46518400	-2.73055000	-0.67198800
N	4.87243500	-1.54646600	-1.10783100
C	2.29966900	-0.91874400	1.45518500
O	2.74105300	-0.10830700	2.27556400
N	1.02834200	-1.40453200	1.49035500
C	0.35688400	-2.13190800	0.41148900
C	-0.73494100	-1.25946000	-0.21435900
N	-1.67773100	-0.78259600	0.80920700
C	-0.99070800	-0.03924300	1.86566700
C	0.09701800	-0.90827100	2.50428400
C	-2.91845100	-0.28037400	0.35175100
C	-3.41478800	0.95849200	0.76699100
C	-4.67911300	1.39451900	0.33140600
C	-5.44906000	0.62002300	-0.53047000
C	-4.93965200	-0.62310400	-0.94922400
C	-3.69885800	-1.08126900	-0.51638500
H	6.64336200	3.11641700	-1.29747100
H	4.65352500	4.61608700	-1.20768000
H	2.38896600	3.63602900	-0.76871300
F	1.91180300	1.14114100	-0.39648000
H	6.37128500	0.67925000	-0.92673900
H	1.07825200	-2.42444300	-0.34908200
H	-0.08946100	-3.04207400	0.83079600
H	-0.25838700	-0.40795200	-0.73033600
H	-1.26627000	-1.84785800	-0.96339100
H	-1.71404200	0.23216300	2.63899200
H	-0.53272300	0.89011400	1.48310100
H	-0.36488100	-1.77034400	3.00084400
H	0.66229900	-0.33366600	3.23705700
H	-2.84693400	1.62263700	1.40589600
O	-5.05805200	2.61915400	0.80439500
H	-6.42245800	0.92971700	-0.88827400
O	-5.76579600	-1.32443600	-1.78233600
H	-3.34288600	-2.06189500	-0.80227000
C	-6.31360600	3.14425400	0.38345100
C	-5.32591200	-2.58963300	-2.26616600
H	-6.40706900	4.11823300	0.86641100
H	-7.14319300	2.50002300	0.69914500
H	-6.34914800	3.27200500	-0.70529800
H	-6.11813900	-2.94868200	-2.92504100
H	-5.18297400	-3.30501200	-1.44699200

H	-4.39213200	-2.49841900	-2.83413400
H	4.88236700	-3.59131000	-1.00269300

5-(2-fluorophenyl)-4-(4-(3,5-dimethoxyphenyl)piperazine-1-carbonyl)-3*H*-1,2,3-triazole

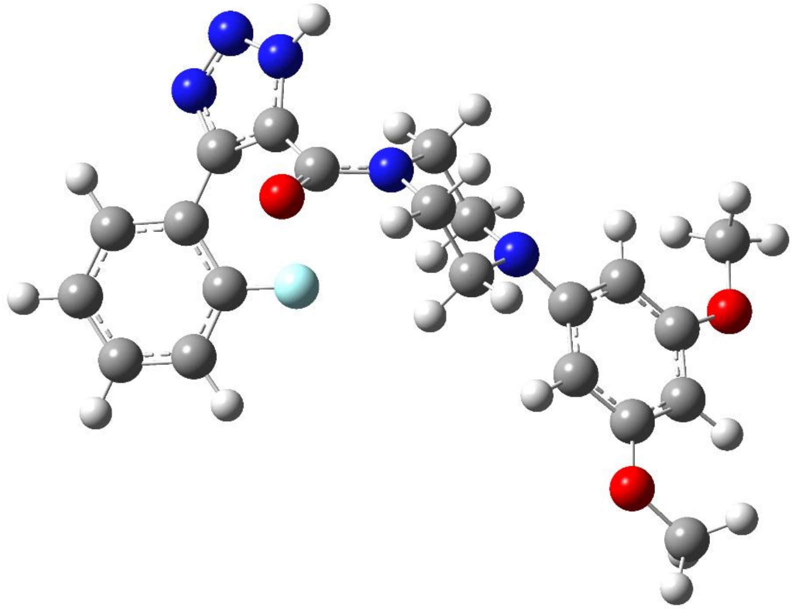

C	5.53551100	2.76951400	-1.28034600
C	4.42292100	3.60206300	-1.12969900
C	3.19608600	3.05962700	-0.74522200
C	3.11112700	1.69388500	-0.51645400
C	4.20263400	0.82755700	-0.64159300
C	5.42360600	1.40213800	-1.03899300
C	4.10754700	-0.61901500	-0.40748200
C	3.28120200	-1.36511600	0.42684100
N	3.68531200	-2.64365300	0.21519100
N	4.68824300	-2.71246700	-0.67732300
N	4.93955500	-1.49080400	-1.06085200
C	2.32030000	-0.95820600	1.50610300
O	2.73988200	-0.25744200	2.42880900
N	1.04803100	-1.43171500	1.42735900
C	0.43147200	-2.03739000	0.24440100
C	-0.67334000	-1.12971900	-0.30415100
N	-1.65491700	-0.81035900	0.74281600
C	-1.01877700	-0.16616600	1.89125300
C	0.07725600	-1.07040500	2.46277400
C	-2.90719000	-0.30922700	0.31456400
C	-3.45974400	0.85819600	0.84788200
C	-4.73412400	1.28793200	0.43546400
C	-5.45682400	0.58010500	-0.51966000
C	-4.88998300	-0.59031000	-1.05734100
C	-3.63881700	-1.04318600	-0.64927400
H	6.49220300	3.18611200	-1.58010700

H	4.50516200	4.66942700	-1.31074900
H	2.30823900	3.67205600	-0.62849100
F	1.90026200	1.18193400	-0.16973200
H	6.28584500	0.75312100	-1.14968100
H	1.18279500	-2.20066900	-0.52817100
H	0.00832200	-3.00850400	0.52855100
H	-0.21355000	-0.20937500	-0.70159100
H	-1.16505700	-1.64217800	-1.13178800
H	-1.76848300	-0.00213600	2.66956400
H	-0.58142400	0.81224200	1.62528600
H	-0.37205300	-1.99268600	2.85046500
H	0.60950500	-0.56543400	3.26807300
H	-2.93003800	1.47203400	1.56519100
O	-5.17162200	2.43806400	1.02885800
H	-6.43650500	0.88719100	-0.86239500
O	-5.67283400	-1.23098500	-1.97634400
H	-3.23818600	-1.97375700	-1.02820000
C	-6.44590600	2.94905000	0.64866600
C	-5.17478800	-2.42121800	-2.57948000
H	-6.58934900	3.85893100	1.23358200
H	-7.24743700	2.23707400	0.87986600
H	-6.47636700	3.19374300	-0.41998500
H	-5.94204200	-2.74059400	-3.28655900
H	-5.01513100	-3.21117600	-1.83542500
H	-4.23732400	-2.23561000	-3.11761100
H	3.35053200	-3.49197500	0.65731700

Tautomers of **7n**

5-(4-fluorophenyl)-4-(4-(3,5-dimethylphenyl)piperazine-1-carbonyl)-1*H*-1,2,3-triazole

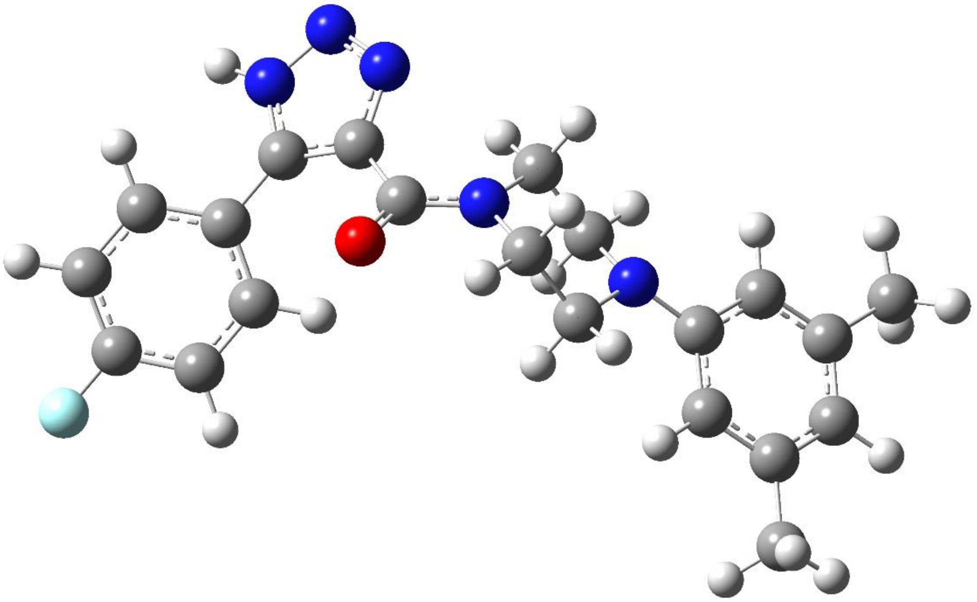

C	6.20245400	1.36078700	-0.49944100
C	5.43485700	2.51662100	-0.57788400
C	4.04413400	2.48544100	-0.56138800
C	3.40386100	1.25359400	-0.46596400
C	4.14623800	0.06306400	-0.38500300
C	5.55012400	0.13371100	-0.40092700
C	3.46934200	-1.23613300	-0.30537700
C	2.29129700	-1.68973800	0.29077000
N	2.15736100	-3.02544900	0.02854100
N	3.16302300	-3.43029200	-0.69690600
N	3.95060900	-2.35635100	-0.90193400
C	1.40500000	-0.93944500	1.23129200
O	1.91635700	-0.13162800	2.01698200
N	0.06607700	-1.20245600	1.21610400
C	-0.67551400	-1.94680900	0.19703100
C	-1.73856700	-1.04974000	-0.44275200
N	-2.62560000	-0.46974500	0.57464200
C	-1.86816200	0.30187400	1.55778900
C	-0.80237000	-0.57868500	2.21530900
C	-3.85517700	0.06760600	0.11864500
C	-4.28176300	1.36124200	0.45212300
C	-5.53158700	1.84546500	0.03155600
C	-6.35102200	1.02947400	-0.74753600
C	-5.94791800	-0.26916700	-1.10316100
C	-4.71272200	-0.73749700	-0.66215000
H	7.28501300	1.42683100	-0.50645800
F	6.05905700	3.70984000	-0.67156500
H	3.48209700	3.41064400	-0.62958200

H	2.32039100	1.21696300	-0.46514000
H	6.14426600	-0.77088300	-0.31162400
H	0.00441400	-2.31526300	-0.56736000
H	-1.15920200	-2.81145200	0.67012000
H	-1.23597300	-0.25257500	-1.02012400
H	-2.32125100	-1.64891000	-1.14383200
H	-2.54869400	0.65422600	2.33780700
H	-1.38376100	1.18609000	1.10521900
H	-1.29496200	-1.37423000	2.78977900
H	-0.18015600	0.01250000	2.88587300
H	-3.64234200	2.01818300	1.03227800
C	-5.96821100	3.23901700	0.42535200
H	-7.31765300	1.40114000	-1.08167200
C	-6.85165200	-1.14452300	-1.94229300
H	-4.41888200	-1.75518000	-0.90307400
H	4.77127300	-2.44850700	-1.48869400
H	-6.87844700	3.53666700	-0.10440200
H	-5.19023500	3.97977000	0.20523900
H	-6.17328700	3.30259100	1.50190900
H	-6.43381000	-2.14806600	-2.06914600
H	-7.00325400	-0.71649100	-2.94140900
H	-7.84407500	-1.24649200	-1.48654400

5-(4-fluorophenyl)-4-(4-(3,5-dimethylphenyl)piperazine-1-carbonyl)-2*H*-1,2,3-triazole

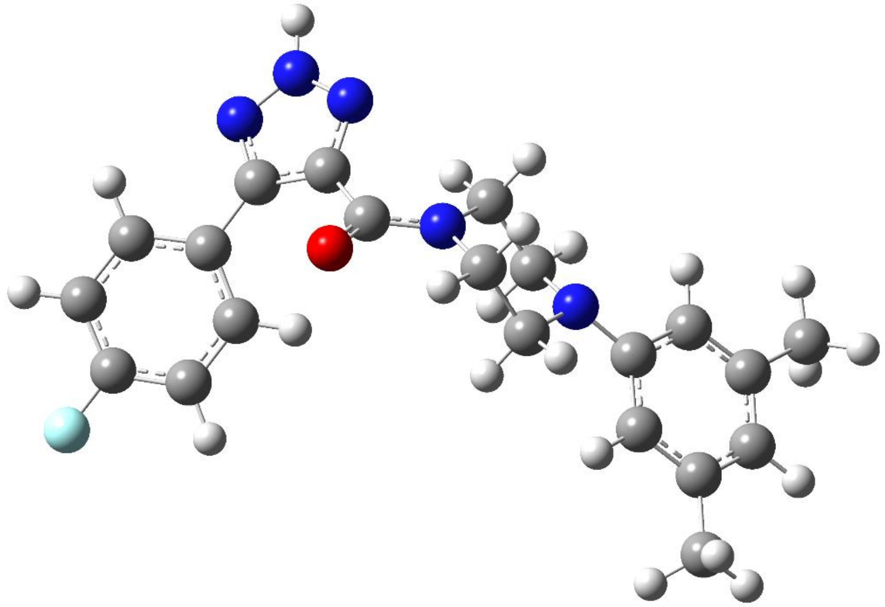

C	5.98244800	1.61178600	-0.72469600
C	5.14145900	2.70571000	-0.55398100
C	3.78002300	2.56040200	-0.31497200
C	3.24619200	1.27526400	-0.24298700
C	4.06434700	0.14514400	-0.40330200
C	5.43608400	0.33316900	-0.64715500

C	3.51574300	-1.21953500	-0.34864200
C	2.36426700	-1.75272000	0.29555100
N	2.29498400	-3.06491500	0.02939200
N	3.35334500	-3.28007900	-0.73494500
N	4.11458800	-2.22751200	-1.00320200
C	1.45459100	-1.10023200	1.29121600
O	1.95859400	-0.41201100	2.18604200
N	0.11407000	-1.32040200	1.19000400
C	-0.58950300	-1.96407200	0.07909900
C	-1.64128200	-1.01494800	-0.50065000
N	-2.56078300	-0.54291900	0.54325800
C	-1.83650000	0.13869700	1.61382400
C	-0.78496600	-0.79445500	2.21896900
C	-3.78247700	0.02792300	0.10503800
C	-4.22510700	1.28556500	0.53933900
C	-5.46622300	1.80028000	0.12950500
C	-6.25960900	1.05271600	-0.74004800
C	-5.83967200	-0.20830300	-1.19721800
C	-4.61375800	-0.70911600	-0.76568800
H	7.04068500	1.76583500	-0.90759400
F	5.66442300	3.95060500	-0.62360700
H	3.15518700	3.43914800	-0.19575300
H	2.18211500	1.15904700	-0.07169900
H	6.07773000	-0.53268200	-0.77172500
H	0.11795500	-2.24518700	-0.69815700
H	-1.07737200	-2.87602000	0.44706700
H	-1.12952200	-0.16278600	-0.98338000
H	-2.19925800	-1.54616400	-1.27297100
H	-2.54092700	0.41646600	2.40262300
H	-1.34484600	1.06192800	1.25732800
H	-1.28746000	-1.64031700	2.70628100
H	-0.18331200	-0.26557500	2.95706600
H	-3.60535500	1.89090300	1.19248900
C	-5.92222600	3.15175000	0.63270500
H	-7.21926900	1.44896900	-1.06605900
C	-6.71483400	-1.00849900	-2.13560500
H	-4.30678100	-1.70059900	-1.08645000
H	3.57179200	-4.20056700	-1.09456000
H	-6.81271100	3.49654600	0.09812200
H	-5.13901000	3.90947900	0.51148400
H	-6.16843700	3.11614200	1.70195400
H	-6.29770000	-2.00257800	-2.32407600
H	-6.82471300	-0.50297000	-3.10354300
H	-7.72476000	-1.13674900	-1.72768100

5-(4-fluorophenyl)-4-(4-(3,5-dimethylphenyl)piperazine-1-carbonyl)-3*H*-1,2,3-triazole

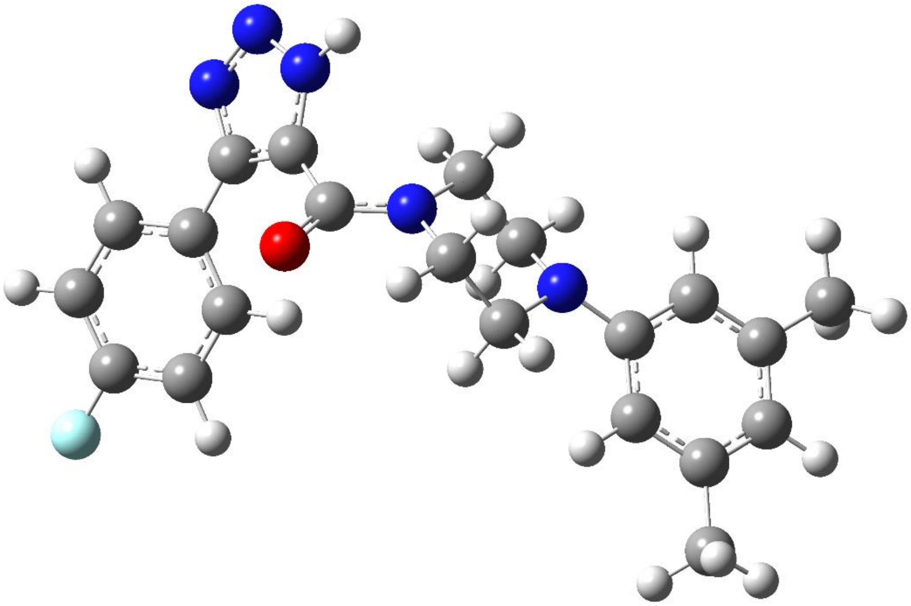

C	5.64106600	1.95301500	-0.82702400
C	4.74932500	2.94401600	-0.43271900
C	3.45270700	2.65022100	-0.02762700
C	3.03953900	1.31946200	-0.01609900
C	3.91405500	0.28850600	-0.39876900
C	5.21651000	0.62692300	-0.80674800
C	3.50403300	-1.12348500	-0.39663500
C	2.45829900	-1.77576500	0.25228100
N	2.59313700	-3.06915500	-0.14392500
N	3.63771500	-3.23468600	-0.96995900
N	4.18214800	-2.05912000	-1.13349900
C	1.49584300	-1.33284500	1.31331400
O	1.94318600	-0.80340800	2.33377800
N	0.17361800	-1.58311200	1.10826600
C	-0.44740700	-1.97312100	-0.16085200
C	-1.47419800	-0.92225400	-0.59221300
N	-2.46320900	-0.68951000	0.46747900
C	-1.81812400	-0.24329000	1.70087100
C	-0.79508300	-1.28290000	2.16544100
C	-3.66687400	-0.04599500	0.07881000
C	-4.14853500	1.10223900	0.72259800
C	-5.37075000	1.68475700	0.34892000
C	-6.10299500	1.11874300	-0.69460200
C	-5.64113500	-0.02757200	-1.36339300
C	-4.43598000	-0.60176000	-0.96512100
H	6.64563200	2.22108400	-1.13711200
F	5.15461300	4.23413600	-0.44670200
H	2.78236500	3.45199700	0.26350300
H	2.02222900	1.09289600	0.28304900
H	5.89841200	-0.16109000	-1.10712000

H	0.31377200	-2.07176400	-0.93475700
H	-0.94351000	-2.94314200	-0.03093100
H	-0.94539000	0.01405100	-0.84643600
H	-1.97322100	-1.27688800	-1.49498700
H	-2.57521700	-0.13525000	2.48217900
H	-1.31626500	0.73318900	1.57631800
H	-1.31400300	-2.21305600	2.42895400
H	-0.24761800	-0.92080700	3.03481000
H	-3.57323900	1.56823200	1.51556000
C	-5.87401300	2.91025200	1.07840500
H	-7.04779400	1.56837000	-0.99340800
C	-6.44437300	-0.62537600	-2.49672300
H	-4.09691800	-1.51127600	-1.45319600
H	2.04701700	-3.87456800	0.13929000
H	-6.72711000	3.36035200	0.56131600
H	-5.09111400	3.67254100	1.16848200
H	-6.19642500	2.66203300	2.09804900
H	-6.06550600	-1.61202400	-2.78098700
H	-6.40736300	0.01371100	-3.38862000
H	-7.50107600	-0.73209100	-2.22508200

Tautomers of **7o**

5-(4-fluorophenyl)-4-(4-(3,5-dimethoxyphenyl)piperazine-1-carbonyl)-1*H*-1,2,3-triazole

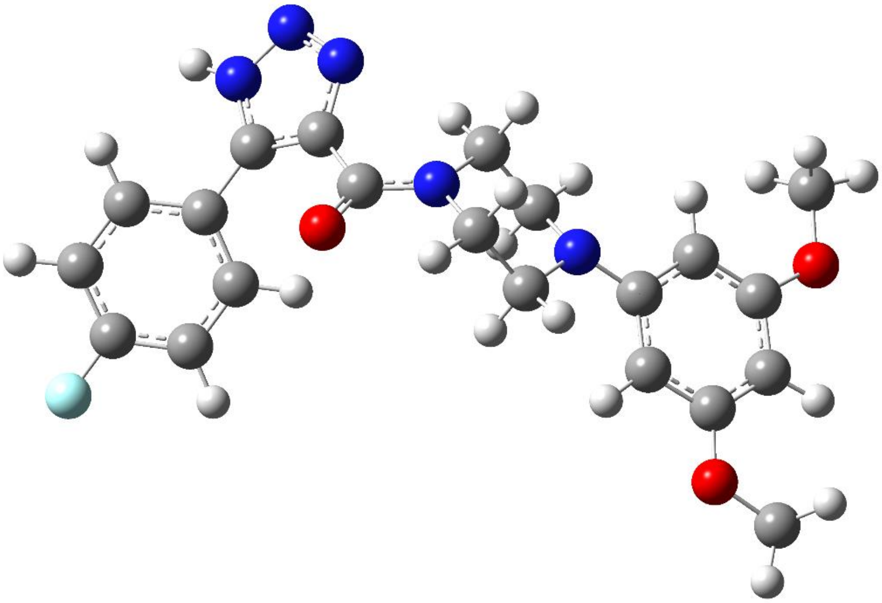

C	6.55744500	1.52500800	-0.70074100
C	5.72546400	2.63099300	-0.82688600
C	4.33967500	2.52705200	-0.76314500
C	3.77150800	1.27148900	-0.56998500
C	4.58033900	0.12960400	-0.43886400
C	5.97703200	0.27377000	-0.50430100
C	3.97679400	-1.19541300	-0.25725600
C	2.84728900	-1.66856100	0.41234200
N	2.76752800	-3.02160700	0.22985300
N	3.76280100	-3.42017400	-0.51351900
N	4.48900200	-2.32445700	-0.81004100
C	1.96409700	-0.90610800	1.34598700
O	2.46826300	-0.03969700	2.07124300
N	0.63748900	-1.22282600	1.39295600
C	-0.11052400	-2.03012300	0.42801000
C	-1.19597000	-1.18225400	-0.24094400
N	-2.07444200	-0.55840900	0.75872600
C	-1.31237500	0.26750900	1.69443000
C	-0.22535800	-0.56751500	2.37608900
C	-3.31602900	-0.05980000	0.29992000
C	-3.74931000	1.23522900	0.59787100
C	-5.01572700	1.67219200	0.16942800
C	-5.85044900	0.84113000	-0.57082800
C	-5.40461200	-0.45919000	-0.87190500
C	-4.16235600	-0.91744900	-0.44336900
H	7.63404900	1.64808300	-0.74703800
F	6.28001100	3.84700600	-1.01629500
H	3.72568200	3.41466400	-0.87111500

H	2.69202600	1.17765800	-0.53306200
H	6.62239900	-0.59034200	-0.37837400
H	0.56133000	-2.43146700	-0.32707400
H	-0.57421400	-2.87314100	0.95643300
H	-0.71366400	-0.41030200	-0.86704700
H	-1.78056800	-1.82486100	-0.90039100
H	-1.98680400	0.64689300	2.46670500
H	-0.84710500	1.13476900	1.19259600
H	-0.69765100	-1.34351400	2.99232700
H	0.39635000	0.06309000	3.01021300
H	-3.13019200	1.94073900	1.13683500
O	-5.32935600	2.95463800	0.52146600
H	-6.82728500	1.14940800	-0.92042400
O	-6.29227300	-1.21235200	-1.58829200
H	-3.85487100	-1.93660300	-0.63541100
C	-6.58181500	3.48276100	0.09512200
C	-5.92210200	-2.54046900	-1.94527300
H	-6.61880000	4.50570100	0.47305400
H	-7.42019900	2.91050800	0.51050800
H	-6.65925500	3.49492600	-0.99884900
H	-6.75719700	-2.93705300	-2.52491700
H	-5.76667800	-3.16596200	-1.05771600
H	-5.01362600	-2.55178200	-2.55962000
H	5.28835300	-2.41199700	-1.42636300

5-(4-fluorophenyl)-4-(4-(3,5-dimethoxyphenyl)piperazine-1-carbonyl)-2*H*-1,2,3-triazole

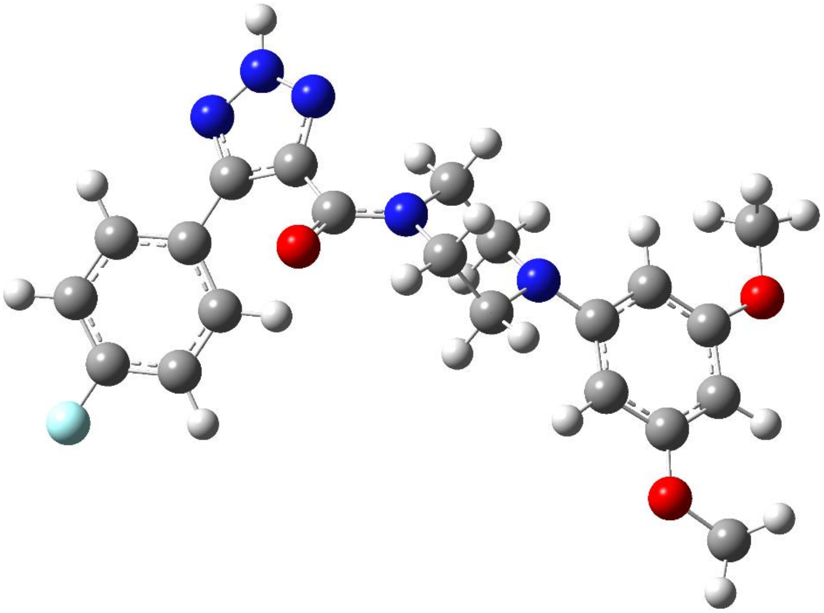

C	6.39831800	1.66754500	-0.95902300
C	5.52915000	2.74827100	-0.86062800
C	4.17973900	2.58649500	-0.56969000
C	3.68770100	1.29833300	-0.36923000

C	4.53525500	0.18179300	-0.45413500
C	5.89360500	0.38594200	-0.75368500
C	4.02911700	-1.18734100	-0.26536400
C	2.91092700	-1.69297700	0.45520100
N	2.87207300	-3.02461000	0.30440500
N	3.91542900	-3.27712300	-0.46909000
N	4.63890800	-2.23207900	-0.84825500
C	2.01014100	-0.98077900	1.41757800
O	2.51817200	-0.20658400	2.23652300
N	0.67376800	-1.24162200	1.37309100
C	-0.04067900	-1.99538700	0.34195300
C	-1.12981000	-1.12469700	-0.28973600
N	-2.03502900	-0.58342900	0.73340700
C	-1.30228500	0.20175300	1.72526000
C	-0.21273100	-0.65236700	2.37773800
C	-3.28154600	-0.08473600	0.28673100
C	-3.74278200	1.18483900	0.64474500
C	-5.01249100	1.61897900	0.22284400
C	-5.82216700	0.81050800	-0.56851700
C	-5.34807500	-0.46442800	-0.92913700
C	-4.10205200	-0.92004700	-0.50864300
H	7.44602800	1.83440200	-1.18596600
F	6.01142700	3.99617300	-1.05580400
H	3.53159500	3.45436500	-0.51012100
H	2.63272900	1.16827600	-0.15680900
H	6.55807300	-0.46899800	-0.82079400
H	0.65369600	-2.32687400	-0.42718400
H	-0.49622500	-2.88280700	0.80038300
H	-0.65208300	-0.30529100	-0.85617400
H	-1.69179200	-1.73329800	-0.99913600
H	-1.99445700	0.52637200	2.50658900
H	-0.84428200	1.10309400	1.27991500
H	-0.68111900	-1.46595000	2.94697700
H	0.39233600	-0.04850200	3.05281600
H	-3.14359700	1.87353200	1.22649900
O	-5.35516400	2.87584400	0.63456600
H	-6.80081600	1.11760700	-0.91403200
O	-6.21266400	-1.19709400	-1.69312100
H	-3.77151900	-1.92209100	-0.74724300
C	-6.61205800	3.40150800	0.21805200
C	-5.81261200	-2.49852200	-2.11122700
H	-6.67294400	4.40372400	0.64517400
H	-7.44452400	2.79424700	0.59372100
H	-6.67606200	3.46611600	-0.87494200
H	-6.63248300	-2.88072700	-2.72142400
H	-5.65661600	-3.16499300	-1.25411600

H	-4.89626000	-2.46190000	-2.71270700
H	4.15071300	-4.21925800	-0.75436700

5-(4-fluorophenyl)-4-(4-(3,5-dimethoxyphenyl)piperazine-1-carbonyl)-3*H*-1,2,3-triazole

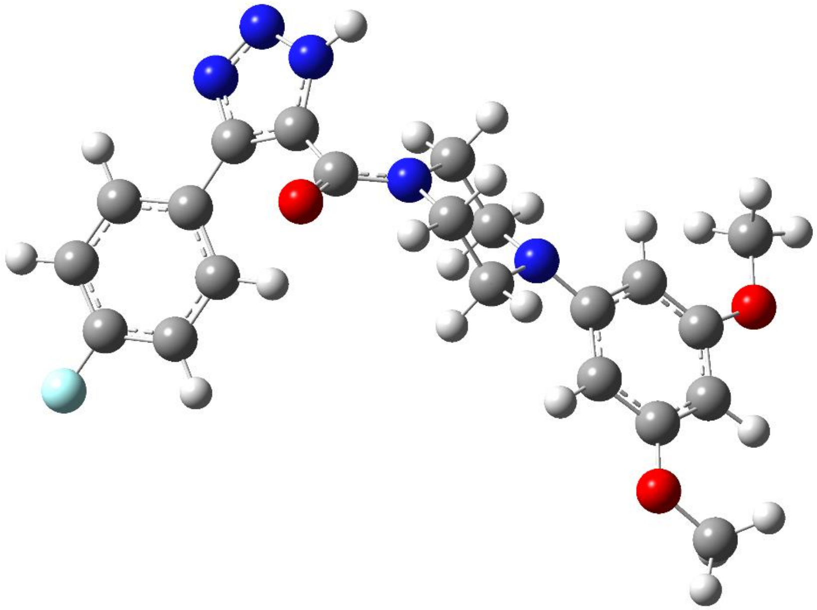

C	6.11415900	1.87524200	-1.17642500
C	5.23905900	2.91093600	-0.86961800
C	3.96383800	2.67453200	-0.37011400
C	3.55554600	1.35729300	-0.17074300
C	4.41326900	0.28315600	-0.46172800
C	5.69468900	0.56368900	-0.96912200
C	4.00850700	-1.11686800	-0.26421600
C	2.98093800	-1.68288300	0.48775900
N	3.11454500	-3.01656400	0.25867300
N	4.14063100	-3.28442300	-0.56321900
N	4.67410900	-2.13840100	-0.89014900
C	2.03949500	-1.10522300	1.50173000
O	2.50573900	-0.42475300	2.41916100
N	0.71558100	-1.39690500	1.37607900
C	0.06597400	-1.99493200	0.20665100
C	-1.00392800	-1.04995300	-0.34704400
N	-1.96446600	-0.67537800	0.69849700
C	-1.29356000	-0.02437000	1.82166800
C	-0.22650100	-0.95286700	2.40688600
C	-3.20492500	-0.14501200	0.26919800
C	-3.70868500	1.05845400	0.76870300
C	-4.97257000	1.51852000	0.35632900
C	-5.73173700	0.80366900	-0.56468100
C	-5.21351100	-0.40400700	-1.06794600
C	-3.97348000	-0.88624300	-0.65916200
H	7.10251600	2.09867900	-1.56421600

F	5.63965400	4.18772500	-1.06531300
H	3.30584800	3.50859200	-0.15020600
H	2.55625400	1.17445300	0.20697400
H	6.36456600	-0.25753600	-1.19902100
H	0.80363600	-2.19363600	-0.57057200
H	-0.39709100	-2.94479000	0.50226400
H	-0.51110300	-0.15303900	-0.76247200
H	-1.52031200	-1.55309500	-1.16527800
H	-2.02726000	0.18638600	2.60396400
H	-0.82716300	0.93201400	1.52545300
H	-0.71033000	-1.83959900	2.83540200
H	0.33903900	-0.44385600	3.18640300
H	-3.14845000	1.67673200	1.45860200
O	-5.36182900	2.70318800	0.91385700
H	-6.70464400	1.13308900	-0.90591100
O	-6.03020000	-1.04820300	-1.95413600
H	-3.60965900	-1.84280800	-1.00958000
C	-6.62110800	3.24723600	0.52908700
C	-5.58275700	-2.27677000	-2.51914100
H	-6.72411300	4.18184400	1.08264200
H	-7.44538900	2.57403700	0.79394500
H	-6.65517100	3.45504100	-0.54723000
H	-6.37030000	-2.59383400	-3.20456800
H	-5.44111500	-3.04430100	-1.74843800
H	-4.64659500	-2.14445100	-3.07497100
H	2.58120900	-3.78121200	0.65599900

Tautomers of **7q**
5-(3-chlorophenyl)-4-(4-(3,5-dimethylphenyl)piperazine-1-carbonyl)-1*H*-1,2,3-triazole

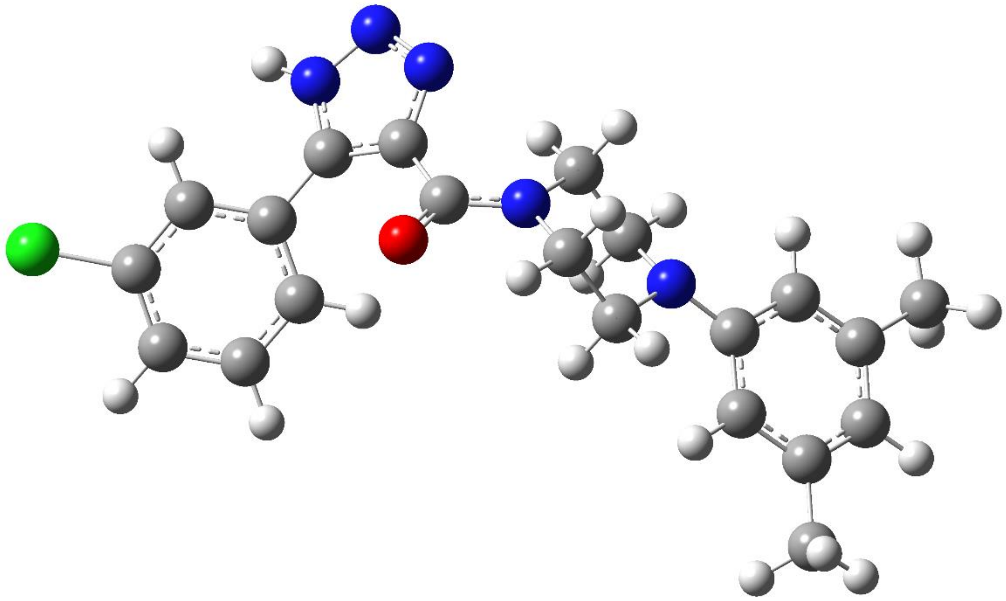

C	5.76325800	1.41053200	-0.46194100
C	5.02238400	2.58404500	-0.58676100
C	3.62842100	2.49646600	-0.59343900
C	2.99114200	1.26349800	-0.48076000
C	3.74754800	0.08756900	-0.35546400
C	5.14984400	0.16638100	-0.34388600
C	3.08745800	-1.22021300	-0.25750400
C	1.91379600	-1.67378000	0.34462200
N	1.79165300	-3.01385400	0.10236300
N	2.80169000	-3.42014800	-0.61721300
N	3.58080600	-2.34428000	-0.83630300
C	1.02200700	-0.91622600	1.27535900
O	1.53124200	-0.10767500	2.06115500
N	-0.31661600	-1.17582700	1.24900500
C	-1.04960800	-1.92267200	0.22538700
C	-2.10107900	-1.02410500	-0.43126300
N	-2.99709600	-0.43527300	0.57302900
C	-2.24805400	0.33770700	1.56164500
C	-1.19348200	-0.54382200	2.23565900
C	-4.21893500	0.10542600	0.10029800
C	-4.64409500	1.40194100	0.42422600
C	-5.88671700	1.88996800	-0.01296200
C	-6.70005800	1.07484500	-0.79928700
C	-6.29805600	-0.22659000	-1.14578000
C	-5.07028200	-0.69866000	-0.68827100
Cl	7.52362300	1.49778200	-0.44139800
H	5.52169000	3.54257700	-0.67572900
H	3.03777200	3.40170500	-0.69428500

H	1.90864800	1.20984600	-0.50245700
H	5.75714100	-0.72367500	-0.21945400
H	-0.36255600	-2.29825200	-0.52926900
H	-1.54245800	-2.78233100	0.69787500
H	-1.58817600	-0.23213800	-1.00672800
H	-2.67814800	-1.62428700	-1.13607500
H	-2.93615500	0.69612000	2.33216300
H	-1.75489800	1.21797000	1.11088600
H	-1.69531800	-1.33482400	2.80825000
H	-0.57639900	0.04813000	2.91030800
H	-4.00904100	2.05827400	1.00980800
C	-6.32205700	3.28664100	0.37102400
H	-7.66097300	1.44937500	-1.14643100
C	-7.19520400	-1.10095100	-1.99297400
H	-4.77801600	-1.71837300	-0.92237500
H	4.40208900	-2.43826300	-1.42222300
H	-7.22564300	3.58575800	-0.16918100
H	-5.53902200	4.02360600	0.15615800
H	-6.53793400	3.35506500	1.44516400
H	-6.77829000	-2.10552100	-2.11456400
H	-7.33592400	-0.67364800	-2.99398000
H	-8.19235200	-1.20034200	-1.54704700

5-(3-chlorophenyl)-4-(4-(3,5-dimethylphenyl)piperazine-1-carbonyl)-2*H*-1,2,3-triazole

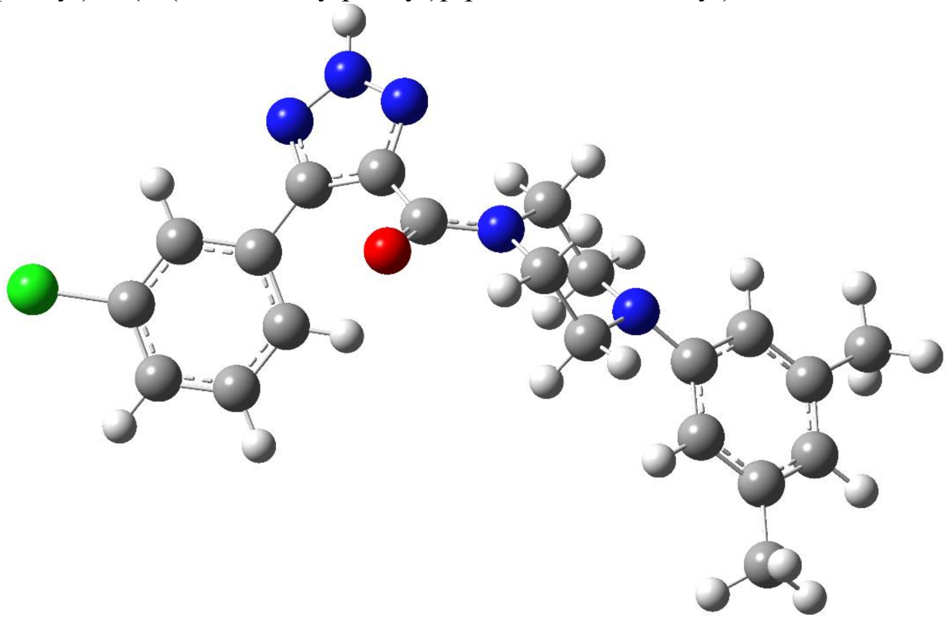

C	5.63222800	1.54846600	-0.49847300
C	4.85216100	2.68997900	-0.31941000
C	3.48195300	2.52958200	-0.10652500
C	2.90800600	1.25979600	-0.07139500
C	3.70389400	0.11756400	-0.24648600
C	5.08334300	0.27146900	-0.46457500

C	3.13042500	-1.23883800	-0.23526400
C	1.95247500	-1.76589500	0.36369300
N	1.87125700	-3.07035500	0.06646100
N	2.94786300	-3.28579200	-0.67265800
N	3.73259500	-2.24142400	-0.89437600
C	1.02572200	-1.12092100	1.34915200
O	1.51749200	-0.45886300	2.26998400
N	-0.31399200	-1.31987500	1.20940800
C	-0.99820900	-1.93419600	0.06988300
C	-2.02539500	-0.96218300	-0.51615200
N	-2.96401300	-0.49913400	0.51467500
C	-2.25749100	0.15385000	1.61458600
C	-1.23152100	-0.80285100	2.22676500
C	-4.16828800	0.09447000	0.05858400
C	-4.60609400	1.34937500	0.50560500
C	-5.83095300	1.88670400	0.07662100
C	-6.61219200	1.16523200	-0.82544200
C	-6.19645700	-0.09214700	-1.29612900
C	-4.98721200	-0.61603800	-0.84512400
Cl	7.36824700	1.72483900	-0.76963000
H	5.30282900	3.67596400	-0.34676100
H	2.85692300	3.40723800	0.02766100
H	1.83964100	1.16180600	0.08131000
H	5.71136800	-0.60097900	-0.60094800
H	-0.27590600	-2.20859100	-0.69602900
H	-1.50458400	-2.84720100	0.40886800
H	-1.49308400	-0.10723100	-0.97067500
H	-2.57063100	-1.47271300	-1.31119500
H	-2.97709400	0.42516800	2.39183200
H	-1.74699500	1.07763000	1.28730900
H	-1.75500800	-1.65193600	2.68535200
H	-0.64206200	-0.29520500	2.98920300
H	-3.99478100	1.93499700	1.18415200
C	-6.28390000	3.23308700	0.59593200
H	-7.55893600	1.57932800	-1.16668300
C	-7.05819500	-0.86371300	-2.27026200
H	-4.68440800	-1.60507100	-1.17724000
H	3.16146300	-4.20140300	-1.04777800
H	-7.14223100	3.61014200	0.03131800
H	-5.48172100	3.97773800	0.53225400
H	-6.58038600	3.17328600	1.65133800
H	-6.64813700	-1.85886600	-2.46829400
H	-7.13927300	-0.33837100	-3.23045700
H	-8.07890800	-0.98779100	-1.88876900

5-(3-chlorophenyl)-4-(4-(3,5-dimethylphenyl)piperazine-1-carbonyl)-3*H*-1,2,3-triazole

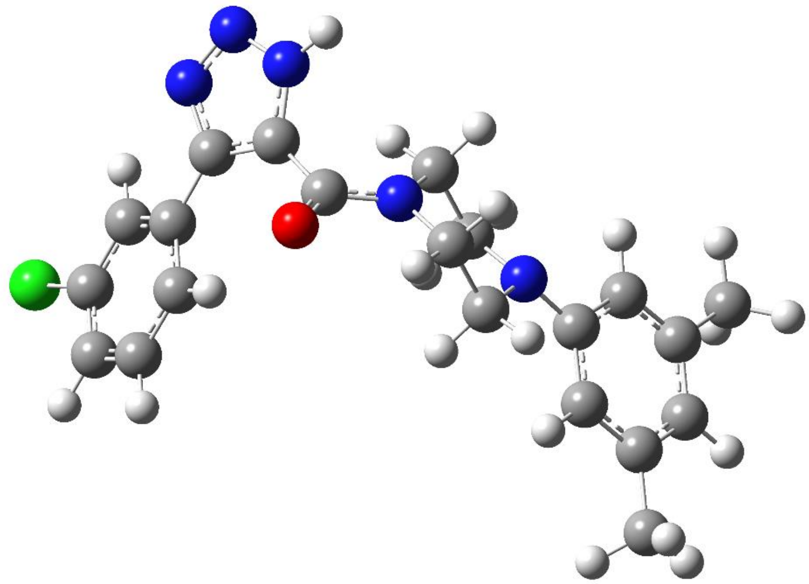

C	4.59599400	2.27333300	-0.13144500
C	4.19350200	2.69988600	1.13345900
C	3.45427000	1.81847800	1.92392700
C	3.12466600	0.54452000	1.46265000
C	3.52724700	0.13079500	0.18229700
C	4.27589900	1.01137200	-0.61815800
C	3.20491100	-1.20327000	-0.35046700
C	2.18664800	-2.10187000	-0.03958400
N	2.39650500	-3.13300500	-0.89829700
N	3.45773400	-2.91674300	-1.69322600
N	3.93941500	-1.74866700	-1.37186200
C	1.11944500	-2.12716200	1.01717500
O	1.45569100	-2.16894300	2.20517100
N	-0.17171400	-2.13759500	0.59743100
C	-0.63907900	-1.89796600	-0.77130700
C	-1.56561200	-0.67992500	-0.80624200
N	-2.67280300	-0.83758300	0.14542200
C	-2.17592100	-1.01661400	1.50805400
C	-1.25720100	-2.23897600	1.57742600
C	-3.79503900	0.01059400	-0.04286700
C	-4.31414400	0.81180100	0.98304100
C	-5.45905500	1.60005400	0.77918500
C	-6.07363300	1.59592000	-0.47253600
C	-5.57125700	0.80762400	-1.52221400
C	-4.44560200	0.01948500	-1.29488600
Cl	5.53386400	3.36954000	-1.15128600
H	4.45325600	3.69032500	1.49073700
H	3.14002900	2.12901500	2.91606100
H	2.57349300	-0.13644400	2.10098300
H	4.59553700	0.70391500	-1.60655500

H	0.21084200	-1.72615300	-1.43142000
H	-1.18029400	-2.78595900	-1.12053200
H	-0.97695100	0.22618400	-0.57662300
H	-1.95871800	-0.57300800	-1.81814400
H	-3.02250900	-1.18791300	2.17831700
H	-1.62963600	-0.12762700	1.87105100
H	-1.83524000	-3.14477800	1.35640500
H	-0.81360000	-2.33170700	2.56795700
H	-3.82796900	0.84604400	1.95242500
C	-6.00268500	2.44187600	1.91198400
H	-6.95786400	2.20797800	-0.63855800
C	-6.25142000	0.81282800	-2.87280400
H	-4.07963000	-0.62213000	-2.09166000
H	1.88307400	-4.00361900	-0.97594700
H	-6.81825300	3.08822000	1.57326200
H	-5.22354200	3.08007600	2.34610100
H	-6.38943500	1.81407400	2.72500300
H	-5.82792900	0.05435600	-3.53845400
H	-6.14465000	1.78704400	-3.36711000
H	-7.32679600	0.61994700	-2.77921400

Tautomers of **7r**

5-(3-chlorophenyl)-4-(4-(3,5-dimethoxyphenyl)piperazine-1-carbonyl)-1*H*-1,2,3-triazole

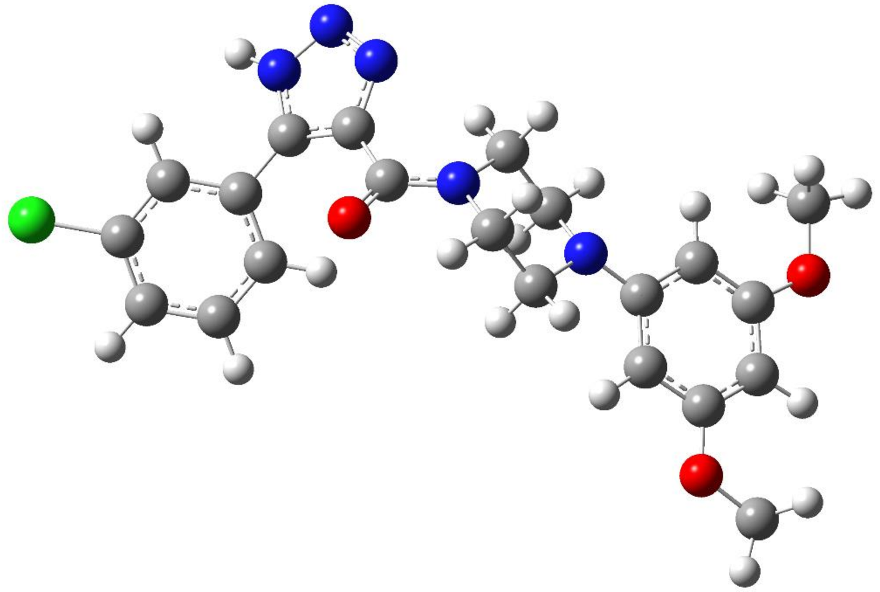

C	6.18263700	1.47952400	-0.63388400
C	5.40032000	2.61666700	-0.82449000
C	4.01024500	2.48393100	-0.79399700
C	3.41745500	1.24212800	-0.58082100
C	4.21537400	0.10284600	-0.39079300
C	5.61404300	0.22760800	-0.41554900
C	3.60059600	-1.21515100	-0.18725800
C	2.46019500	-1.66126900	0.48078000
N	2.37024000	-3.01692600	0.32845100
N	3.36942000	-3.44075300	-0.39625100
N	4.10952400	-2.36023100	-0.70849100
C	1.57741500	-0.86581100	1.38793700
O	2.08798600	0.01253300	2.09376100
N	0.24744800	-1.16542400	1.43189500
C	-0.50185200	-1.99417800	0.48581200
C	-1.57575100	-1.15724900	-0.21445600
N	-2.45418900	-0.49679800	0.76133100
C	-1.68996000	0.35083700	1.67570300
C	-0.61528700	-0.47289200	2.38961200
C	-3.68986000	-0.00328800	0.28140500
C	-4.11400400	1.30391300	0.53587800
C	-5.37520100	1.73678900	0.08822600
C	-6.21341200	0.88891100	-0.62868400
C	-5.77657800	-0.42381400	-0.88611000
C	-4.53974800	-0.87771200	-0.43777600
Cl	7.93914500	1.62502400	-0.65788600
H	5.86522800	3.58199400	-0.99154900
H	3.38751100	3.36026700	-0.94474300

H	2.33716800	1.15231400	-0.57416600
H	6.25337100	-0.63141300	-0.24333100
H	0.17113700	-2.42322600	-0.25289600
H	-0.97602700	-2.81712500	1.03597400
H	-1.08328100	-0.40854900	-0.86052500
H	-2.16206300	-1.81432500	-0.85793600
H	-2.36537100	0.76037100	2.43154100
H	-1.21348500	1.19759500	1.15000900
H	-1.09850300	-1.22554300	3.02593900
H	0.00827100	0.17074400	3.00864000
H	-3.49135500	2.02118700	1.05487600
O	-5.68009000	3.03222200	0.39765500
H	-7.18641800	1.19325300	-0.99214900
O	-6.66723500	-1.19273400	-1.58171200
H	-4.23960900	-1.90489100	-0.59545300
C	-6.92693300	3.55590200	-0.05007400
C	-6.30658800	-2.53481300	-1.89334300
H	-6.95715900	4.59079800	0.29442000
H	-7.77113800	3.00388100	0.38061500
H	-7.00063900	3.53323500	-1.14414000
H	-7.14328800	-2.94392500	-2.46185900
H	-6.15837600	-3.13155500	-0.98500600
H	-5.39668100	-2.57365800	-2.50447000
H	4.91422300	-2.46936700	-1.31460800

5-(3-chlorophenyl)-4-(4-(3,5-dimethoxyphenyl)piperazine-1-carbonyl)-2*H*-1,2,3-triazole

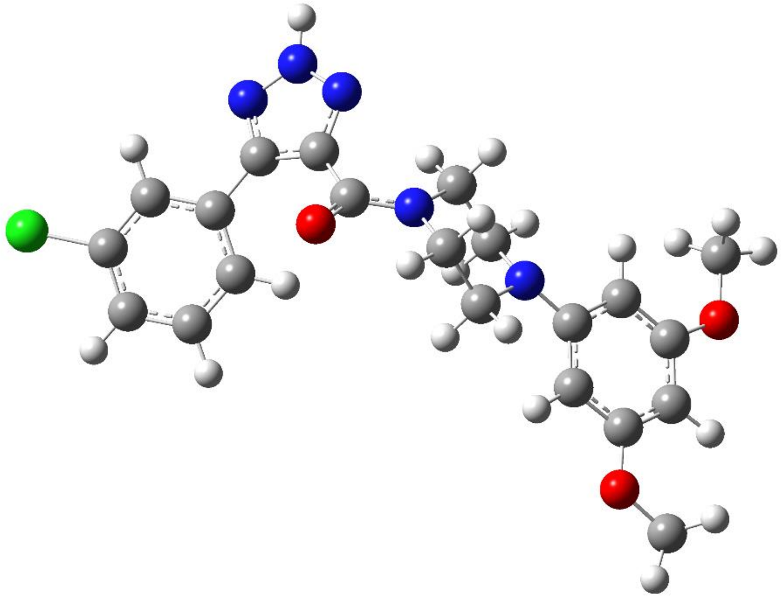

C	6.04206900	1.60565800	-0.72981800
C	5.22452700	2.73162700	-0.64325100
C	3.86692200	2.54626800	-0.37660600
C	3.34192600	1.26731000	-0.19894100

C	4.17539700	0.14166500	-0.28223300
C	5.54225100	0.32022500	-0.55374600
C	3.65154100	-1.22479700	-0.11967900
C	2.51061400	-1.72558500	0.56644800
N	2.46356800	-3.05472400	0.40050800
N	3.52439400	-3.30929500	-0.34921200
N	4.26706600	-2.26862000	-0.69734500
C	1.59293100	-1.01459700	1.51448100
O	2.09032200	-0.26171900	2.35912000
N	0.25545000	-1.25492300	1.42728800
C	-0.43862800	-1.98392800	0.36466000
C	-1.49844000	-1.08973700	-0.28350700
N	-2.42570500	-0.55344700	0.72229600
C	-1.71225500	0.20758600	1.74654700
C	-0.65127700	-0.66910100	2.41592500
C	-3.65348600	-0.03326600	0.24892700
C	-4.11193100	1.23459700	0.61661600
C	-5.36418000	1.69007500	0.16591300
C	-6.15853200	0.90504500	-0.66364600
C	-5.68716100	-0.36815300	-1.03376400
C	-4.45892600	-0.84495400	-0.58533600
Cl	7.76293900	1.81405500	-1.06664700
H	5.63731700	3.72477900	-0.78192900
H	3.21313200	3.41082300	-0.31313500
H	2.28197700	1.14775700	-0.00760200
H	6.19883800	-0.53922300	-0.62040800
H	0.27311000	-2.31233800	-0.38983500
H	-0.91782900	-2.87225200	0.79632400
H	-0.99485300	-0.26779600	-0.82320700
H	-2.04710800	-1.68015400	-1.01818300
H	-2.42223900	0.52815100	2.51336600
H	-1.23185700	1.11025400	1.32835000
H	-1.14444300	-1.48491400	2.96048500
H	-0.05768300	-0.08251300	3.11599500
H	-3.52329000	1.90629300	1.22826300
O	-5.70564900	2.94299700	0.59036400
H	-7.12325300	1.22907100	-1.03206600
O	-6.53600200	-1.07733300	-1.83656200
H	-4.13243400	-1.84617900	-0.83259500
C	-6.94351800	3.49046000	0.14583700
C	-6.13540700	-2.37369000	-2.26968600
H	-7.00617300	4.48562300	0.58890100
H	-7.79363100	2.88638100	0.48537300
H	-6.97375500	3.57508100	-0.94725700
H	-6.93995700	-2.73515100	-2.91206700
H	-6.01139800	-3.05935200	-1.42250900

H	-5.20102400	-2.33340200	-2.84250400
H	3.75720100	-4.25061900	-0.63972100

5-(3-chlorophenyl)-4-(4-(3,5-dimethoxyphenyl)piperazine-1-carbonyl)-3*H*-1,2,3-triazole

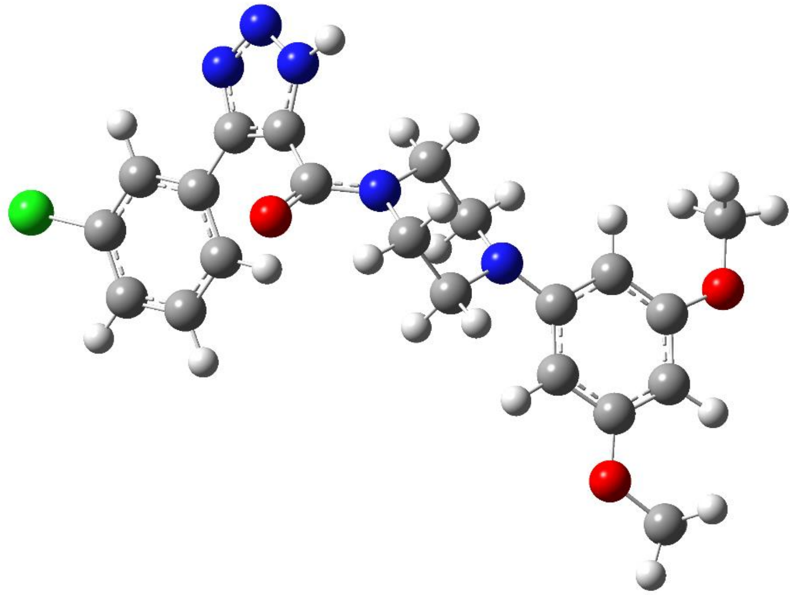

C	5.69321600	1.88733600	-0.70654100
C	4.84554200	2.92044400	-0.30950400
C	3.57341700	2.58785000	0.15909900
C	3.16213000	1.25805500	0.22966800
C	4.02664500	0.22501300	-0.16611200
C	5.30760900	0.55339400	-0.64240000
C	3.63516400	-1.19166800	-0.11146600
C	2.59171500	-1.83312800	0.55171000
N	2.74836500	-3.13742500	0.20614600
N	3.80316900	-3.31934700	-0.60504500
N	4.33308000	-2.14447300	-0.80622200
C	1.60795300	-1.36744900	1.58499600
O	2.03728200	-0.82263600	2.60478900
N	0.29034200	-1.61397500	1.35385000
C	-0.30085600	-2.03763000	0.08131100
C	-1.31553900	-0.99819100	-0.40236900
N	-2.32916400	-0.73679200	0.62709600
C	-1.71497600	-0.26006600	1.86497000
C	-0.70212000	-1.28535700	2.38023600
C	-3.53062500	-0.12004700	0.20221300
C	-4.04284500	1.01553600	0.83454200
C	-5.27054000	1.56148000	0.41791400
C	-5.98422900	1.00163900	-0.63691200
C	-5.45676200	-0.13681100	-1.27386800
C	-4.25297800	-0.70390300	-0.86545100
Cl	7.30767900	2.28108600	-1.30510300

H	5.16919400	3.95381800	-0.36702400
H	2.89492500	3.37792400	0.46654800
H	2.16351900	1.03144200	0.58454700
H	5.98453200	-0.23274800	-0.95417400
H	0.47823400	-2.15669100	-0.67163700
H	-0.80016800	-3.00393300	0.22521800
H	-0.78034500	-0.06896900	-0.66711400
H	-1.79154000	-1.37542200	-1.30797400
H	-2.49083800	-0.13726900	2.62490900
H	-1.21459500	0.71507700	1.72815500
H	-1.22620200	-2.20823600	2.65844700
H	-0.17463700	-0.89845100	3.25124500
H	-3.51426700	1.51756400	1.63470000
O	-5.67156500	2.66725200	1.11221700
H	-6.92798900	1.40121400	-0.98481700
O	-6.22834000	-0.62852800	-2.28891100
H	-3.88667800	-1.61091500	-1.32712700
C	-6.89445000	3.29332100	0.73458500
C	-5.76585700	-1.77252400	-3.00058100
H	-7.01309400	4.14551400	1.40556200
H	-7.74612100	2.61295400	0.85548400
H	-6.85895800	3.64773800	-0.30273400
H	-6.51555000	-1.96865300	-3.76875200
H	-5.68171900	-2.64683600	-2.34361000
H	-4.79630300	-1.58283100	-3.47689900
H	2.20883400	-3.93914500	0.51202700
